# Highly diastereoselective preparation of chiral NHC-boranes stereogenic at the boron atom†
†Electronic supplementary information (ESI) available: Experimental details, spectroscopic data and theoretical investigations; X-ray data for compounds **4a**, **4b** and **4d**. CCDC 1856403, 1857908 and 1857909. For ESI and crystallographic data in CIF or other electronic format see DOI: 10.1039/c9sc01454c


**DOI:** 10.1039/c9sc01454c

**Published:** 2019-05-24

**Authors:** Clara Aupic, Amel Abdou Mohamed, Carlotta Figliola, Paola Nava, Béatrice Tuccio, Gaëlle Chouraqui, Jean-Luc Parrain, Olivier Chuzel

**Affiliations:** a Aix Marseille Univ , CNRS , Centrale Marseille, iSm2 , Marseille , France . Email: olivier.chuzel@univ-amu.fr; b Aix Marseille Univ , CNRS , ICR , Marseille , France

## Abstract

Stereogenic main group elements are clearly generating interest in the enantioselective catalysis field. Surprisingly, while chiral organoboron reagents are very useful in stereoselective transformations, few scaffolds stereogenic at boron and configurationally stable have been reported to date. Herein, we describe an original library of chiral NHC-boranes, stereogenic at the boron atom, that has been prepared in only a few steps and in good yields (up to 93%). Key steps involve a chlorination/arylation sequence in the presence of simple Grignard reagents from bicyclic NHC-boranes. The high and unprecedented diastereoselectivity observed during the second step (up to 99 : 1 dr) has been rationalized through a plausible S_RN_1 mechanism thanks to EPR observations and DFT calculations.

## Abstract 2

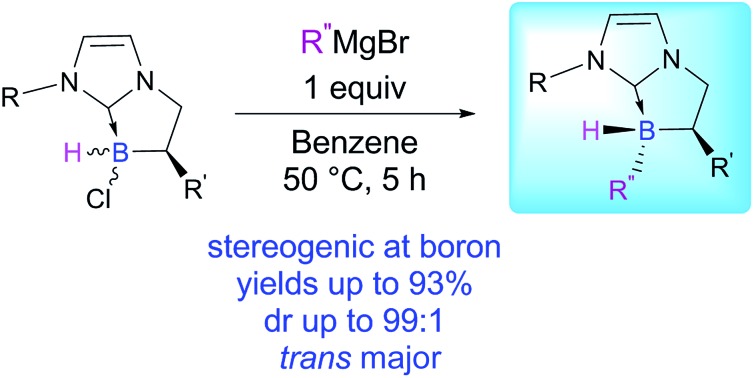
Stereogenic main group elements, a promising strategy for metal-free enantioselective catalysis.

## Introduction

When it comes to enantioselective catalysis, the literature on the subject is widely dominated by transition metal catalysts. Nevertheless, due to their inherent cost, rarity and toxicity, alternative solutions are needed, and chemists worldwide have focused on developing more environmentally benign systems based on abundant materials and low toxicity metal-, organo- or bio-catalysis.^1^ In this respect, main-group elements offer great potential and have recently been presented as the fourth pillar of catalysis by Melen^2^ (after bio-, organo- and metal catalysis as defined by List).^3^ Particularly, as with transition metal catalysis, p-block elements allow the activation of various chemical bonds and, consequently enable chemical transformations.^4^

**Fig. 1:**
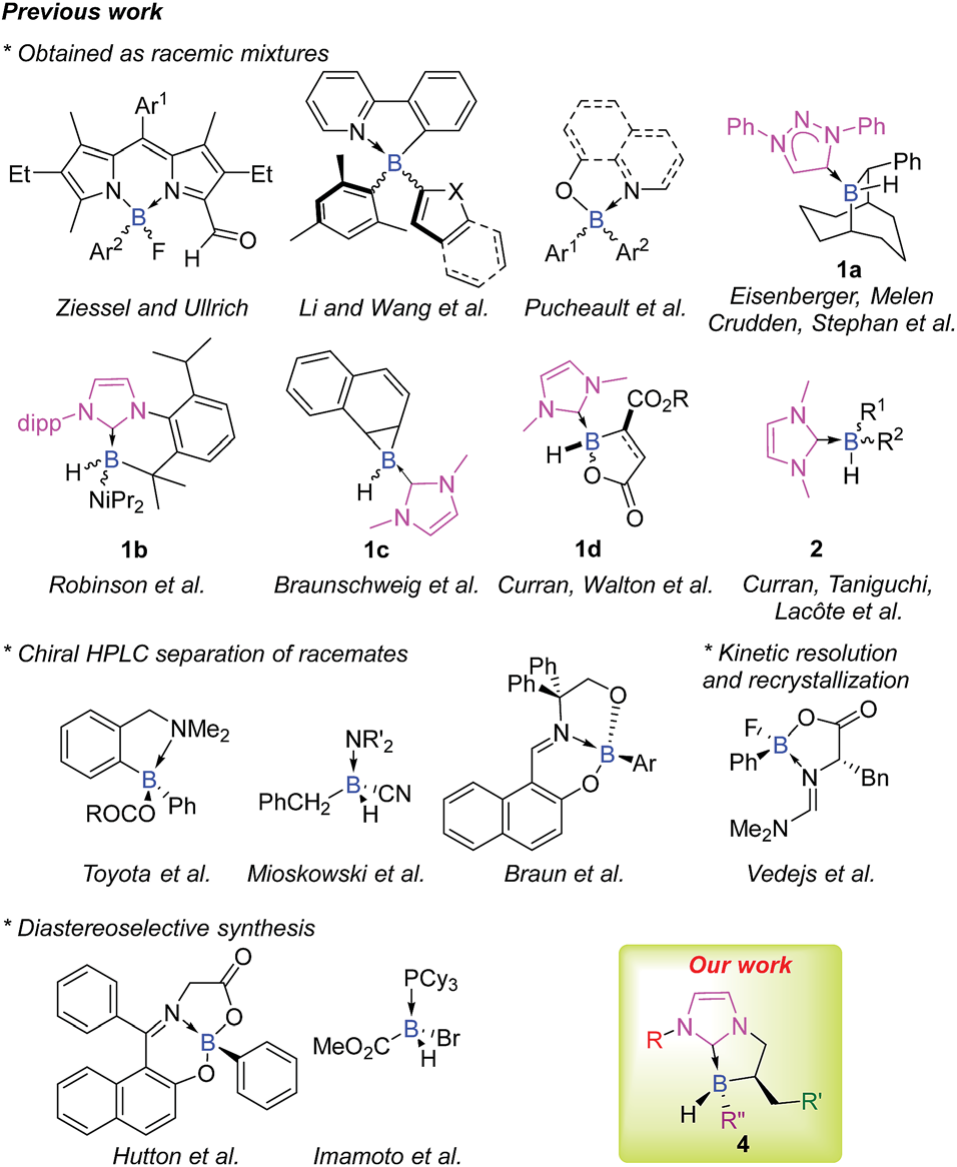
Representative examples of chiral platforms stereogenic at boron and the strategy of preparation.

Stereogenic main group elements other than carbon have gained interest, and a great deal of work has been done to obtain stereogenic S,^5^ Si,^6^ P^7^ or N atoms.^8^ Astonishingly, while chiral organoboron reagents are very useful in stereoselective transformations, efforts are still needed regarding the design of scaffolds stereogenic at boron, and only a handful of configurationally stable ones have been reported to date, mainly as racemates (Fig. 1).^9^ Expanding the chemical space of stereogenic tetrahedral-coordinated boron is of paramount importance and should open interesting new prospects especially in the field of metal-free catalysis where tetravalent boron structures are involved as key intermediates in borane or, more recently, in borenium catalysis.^10^ This area is still in its infancy and the design and preparation of chiral platforms stereogenic at boron are challenging tasks. This is mostly due to the labile nature of ligands attached to the tetracoordinated boron atom that makes it configurationally unstable. Accordingly, the choice of the nature of boron's ligands is crucial.

In this context, N-heterocyclic carbene boranes (NHC-boranes) have been the subject of significant research. Efforts have been devoted to gain better insights into their physicochemical properties and reactivity profiles^11^ and rapidly, their scope of application in organic synthesis (radical,^12^ ionic,^13^ and organometallic reactions^14^) has showcased their interesting added value. The recent infatuation for NHC ligated boranes also comes from the fact that they are easily available, stable and, more importantly, are quite resistant to dissociation.^11^ Nevertheless, only a few scaffolds of cyclic **1a–d**^15^ and acyclic **2** (ref. 12*d* and 16) NHC-boranes stereogenic at the boron atom in the racemic form have previously been reported (Fig. 1).

Herein, we report the controlled diastereoselective preparation of stable chiral NHC-borane **4** stereogenic at boron (up to 99 : 1 dr) (Fig. 1) *via* boron–carbon bond formation starting from commercially available Grignard reagents and our in-house cyclic NHC-boranes **3** synthesized *via* an enantioselective rhodium-mediated boracyclopentannulation reaction.^17^ Thanks to this tunable platform a delicate balance between steric and electronic factors could be controlled directly at the boron atom.

## Results and discussion

### Preliminary results

**Scheme 1:**
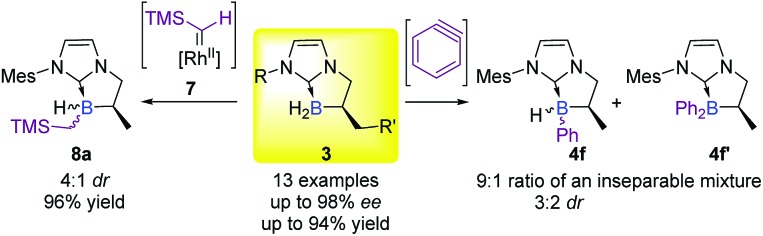
Diastereoselective one-step functionalization of chiral NHC-boranes.

Based on the literature precedents and starting from our tailor-made cyclic NHC-boranes **3**, several methods were at our disposal for the formation of a new boron–carbon bond. However, despite all our efforts and the optimized procedure from the literature, the direct hydroboration of arynes with NHC-boranes16*a* provided an inseparable mixture of mono-**4f** and bis-phenylborane **4f′** products (up to a 9 : 1 ratio) and poor diastereoselectivity for **4f** (up to 3 : 2 dr)‡
‡The diastereomeric ratio was determined for the crude by ^1^H NMR (in CD_2_Cl_2_), and for the diastereotopic CH_2_ protons of the boracyclic moiety. (Scheme 1).† On the other hand, insertion of the rhodium carbene **7** into the B–H bond pleasingly delivered the desired product **8a** in a good yield (96%),16*b* albeit with modest diastereoselectivity (up to 4 : 1 dr)‡ and with a limited substrate scope.† Consequently, a more general approach, starting from easily and commercially available substrates was highly necessary.

**Scheme 2:**
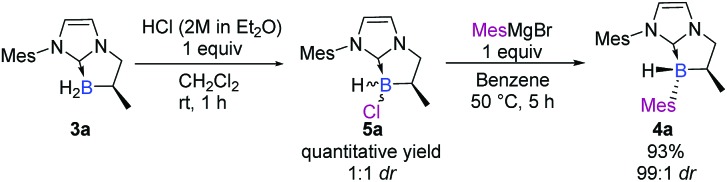
Diastereoselective two-step preparation of chiral NHC-boranes.

We turned our attention to a two-step diastereoselective chlorination/arylation procedure (Scheme 2).^18^ The cyclic chiral NHC-borane **3a** simply treated with an equivalent of hydrogen chloride yielded the chlorinated NHC-borane **5a** in a quantitative yield albeit in a 1 : 1 ratio of diastereomers.‡ Then, **5a** was treated with an equivalent of the mesityl Grignard reagent at 50 °C for 5 hours in benzene. To our delight, the desired adduct **4a** was obtained not only in an excellent 93% yield but also as a single diastereomer‡ (Scheme 2). The structure and the absolute stereochemistry of **4a** were obtained by X-ray diffraction analysis, thus confirming the anticipated *trans* relationship between the B-mesityl group and the adjacent methyl substituent.^19^

### Scope and limits of the reaction

**Scheme 3:**
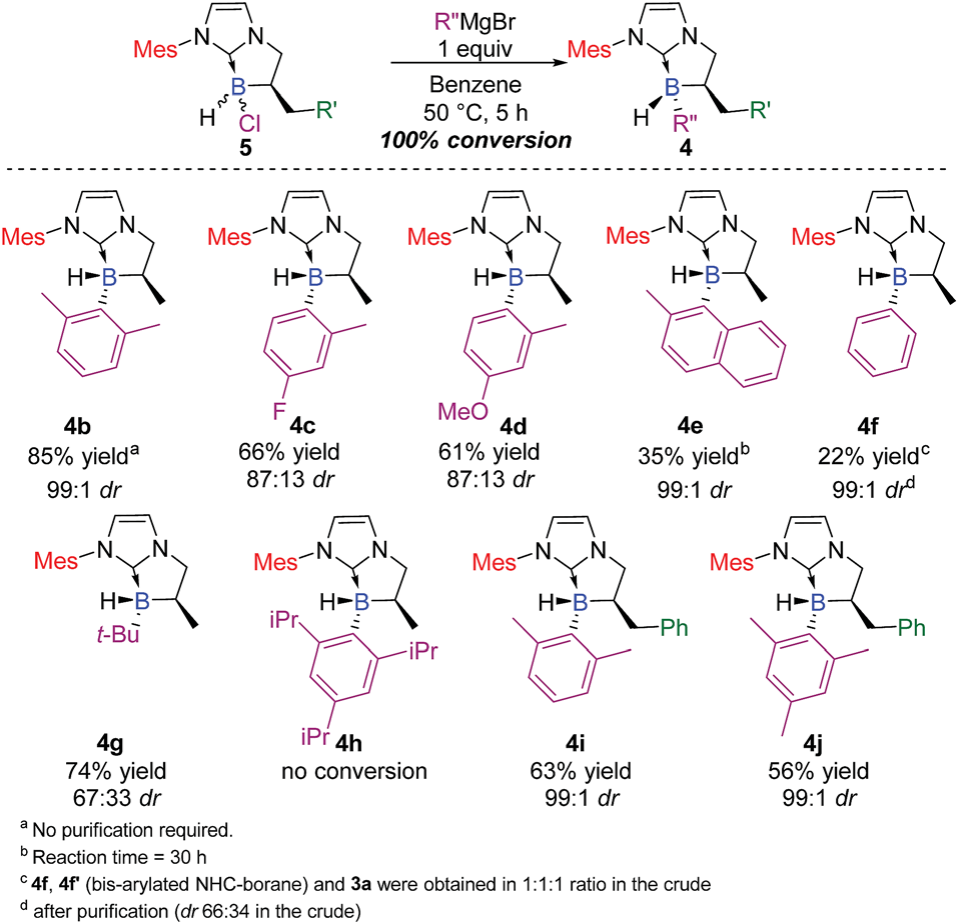
Scope of the diastereoselective functionalization of NHC-borane **5** with various Grignard reagents (ESI†).

Triggered by this impressive and unprecedented result, we next examined the scope and limits of the reaction. Application of these conditions across a variety of Grignard reagents was then undertaken. The reaction proceeded smoothly in most cases with total conversion,^20^ with yields up to 85% and diastereoselective ratios‡ ranging from 67 : 33 to 99 : 1 (Scheme 3). Compounds bearing electron-withdrawing **4c** (66% yield, 87 : 13 dr, *trans* as the major diastereomer) or electron-donating groups **4d**^21^ (61% yield, 87 : 13 dr, *trans* as the major diastereomer) showed similar reactivity, indicating that the electronic nature of the Grignard substrate does not play an essential role in the transformation. The reaction proved to be compatible with an aliphatic Grignard reagent and delivered the desired adduct **4g** in a good yield (74%), albeit with lower diastereoselectivity (67 : 33 dr). As a further demonstration of the generality of the process, 2,6-methylphenyl and 2-methyl-1-naphthyl Grignard reagents were also successfully used in the reaction and yielded the corresponding NHC-boranes **4b**^22^ and **4e** in 85 and 35% yields, respectively (99 : 1 dr, *trans* as the major diastereomer).

Limits were reached with simple phenyl magnesium bromide which gave a mixture of mono-(**4f**) and bis-phenyl-functionalized (**4f′**) NHC-boranes, and surprisingly, with the concomitant formation of **3a** (1 : 1 : 1 ratio in the crude, see below in the Mechanism section for a plausible explanation and in the ESI for complementary experiments, pages S75 and S76†). On the other hand, no reaction could take place with a much hindered organomagnesium reagent (see 4 h). Finally, the wide scope of the process was evaluated with respect to the α-benzyl-substituted boron atom. The reaction yielded the corresponding trisubstituted NHC-boranes **4i–j** in good yields (63 and 56% respectively) and excellent diastereoselectivities (99 : 1 dr).

**Scheme 4:**
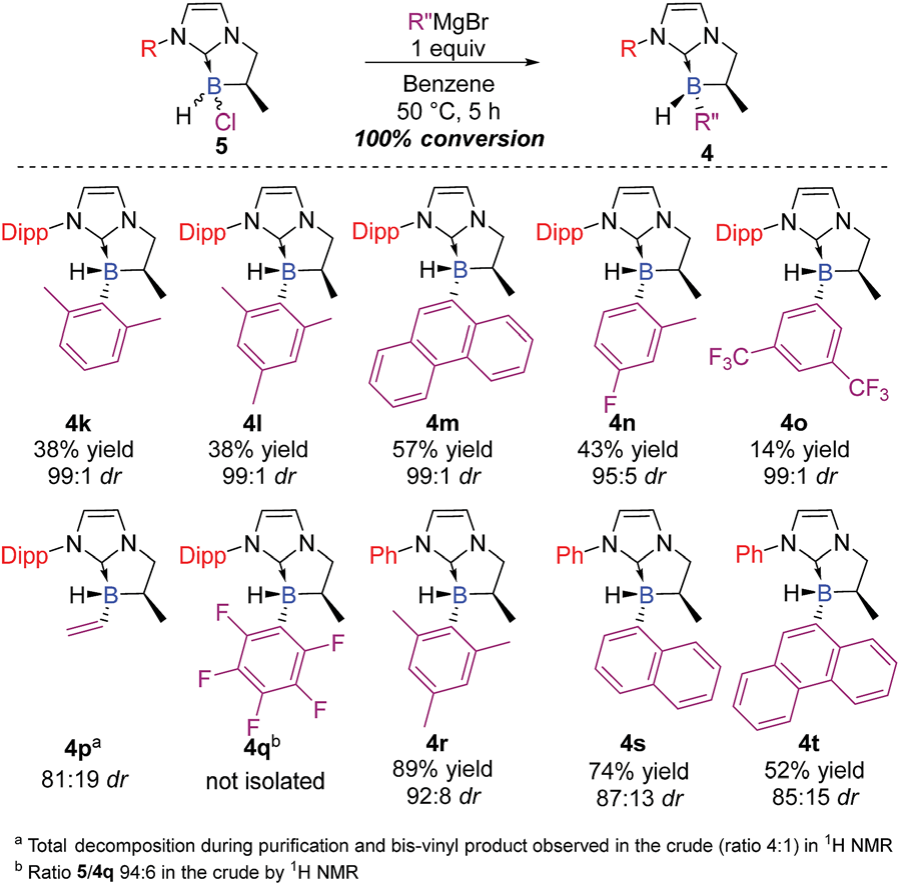
Scope of the diastereoselective functionalization of NHC-boranes with various N-substituents on the starting NHC-borane scaffold **5** (ESI†).

Attention was then turned to the N-substituted NHC-backbone. As shown in Scheme 4, the reaction was compatible with Dipp (2,6-diisopropylphenyl) (**4k–o**) or Ph (**4r–t**) N-substituents providing a library of novel chiral NHC-boranes. Unfortunately, NHC-borane **5** did not react with the perfluorinated phenyl Grignard reagent, and a ratio of only 94 : 6 of the starting material to **4q** was observed in the crude by ^1^H NMR. On the other hand, total conversion of the starting material and good diastereoselectivity (81 : 19 dr) was observed in the case of the vinyl derivative **4p**; however, the latter decomposed during purification, but the yield was estimated to be 45% in the crude by ^1^H NMR after filtration on a short pad of Celite©.

**Scheme 5:**
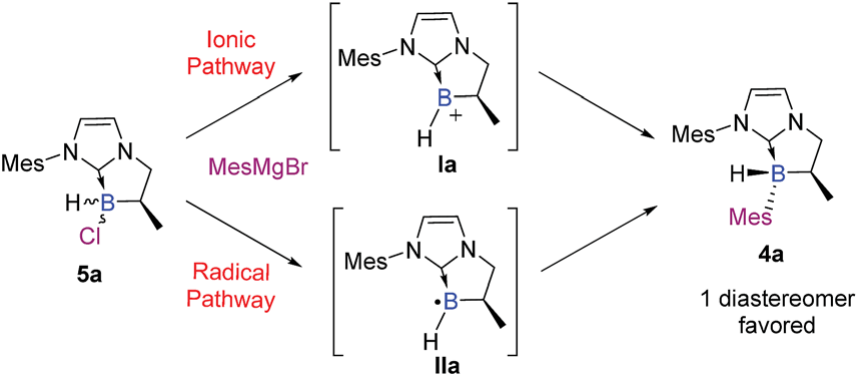
Suggested pathways for the diastereoselective functionalization of NHC-boranes.

To summarize, this two-step sequence led to the highly diastereoselective formation of novel NHC-boranes **4**, stereogenic at boron, in good yields and with high diastereoselectivities, driven notably by bulky substituents on the nitrogen atom of the NHC-core (*i.e.* Mes or Dipp). Moreover, the excellent diastereoselectivity observed in the last step drew our attention and naturally led us to an understanding of the reaction mechanism. Obviously, the S_N_2 pathway was immediately ruled out. Therefore, two plausible mechanisms *via* a trivalent boron species remained: an ionic (S_N_1) pathway or a radical (S_RN_1) one (Scheme 5).

### Insights into the mechanism

**Scheme 6:**
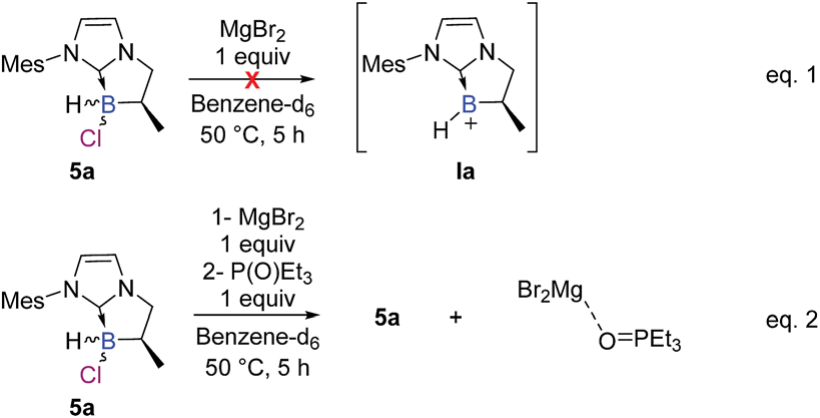
Control experiments of the hypothetical S_N_1 mechanism *via* the borenium cation **Ia**.

To further elucidate the mechanism, we decided to first examine the ionic route. We hypothesized that an intermediate borenium trivalent species **Ia** could result from chloride abstraction by a magnesium Lewis acid. Grignard reagents in solution are particularly complex since multiple species are present at thermodynamic equilibrium. More precisely, *via* the Schlenk equilibrium, magnesium bromide (MgBr_2_),^23^ a weak halophile, could promote the formation of the borenium derivative at thermodynamic equilibrium. However, when borane **5a** was treated with MgBr_2_ (1 equiv) in benzene-*d*_6_, the intermediate borenium **Ia** was not observed by ^11^B NMR (Scheme 6, eqn (1)). Needless to say, the non-observation of **Ia** by ^11^B NMR is not evidence to rule out the formation of the intermediate borenium and excludes a plausible ionic pathway. Accordingly, the experiment with MgBr_2_ was next repeated in the presence of a borenium trap [P(O)Et_3_] to displace the potential thermodynamic equilibrium (Scheme 6, eqn (2)). However, the sole product observed was the MgBr_2_·P(O)Et_3_ complex (^31^P NMR; *δ* = 64.4 ppm in benzene-*d*_6_) and no trace of the expected boronium phosphine oxide complex was found. Based on these new results, the ionic pathway seems to be ineffective and additional studies were then undertaken towards the radical pathway.

**Scheme 7:**
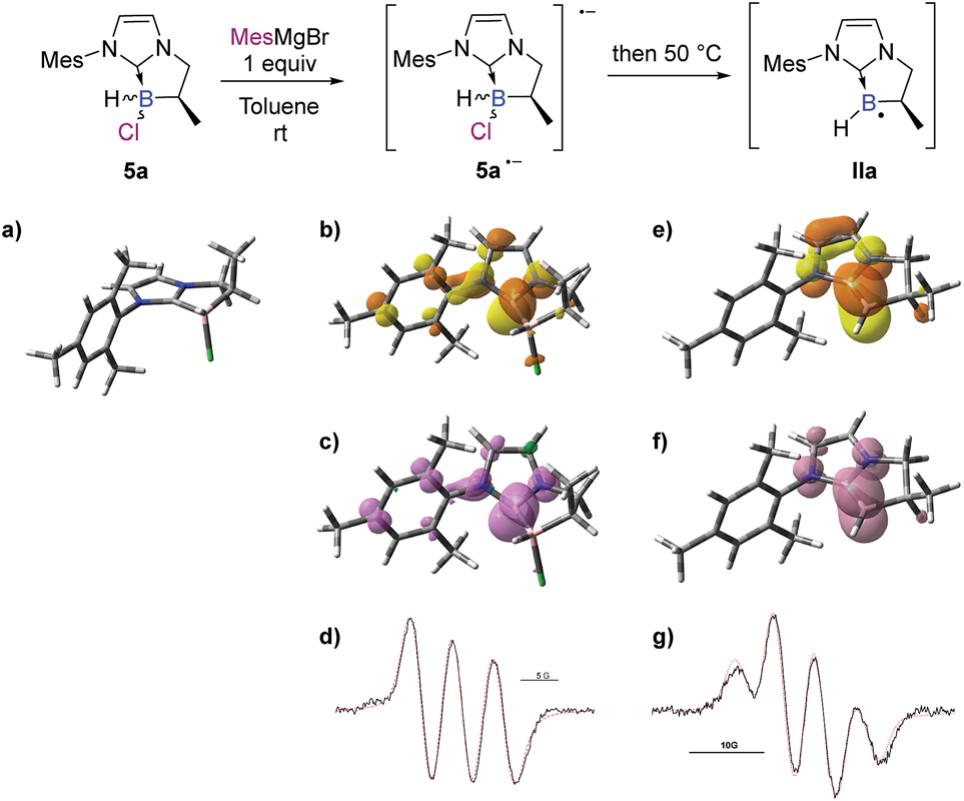
EPR spectra and DFT analysis (uM06-2X/6-311G+(d,p) level of theory) of **5a**, **5a˙^–^** and **IIa**. (a) Optimized structure of *trans*-**5a**. Selected bond distances (Å): B–C_NHC_ 1.62; B–Cl 1.91. Contour plots (b) of the SOMO (isovalue = 0.05) and (c) of the electron density (isovalue = 0.004) of **5a˙^–^**. Selected bond distances (Å): B–C_NHC_ 1.59; B–Cl 2.01. (d) Experimental (black) X-band EPR spectrum of **5a˙^–^** recorded at 25 °C in toluene and its superimposed simulation (red), *a*_N1_ = 4.7 G, *a*_N2_ = 0.8 G, and *a*_B_ = 0.5 G. Contour plots (e) of the SOMO (isovalue = 0.05, NBO charges: B(0.219); C_NHC_(0.114)) and (f) of the electron density (isovalue = 0.004) of **IIa**. Selected bond distance (Å): B–C_NHC_ 1.50. (g) Experimental (black) X-band EPR spectrum of **IIa** recorded at 50 °C in toluene and its superimposed simulation (red), *a*_B_ = 4.5 G, *a*_H_ = 0.7 G and *a*_H_ = 1.6 G.

A control EPR experiment was performed. A solution of NHC-borane **5a** and the mesityl Grignard reagent in deoxygenated toluene was placed in the cavity of a CW-X-band EPR spectrometer. Before heating the medium, a spectrum showing three main lines of equal intensity, broadened by several weak hyperfine couplings, was systematically recorded (Scheme 7d).

Considering that Grignard reagents are subject to one-electron oxidation through a SET mechanism,^24^,^25^ this spectrum could reasonably be assigned to the radical anion species of **5a** (**5a˙^–^**), as routinely described for the initiation step in the chain mechanism of the S_RN_1 reaction.^26^ Its computer simulation yielded the following hyperfine coupling constant (hfcc) values: *a*_N1_ = 4.7 G, *a*_N2_ = 0.8 G, and *a*_B_ = 0.5 G, which are consistent with major localization of the unpaired electron on the carbenic atom of the NHC.

To rationalize these EPR observations, calculations were performed on the radical anion **5a˙^–^** in the gas phase by means of DFT methods at the uM06-2X/6-311G+(d,p) level of theory (see the ESI† for details). The shape of the calculated singly occupied molecular orbital (SOMO) shows considerable localization of the unpaired electron on the carbenic atom of the NHC (Scheme 7b and c), as revealed in the EPR study. Moreover, the optimized geometry of the radical anion **5a˙^–^** exhibits an elongated B–Cl bond length (201.1 pm) compared to that of the chlorinated NHC borane **5a** (191.4 pm), which helps to predict the easy fragmentation of the B–Cl bond at 50 °C to generate the boryl radical intermediate **IIa**.

The medium was then heated to 50 °C directly in the spectrometer cavity and the EPR signal shown in Scheme 7g was recorded. Its analysis revealed the presence of a second radical species showing a hfcc with the boron isotope ^11^B in mixture with the one observed at room temperature. After simulation, the following hyperfine coupling constants were determined for this second radical: *a*_B_ = 4.5 G, *a*_H_ = 0.7 G and *a*_H_ = 1.6 G. These results suggest considerable localization of the unpaired electron on the boron atom of **IIa** and are consistent with the literature precedent.^27^ The resulting half-life of the putative radical **IIa** is *ca.* 2.5 min (at 50 °C), revealing its relative persistence under our experimental conditions. Calculations were also performed on the boryl radical **IIa** (DFT methods at the uM06-2X/6-311G+(d,p) level of theory, see the ESI† for details). Firstly, the optimized geometry of the NHC boryl radical **IIa** exhibits a shorter B–C_NHC_ bond length (149.6 pm) compared to that of the NHC-borane **5a** (162.0 pm). The boryl radical is planar at the boron atom and the radical is partially delocalized into the π-system of the NHC. The boryl radical character of **IIa** is further supported by the shape of the calculated singly occupied molecular orbital (SOMO) and the respective contributions of the carbenic atom of NHC and the boron atom (B/C_NHC_ ratio of the spin density in **IIa** is about 8.1 : 1.9 by NBO analysis),† fitting perfectly with the EPR observations (Scheme 7e and f).

**Scheme 8:**
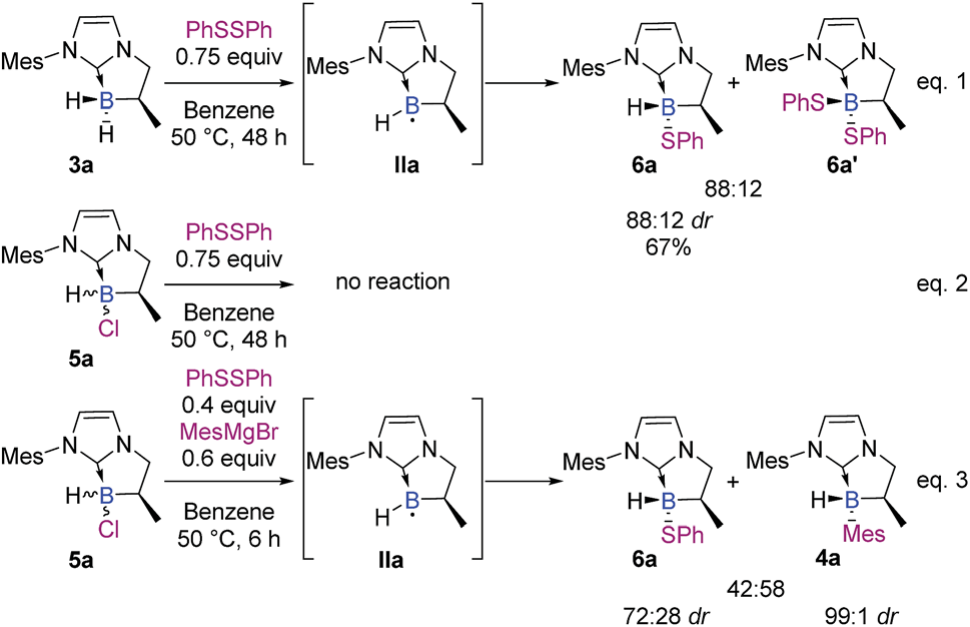
Reactivity of disulfide reagents with NHC-boranes **3a** and **5a**, and cross-reactivity with the Grignard reagent.

Unfortunately, classical attempts to experimentally trap the putative radical **IIa**, in the presence of the Grignard reagent, did not reach our expectations (spin traps used: PBN, MNP, DMPO, and TEMPO). On the other hand, the boryl radical **IIa** was not EPR-observed after treatment of **5a** in toluene at 25 °C or 50 °C with lithium or magnesium metals or a sodium/mercury metal amalgam. Based on some observations of the reactivity of our NHC-boranes with disulfide reagents, we managed to indirectly trap **IIa** and ascertain its reactivity (Scheme 8). In the presence of diphenyl disulfide, NHC-borane **3a** gave the corresponding phenylsulfide NHC-borane adduct **6a** in a 67% yield (88 : 12 dr),‡ together with the disubstituted adduct **6a′** (88 : 12 ratio)‡ (Scheme 8, eqn (1)) through a postulated radical mechanism.^28^ Under the same conditions, NHC-borane **5a** was found to be inert (Scheme 8, eqn (2)).

**Scheme 9:**
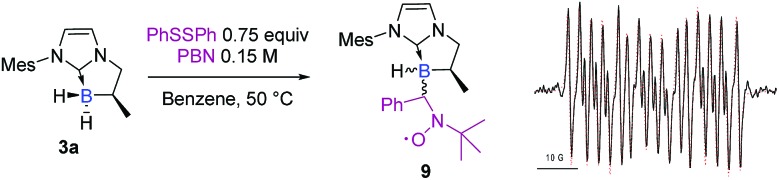
Experimental (black) X-band EPR spectrum of **9** recorded at 50 °C in toluene and its superimposed simulation (red), *a*_N_ = 14.7 G, *a*_H_ = 3.9 G, and *a*_B_ = 2.9 G.

A control spin trapping experiment was then performed with **3a** in the presence of phenyldisulfide and α-phenyl-*N*-tertiary-butyl nitrone (PBN) as the spin trap (Scheme 9); an intense spin adduct signal was recorded. Its computer simulation allowed us to assign this signal to the boron-centered radical adduct of PBN (*a*_N_ = 14.7 G, *a*_B_ = 2.9 G and *a*_H_ = 3.9 G), *i.e.* the nitroxide radical **9**. This undoubtedly confirms the radical pathway for the formation of **6a**. Interestingly, a mixture of NHC-borane **5a** (1 equiv), phenyldisulfide (0.4 equiv) and MesMgBr (0.6 equiv) in benzene at 50 °C led to the NHC-boranes **4a** and **6a** and in a 3 : 2 ratio as the sole products in the crude by ^1^H and ^11^B NMR,† thus demonstrating that the boron centered radical **IIa** putatively arises from the reduction of the chlorinated NHC borane **5a** by the Grignard reagent and then reacts with phenyldisulfide (Scheme 8, eqn (3)).

**Scheme 10:**
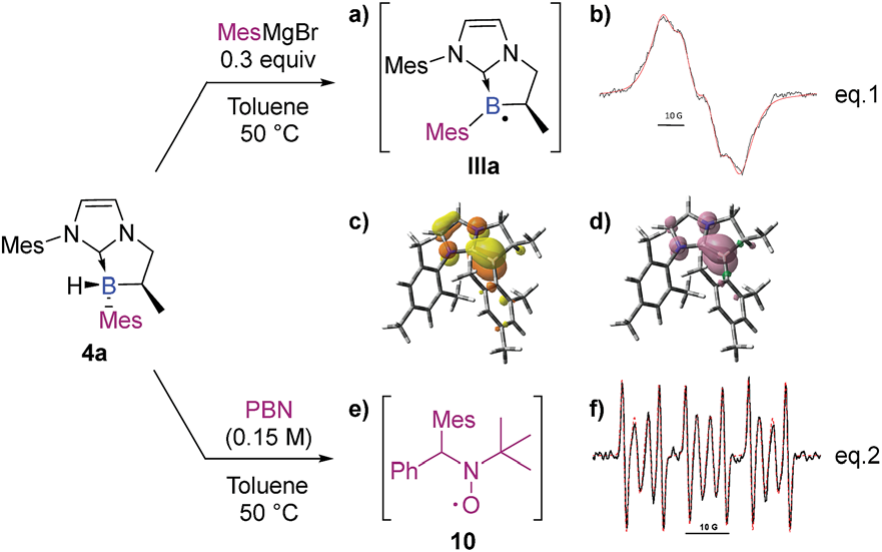
EPR spectra and DFT analysis (uM06-2X/6-311G+(d,p) level of theory) of **IIIa** and **10**. (a) NHC-boryl radical **IIIa**. (b) Experimental (black) X-band EPR spectrum of **IIIa** recorded at 50 °C in toluene and its superimposed simulation (red), *a*_H_ = 4.2 G, *a*_N_ = 2.0 G (*2N*), and *a*_B_ = 6.4 G (^11^B–80%) and *a*_B_ = 2.1 G (^10^B–20%). Contour plots (c) of the SOMO (isovalue = 0.05, NBO charges: B(0.521); C_NHC_(0.093)) and (d) of the electron density (isovalue = 0.004) of **IIIa**. Selected bond distances (Å): B–C_NHC_ 150.9; B–C_Mes_ 157.2. (e) Mesityl/PBN radical adduct **10**. (f) Experimental (black) X-band EPR spectrum obtained by heating **4a** in benzene at 50 °C in the presence of PBN, and its superimposed simulation (red); the major signal corresponds to the Mes/PBN adduct **10** (*a*_N_ = 14.6 G, *a*_H_ = 8.5 G, 60%).

Finally, and still from the viewpoint of rationalizing the formation of the by-products (**3** and **4′**) *via* a radical pathway, the stability of the final product **4a** was analyzed under the reaction conditions. It turned out that a radical species formed from MesMgBr was able to abstract the remaining hydrogen atom (B–H). Accordingly, a medium containing NHC-borane **4a** (1.0 equiv) and MesMgBr (0.3 equiv) was prepared in toluene and submitted to EPR analysis. If no signal was observed before heating, raising the temperature to 50 °C yielded an intense and wide signal (Scheme 10, eqn (1)). Its simulation (superimposed red dotted lines) was achieved by considering a single paramagnetic species with hyperfine couplings with a boron, a hydrogen and two nitrogen nuclei, and led to the following hyperfine splitting constants: *a*_H_ = 4.2 G, *a*_N_ = 2.0 G (*2N*), and *a*_B_ = 6.4 G (^11^B–80%) or *a*_B_ = 2.1 G (^10^B–20%) (Scheme 10b). The value obtained for a_H_ is consistent with a hydrogen nucleus in the α-position towards the radical center. It should also be noted that the values obtained for *a*_B_^11^ and *a*_B_^10^ perfectly fit with the gyromagnetic ratio of these two isotopic nuclei: *γ*_B_^11^/γ_B_^10^ = 2.99 and *a*_B_^11^/*a*_B_^10^ = 3.05. According to this experiment, the spectrum was assigned to a boron-centered radical **IIIa** formed after the reaction with MesMgBr as a reductant (Scheme 10a). Calculations were also performed on the boryl radical **IIIa** (DFT methods at the uM06-2X/6-311G+(d,p) level of theory).† As in the boryl radical **IIa**, **IIIa** presents a shorter B–C_NHC_ bond length (150.9 pm) compared to the NHC-borane **5a** (162.0 pm). **IIIa** is planar at the boron atom and the radical is partially delocalized into the π-system of the NHC (B/C_NHC_ ratio of the spin density in **IIIa** is about 8.0 : 2.0 by NBO analysis in the SOMO).† These results clearly show that Grignard reagents can abstract hydrogen atoms by homolytic cleavage, therefore explaining the formation of the subsequent disubstituted NHC-boranes with less hindered Grignard reagents (as observed with phenyl and vinyl Grignard, see **4f** and **4p**).

Interestingly, despite the fact that borane **4a** in toluene was found to be EPR silent, additional spin-trapping experiments performed at 50 °C on **4a** in the presence of the spin trap α-phenyl-*N*-tertiary-butylnitrone (PBN) clearly gave a 6 line EPR spectrum as shown in Scheme 10f (*a*_N_ = 14.6 G and *a*_H_ = 8.5 G), which could be assigned to the mesityl/PBN radical adduct **10** (Scheme 10e, eqn (2)), as previously described by Yoshida *et al.*,^29^ mixed with a minor unidentified carbon-centered radical (*a*_N_ = 14.5 G, *a*_H_ = 3.2 G, 40%, signal also observed before heating, see the ESI†). This last experiment clearly demonstrates the possible existence of an equilibrium between the radical species **IIa** and the formation of **4a** through homolytic cleavage of the B–C_Ar_ bond. Consequently, hydrogen atom transfer may arise from any species able to produce hydrogen radicals. To acquire thermodynamic information, DFT calculations [M06-2X/6-311 g(d,p)] were performed on *trans* and *cis* isomers of **4a** and **4d**. Energy differences of 2.99 and 1.63 kcal mol^–1^ were, respectively, found in favor of the *trans*-diastereomer which is in full agreement with the experimental diastereomeric ratios obtained.†

Accordingly, based on these experimental observations and theoretical investigations, all these data suggest a probable S_RN_1-type mechanism initiated by a SET from the Grignard reagent to chlorinated NHC-borane **5a**. The radical anion **5a˙^–^** thus generated reacts upon fragmentation at 50 °C and provides the corresponding boryl radical **IIa** along with the chloride ion. Then, **IIa** reacts with the Grignard reagent in the chain mechanism as classically described in the S_RN_1 mechanism.^26^

### Perspective

**Scheme 11:**
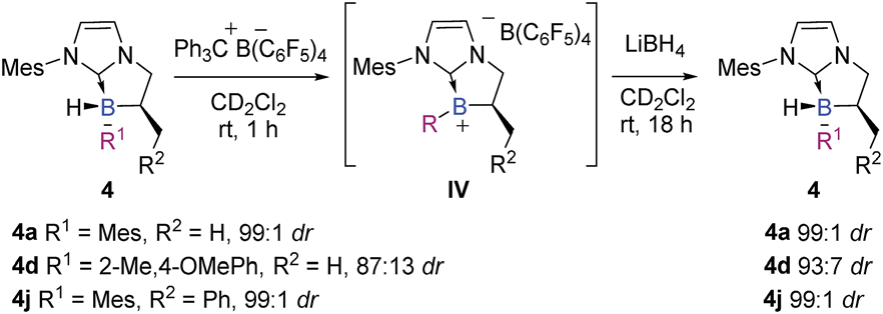
Highly diastereoselective addition of hydride on the borenium **IV** intermediate to recover chiral NHC-borane **4**.

**Scheme 12:**
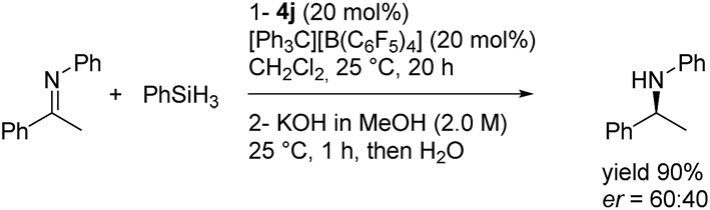
Imine reduction with silane catalyzed by chiral NHC-borenium.

In the view point of using chiral platform **4** as a chiral hydride source in reduction reactions, the diastereoselective regeneration of the NHC-borane from the corresponding planar NHC-borenium is the key step in a hypothetical catalytic cycle, by analogy with the concept developed by Seebach on carbon stereocenters^30^ and transposed here to the boron atom. Accordingly, after the smooth and quantitative formation of the intermediate trivalent borenium **IVa** (^11^B NMR *δ* = 71.7 ppm, CD_2_Cl_2_), from **4a** in the presence of trityl tetrakis(pentafluorophenyl) borate in deuterated dichloromethane, the borocation was treated with lithium borohydride as a quantitative hydride donor.§
§Silanes or boranes were also used as hydride donors in FLP systems, but as depicted in the literature the hydride is, at best, distributed between the borenium and the hydride source in the absence of an unsaturated acceptor. In these cases, the borane is not regenerated in the presence of a silane source and in the absence of a Lewis base (see ESI p. S82–S85 for spectra with PhSiH_3_ and Et_3_SiH as hydride sources). To our delight, the hydride addition to borenium IVa fully regenerated the original diastereomer of NHC-borane **4a** with a total retention of the configuration at the boron atom as indicated by ^1^H NMR for the crude. Moreover, starting from a less diastereo-enriched NHC-borane **4d** (87 : 13 dr), the reaction of the borenium intermediate **IVd** (^11^B NMR *δ* = 64.1 ppm, CD_2_Cl_2_) with LiBH_4_ regenerates **4d** with improved diastereoselectivity (93 : 7 dr) compared to that of the starting material (Scheme 11), showing that hydride addition on this borenium platform is highly diastereoselective. These experiments nicely illustrate the high potential of the designed species **4**. This original chiral environment imparted by the stereogenic boron atom and assisted by the α-carbon stereocenter could be groundbreaking in the field of borenium-catalyzed reduction reactions, for example, where the stereo defining step is the hydride delivery one. To give more substance to the concept, the synthetic utility of these B-stereogenic NHC-boranes was checked in the borenium-catalyzed hydrosilylation of (*E*)-*N*,1-diphenylethan-1-imine with phenylsilane. The borenium was generated *in situ* from NHC-borane **4j** with trityl tetrakis(pentafluorophenyl)borate. The catalytic process gave the corresponding amine in a 90% yield and a 60 : 40 enantiomeric ratio (Scheme 12). Despite the modest enantioselectivity, this is the best example of enantioselective hydrosilylation of an imine catalyzed by a chiral NHC-borenium.

## Conclusions

In conclusion, the diastereoselective synthesis of a series of new stable and enantioenriched NHC-borane complexes stereogenic at boron has been reported. Our results demonstrate that single diastereomers can be obtained in a high yield starting with a 1 : 1 diastereomeric mixture of chlorinated NHC-borane **5**, representing a rare example of stereoselective synthesis of a chiral scaffold stereogenic at the boron atom.^31^ Preliminary results regarding the mechanistic studies indicate that a radical pathway (S_RN_1) is more likely to occur. Another important finding from this study is the stereoselective regeneration of the boron stereogenic center, from a borenium intermediate with a hydride, with excellent diastereoselectivity, therefore providing a promising strategy for metal-free enantioselective reduction catalysis.15*a* Future studies should explore the catalytic activity of this unprecedented scaffold, and more specifically, the scope and the selectivity of the borenium-catalyzed reactions, particularly FLP-type reduction reactions.

## Conflicts of interest

There are no conflicts to declare.

## Supplementary Material

Supplementary information

Crystal structure data
