# Supplementary material for: Highly diastereoselective preparation of chiral NHC-boranes stereogenic at the boron atom
Source: Chem Sci. 2019 May 24;10(26):6524–30. doi: 10.1039/c9sc01454c (PMC6611065; doi:10.1039/c9sc01454c)
Supplement: Supplementary file 1 [file SC-010-C9SC01454C-s001.pdf]

Electronic Supplementary Material (ESI) for Chemical Science.
This journal is © The Royal Society of Chemistry 2019

Highly diastereoselective preparation of chiral NHC-boranes stereogenic at boron atom.

Clara Aupic,[a] Amel Abdou Mohamed,[a] Carlotta Figliola,[a] Paola Nava,[a] Béatrice Tuccio,[b] Gaëlle Chouraqui,[a] Jean-Luc Parrain,[a] Olivier Chuzel*[a]

[a]Aix Marseille Univ, CNRS, Centrale Marseille, iSm2, Marseille, France
[b]Aix Marseille Univ, CNRS, ICR, Marseille, France

Supporting Information

1. General Experimental **2**
2. Preliminary attempts to form the B-C bond **3**
a. Hydroboration of aryne 3
b. B-H bond-rhodium carbene insertion 4
3. Procedure and Analytical Data **6**
a. Synthetic procedures and spectroscopic data for the preparation of chlorinated NHC-borane **5** 6
b. Synthetic procedures and spectroscopic data for the preparation of NHC-borane **4** 10
4. NMR Spectra **28**
5. Modeling approach for *trans*-5a, *cis*- & *trans*-5a•-, IIa and IIIa **61**
a. *trans*-NHC-borane **5a** 61
b. NHC-borane radical anion **5a•-** 62
c. NHC-boryl radical **IIa** 65
d. NHC-boryl radical **IIIa** 66
6. Origin of the diastereoselectivity in the formation of NHC-boranes 4a and 4d **69**
a. DFT calculations 69
b. EPR Studies 75
7. Mechanism studies **76**
a. Ionic pathway 76
b. Radical pathway 78
8. Catalysis **81**
a. Imine reduction with silane catalyzed by chiral NHC-borenium 81
b. Borenium **IVa**-Silane complex studies 82
9. Cristal structures **86**

1. General Experimental

All reactions were performed using standard Schlenk techniques under inert atmosphere of dry argon or in a glovebox Jacomex GP (concept)-II-P. ^1H NMR spectra were measured on a Bruker AC 400 (400 MHz) or a Bruker AC 300 (300 MHz) spectrometer at 298 K (20 °C). Data were reported as follows: chemical shifts in ppm referenced to the internal solvent signal (peak at 7.26 ppm in the case of $CDCl_3$; peak at 5.32 in the case of CD_2Cl_2), multiplicity (s = singlet, d = doublet, t = triplet, q = quartet, quint = quintet, sex = sextet, sep = septet, dd = doublet of doublets, m = multiplet, br = broad signal) and coupling constants (Hz). ^{13}C {^1H} NMR spectra were measured on a Bruker AC 400 (100 MHz) or a Bruker AC 300 (75 MHz) spectrometer with complete proton decoupling. Chemical shifts were reported in ppm from the internal solvent signal (peak at 77.16 ppm in the case of $CDCl_3$, peak at 53.84 in the case of CD_2Cl_2). The resonance of the carbene carbon and any tetrasubstituted carbon α to the boron center could not be observed. ^{11}B NMR spectra were measured on a Bruker AC 400 (128 MHz). ^{11}B chemical shifts are given relative to boron trifluoroetherate (^{11}B = 0 ppm) used as external reference. High-resolution mass spectra (HRMS) were performed on a SYNAPT G2 HDMS (Waters) spectrometer equipped with atmospheric pression ionization source (API) pneumatically assisted. Samples were ionized by positive electrospray mode as follows: electrospray tension (ISV): 2800 V; opening tension (OR): 20 V; nebulization gas pression (nitrogen): 100 L/h. Infrared spectra were recorded on a Bruker TENSOR 27 Fourier Transform infrared spectrometer equipped with a single reflection diamond Attenuated Total Reflexion accessory (Bruker A222). The measurements were done for pure samples at room temperature. For each individual spectrum, about 20 scans were average at 4 cm^{-1} resolution. The Fourier Transform (FT) of the interferograms have been done using the Blackaman-Harris 3-term apodization function, the Mertz phase correction mode and zerofilling factor of 2. The diamond crystal without sample served as reference. Wavenumbers are reported in cm^{-1} with the corresponding intensities s (strong), m (medium), w (weak). The X-band EPR spectra were recorded on a Bruker EMX spectrometer with a continuous flow at 298 K. Unsaturating microwave power: 10-20 mW, modulation range: 0.1-1.5 G; scanning time range: 10-42 s; receptor gain: 5.10^4 to 10^6; number of scans: from 1 to 9. The probes were transferred into a sealed and deoxygenated NMR tube and the spectra were simulated with WinSim. Analytical thin layer chromatography (TLCs) were developed on silica Macherey-Nagel 818333 plates. Visualization was achieved under a UVP mineralight UVGL-58 lamp, and by developing the plates with vanillin reagent. The products were purified by flash column chromatography on silica gel 60 (Macherey-Nagel® Si 60, 0.040-0.063 mm). Anhydrous dichloromethane, toluene and tetrahydrofuran were obtained from the Solvent Purification System BRAUN MB-SPS800. Benzene was purchased anhydrous over 4 Å molecular sieves and stored in the glovebox. Unless otherwise noted, other analytical grade solvents were used without further purification. All reagents were obtained from commercial suppliers unless otherwise stated.

2. Preliminary attempts to form the B-C bond

a. Hydroboration of aryne

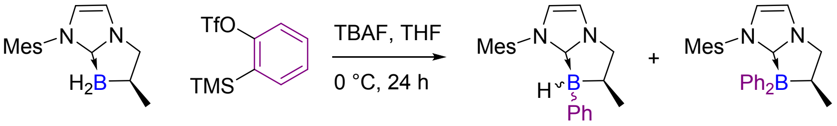

3a **4f** **4f'**

To a stirred solution of 2-(trimethylsilyl)phenyl trifluoromethanesulfonate (0.35 mmol, 2.0 equiv) and NHC-borane **3a** (0.17 mmol, 1.0 equiv) in THF (2.0 mL) at 0 °C, was added *n*-tetrabutylammonium fluoride (1.0 M in THF, 2.4 equiv, 0.42 mL). The mixture was stirred at 0 °C for 24 h. Then the reaction mixture was filtered through a short plug of silica gel using dichloromethane as eluent, then the solvent was evaporated under reduced pressure. The crude material was purified by silica gel chromatography using pentane:diethylether 95:5 as eluent to give the borane **4f** in mixture with the bisfunctionalized product **4f'**.

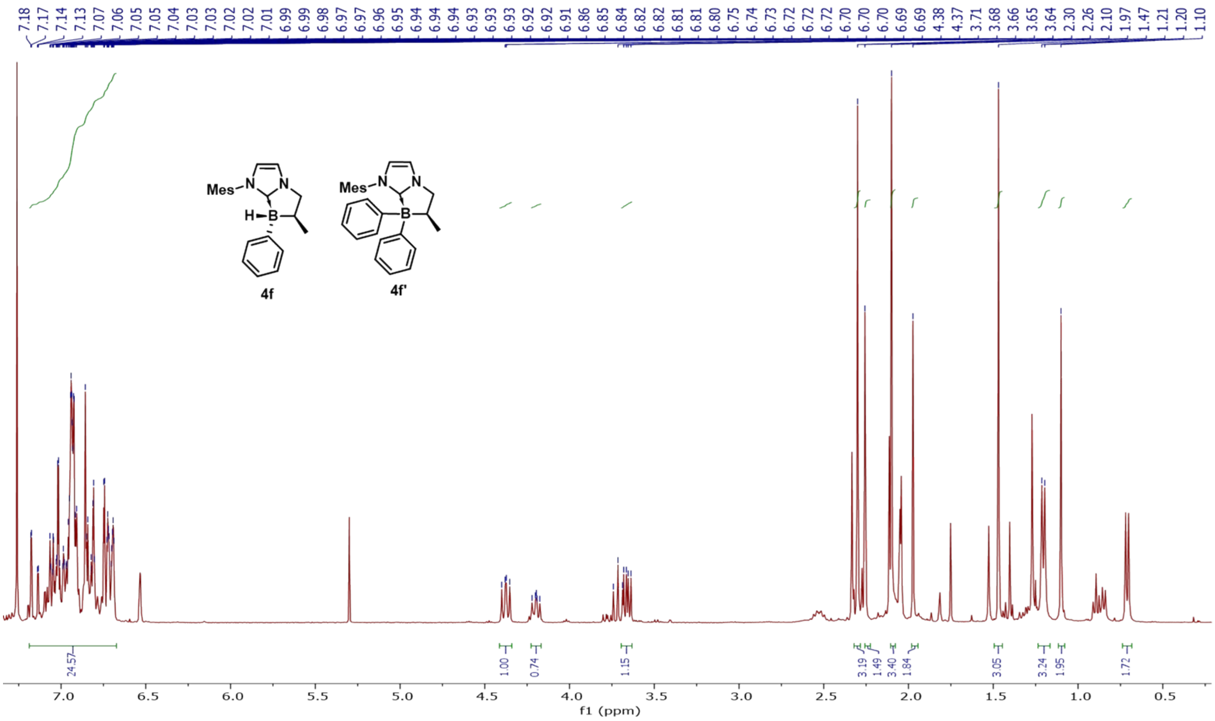

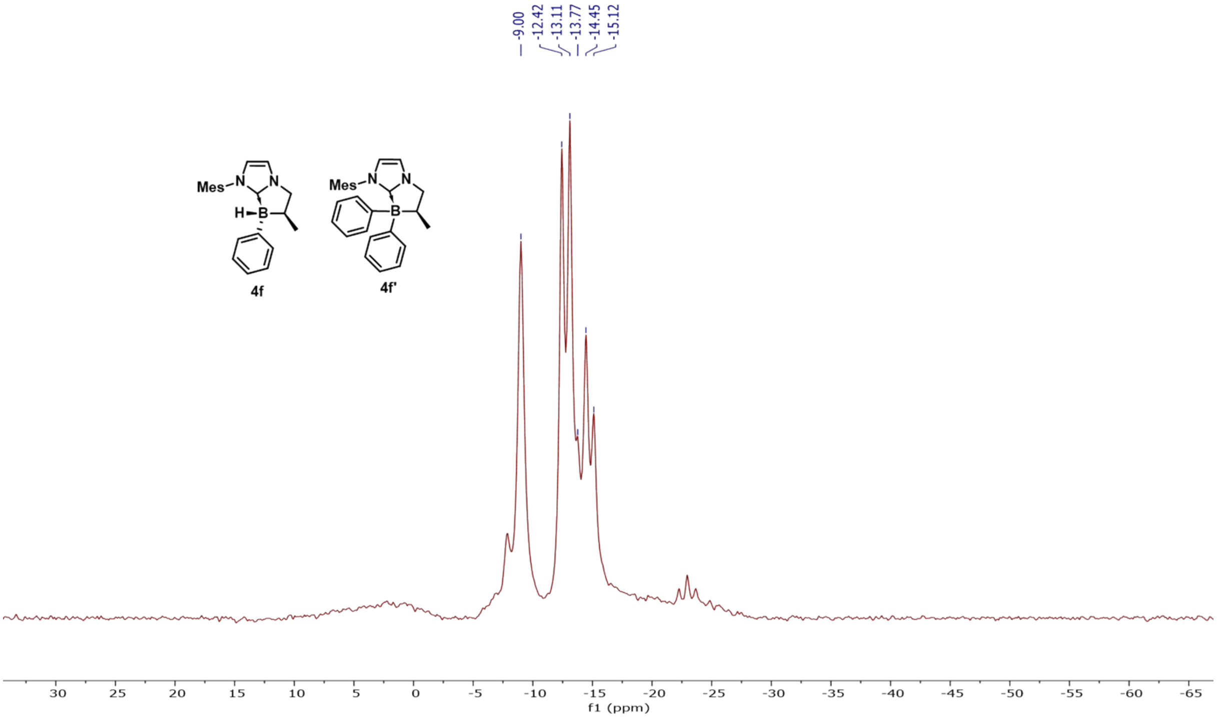

b. B-H bond-rhodium carbene insertion

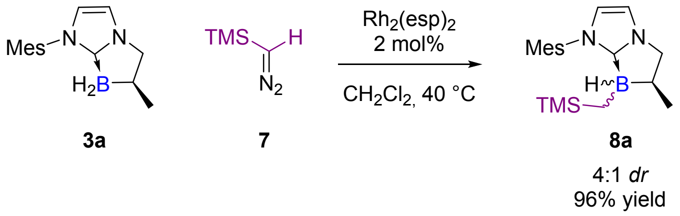

Procedure:

NHC-Borane **3a** (1.0 equiv, 0.62 mmol) and the $Rh_2(esp)_2$ (0.02 equiv, 9.5 mg) were dissolved in dry DCM (1 mL) under argon. The solution was green. The reaction mixture was heated to reflux. A solution of the (trimethylsilyl)methyldiazo compound (2.0 M in THF, 2.0 equiv, 0.62 mL) in dry DCM (0.8 mL) was added via syringe pump over a period of 4 h. The color of the solution turned orange. After 4 h, the mixture was filtered through a short plug of silica gel using dichloromethane as eluent and the solvent was removed under reduced pressure. The compound **8a** was obtained in 96% yield as two diastereomers (4:1 *dr*) and as a pale-yellow oil.

^{1}H NMR (400 MHz, $CDCl_3$) δ (ppm) 6.94 (bs, 3H), 6.72 (d, ^3J = 1.5 Hz, 1H), 4.18 (dd, ^2J = 10.6 Hz, ^3J = 7.9 Hz, 1H), 3.49 (dd, ^2J = 10.7 Hz, ^3J = 6.0 Hz, 1H), 2.33 (s, 3H), 2.03 (s, 6H), 1.52 – 1.39 (m, 1H), 1.08 (d, ^3J = 7.0 Hz, 1H), -0.24 (s, 9H), -0.99 (bs, 2H).

^{13}C NMR (101 MHz, $CDCl_3$) δ (ppm) 138.9, 135.8, 134.7, 134.0, 129.2, 128.8, 123.0, 116.4, 58.1, 33.3 $_{(HSQC)}$, 21.2, 21.1, 18.0, 17.9, 3.2 $_{(HSQC)}$, 0.8, C_9 is not observed.

^{11}B NMR (128 MHz, $CDCl_3$) δ (ppm) -16.2 (d, $^1J_{B\text{-}H}$ = 85.6 Hz), -17.5 (d, $^1J_{B\text{-}H}$ = 83.8 Hz).

^{29}Si NMR (79.5 MHz, CD_2Cl_2) δ (ppm) 3.3

HRMS (ES, $[M+Na]^+$) calcd for $[C_{19}H_{31}N_2BSiNa]^+$: 349.2246; found: 349.2240

^{1}H NMR (400 MHz, $CDCl_3$)

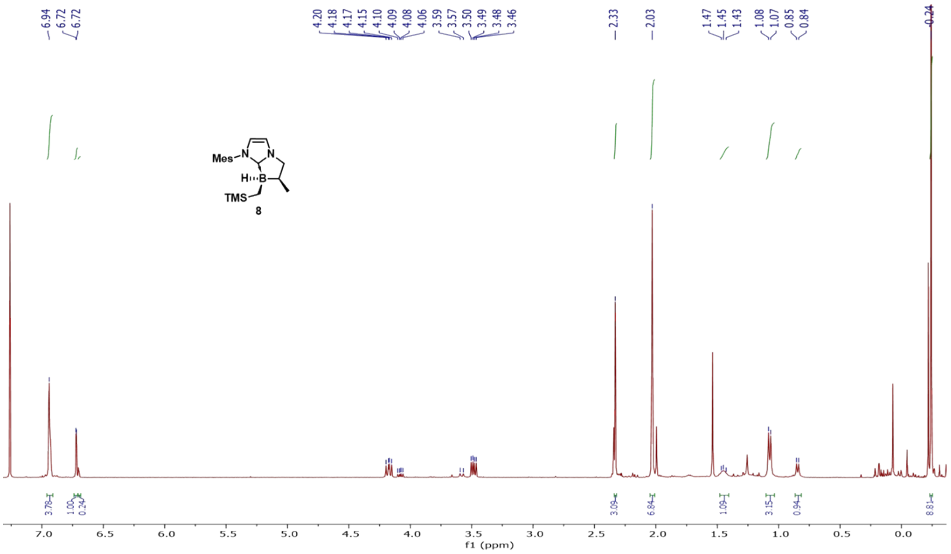

^{13}C NMR (101 MHz, $CDCl_3$)

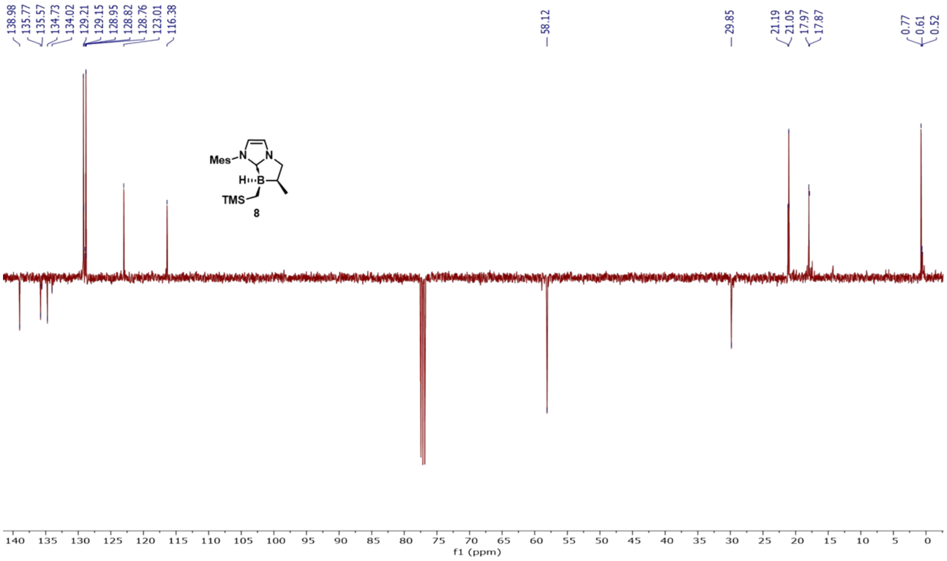

^{11}B NMR (128 MHz, $CDCl_3$)

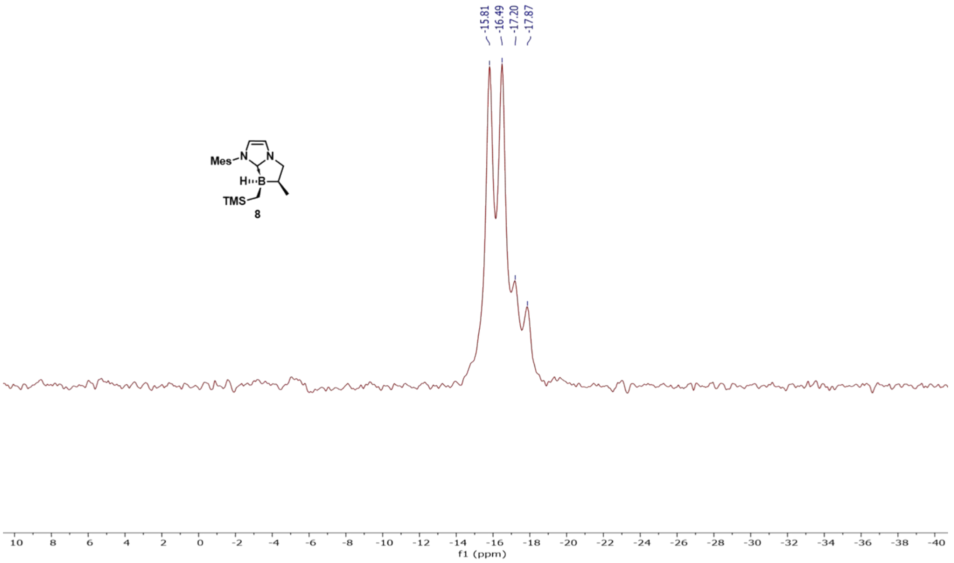

3. Procedure and Analytical Data

a. Synthetic procedures and spectroscopic data for the preparation of chlorinated NHC-borane **5**

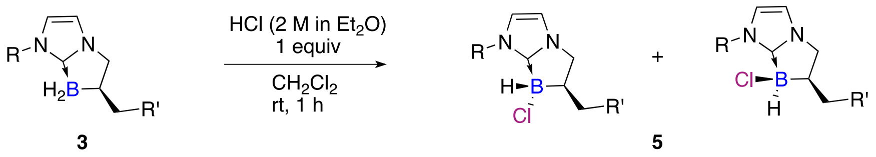

The appropriate NHC-borane **3** (0.25 mmol, 1 equiv) was dissolved in dry dichloromethane (2.5 mL) in a 10 mL Schlenk flask under argon. Then, 0.125 mL of HCl (2.0 M in Et_2O, 0.25 mmol, 1 equiv) was added and the mixture was stirred at room temperature under argon for 1 hour. The solution was then evaporated under vacuum to give a mixture of two diastereomers of monochlorinated NHC-borane **5**.

5a

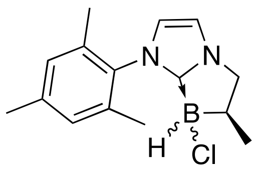

$C_{15}H_{20}BClN_2$
Mw: 274.60 g.mol^{-1}

quantitative yield; *dr* = 1.1:1

^{1}H NMR (400 MHz, $CDCl_3$) δ (ppm) 7.11 - 7.09 (m, 1H), 6.98 - 6.94 (m, 2H), 6.90 (d, ^3J = 1.4 Hz, 1H), 4.44 (dd, ^2J = 11.2, ^3J = 7.3 Hz, 1H), 3.61 (dd, ^2J = 11.2, ^3J = 3.2 Hz, 1H), 2.33 (s, 3H), 2.14 (s, 3H), 1.98 (s, 3H), 1.97 - 1.90 (m, 1H), 1.07 (d, ^3J = 7.4 Hz, 3H).

^{1}H NMR (400 MHz, $CDCl_3$) δ (ppm) 7.11 - 7.09 (m, 1H), 6.98 - 6.94 (m, 2H), 6.86 (d, ^3J = 1.5 Hz, 1H), 4.14 (dd, ^2J = 10.8, ^3J = 8.0 Hz, 1H), 3.77 (dd, ^2J = 10.8, ^3J = 8.9 Hz, 1H), 2.33 (s, 3H), 2.14 (s, 3H), 1.97 (s, 3H), 1.97 - 1.90 (m, 1H), 1.17 (d, ^3J = 7.0 Hz, 3H).

^{13}C NMR (101 MHz, $CDCl_3$) δ (ppm) 139.7, 135.3, 134.7, 129.6, 129.5, 129.2, 124.7, 124.2, 117.5, 117.31, 57.8, 56.8, $32.6_{(HSQC)}$, $29.1_{(HSQC)}$, 21.2, 19.3, 18.1, 17.6, 17.5, 14.7.

^{11}B NMR (128 MHz, $CDCl_3$) δ (ppm) -7.5 (bs).

HRMS (ESI, $[M+Na]^+$) calcd for $[C_{15}H_{20}N_2ClBNa]^+$: 297.1303; found: 297.1302

FT-IR (ATR) ν (cm^{-1}) 3136 (w), 2945 (m), 2924 (m), 2391 (m), 1606 (w), 1490 (s), 1037 (s), 746 (s), 566 (s)

5b

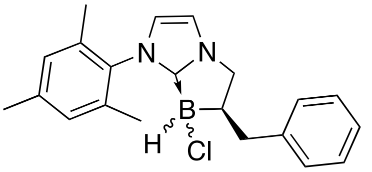

$C_{21}H_{24}BClN_2$
Mw: 350.69 g.mol^{-1}

quantitative yield; *dr* = 1.2:1

^{1}H NMR (400 MHz, $CDCl_3$) δ (ppm) 7.33 – 7.21 (m, 4H), 7.21 – 7.12 (m, 1H), 7.07 – 7.04 (m, 1H), 6.98 (s, 2H), 6.90 (d, ^3J = 1.7 Hz, 1H), 4.19 (dd, ^2J = 11.5 Hz, ^3J = 7.2 Hz, 1H), 3.70 (dd, ^2J = 11.6 Hz, ^3J = 3.8 Hz, 1H), 3.18 – 3.07 (m, 1H), 2.41 (dd, ^2J = 13.9, ^3J = 11.8 Hz, 1H), 2.34 (s, 3H), 2.31 – 2.17 (m, 1H), 2.14 (s, 3H), 2.03 (s, 3H).

^{1}**H NMR** (400 MHz, $CDCl_3$) δ (ppm) 7.33 – 7.21 (m, 4H), 7.21 – 7.12 (m, 1H), 7.07 – 7.04 (m, 1H), 6.98 (s, 1H), 6.95 (s, 1H), 6.87 (d, ^3J = 1.7 Hz, 1H), 4.03 – 3.80 (m, 2H), 3.18 – 3.07 (m, 1H), 2.79 (dd, ^2J = 14.0, ^3J = 10.1 Hz, 1H), 2.33 (s, 3H), 2.31 – 2.17 (m, 1H), 2.16 (s, 3H), 1.95 (s, 3H).

^{13}**C NMR** (101 MHz, $CDCl_3$) δ (ppm) 144.1, 143.6, 139.8, 135.3, 134.7, 133.0$_{(HSQC)}$, 129.6, 129.5, 129.2, 129.2, 128.9, 128.8, 128.3, 128.3, 125.6, 125.4, 124.8, 124.3, 117.5, 117.4, 54.9, 54.3, 39.9, 37.7$_{(HSQC)}$, 36.7, 21.2, 18.1, 18.1, 17.7, 17.5.

^{11}**B NMR** (128 MHz, $CDCl_3$) δ (ppm) -7.9 (bs).
HRMS (ESI, $[M+Na]^+$) not detected

FT-IR (ATR) ν (cm^{-1}) 3240 (m), 3145 (m), 2927 (m), 2860 (w), 1606 (w), 1454 (m), 1373 (s), 1197 (s), 1074 (s), 742 (s). The BH vibration band is not observed.

5c

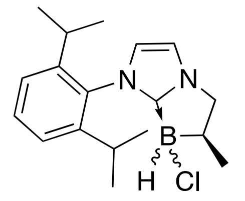

$C_{18}H_{26}BClN_2$
Mw: 316.68 g.mol^{-1}

quantitative yield; *dr* = 1:0.8

^{1}**H NMR** (400 MHz, $CDCl_3$) δ (ppm) 7.43 (t, ^3J = 7.7 Hz, 1H), 7.30 – 7.18 (m, 2H), 7.12 (brs, 1H), 6.90 (d, ^3J = 7.9 Hz, 1H), 4.45 (dd, ^2J = 11.1 Hz, ^3J = 6.7 Hz, 1H), 3.62 (dd, ^2J = 11.2 Hz, ^3J = 2.8 Hz, 1H), 2.63 (sex, ^3J = 6.7 Hz, 1H), 2.34 – 2.20 (m, 1H), 1.83 – 1.76 (m, 1H), 1.20– 0.89 (m, 15H).

^{1}**H NMR** (400 MHz, $CDCl_3$) δ (ppm) 7.43 (t, ^3J = 7.7 Hz, 1H), 7.30 – 7.18 (m, 2H), 7.12 (brs, 1H), 6.90 (d, ^3J = 7.9 Hz, 1H), 4.15 (t, ^2J = 9.8 Hz, 1H), 3.76 (t, ^2J = 9.8 Hz, 1H), 2.63 (sex, 1H), 2.34 – 2.20 (m, 1H), 1.83 – 1.76 (m, 1H), 1.20 – 0.89 (m, 15H).

^{13}**C NMR** (101 MHz, $CDCl_3$) δ (ppm) 146.2, 146.1, 145.7, 145.6, 132.7, 132.6, 130.5, 126.1, 125.6, 124.3, 124.2, 123.9, 123.9, 117.3, 117.0, 57.7, 56.6, $33.0_{(HSQC)}$, $28.5_{(HSQC)}$, 28.5, 25.8, 25.7, 24.8, 24.8, 23.5, 23.4, 23.0, 22.9, 19.1, $14.5_{(HMBC)}$.

^{11}**B NMR** (128 MHz, $CDCl_3$) δ (ppm) -7.5 (brs).

HRMS (ESI, $[M+Na]^+$) calcd for $[C_{18}H_{26}N_2BClNa]^+$: 339.1773 ; found : 339.1777

FT-IR (ATR) ν (cm^{-1}) 3158 (w), 3136 (w), 2963 (s), 2922 (m), 2864 (m), 2386 (m), 2342 (m), 1593 (w), 1551 (w), 1490 (m), 1463 (s), 1387 (m), 1358 (m), 1271 (m), 1140 (s), 1057 (s), 1011 (s), 974 (s), 814 (s), 785 (s), 759 (s), 646 (m).

5d

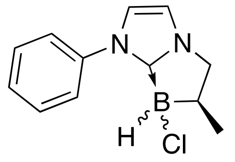

$C_{12}H_{14}BClN_2$
Mw: 232.52 g.mol^{-1}

quantitative yield; *dr* = 1:1

^{1}**H NMR** (300 MHz, CD_2Cl_2) δ (ppm) 7.83 (m, 2H), 7.53 (m, 2H), 7.47-7.43 (m, 1H), 7.42-7.36 (m, 1H), 7.17 – 7.13 (m, 1H), 4.39 (dd, ^2J = 11.4 Hz, ^3J = 7.1 Hz, 1H), 3.62 (dd, ^2J = 11.4 Hz, ^3J = 3.1 Hz, 1H), 1.99 – 1.84 (m, 1H), 1.06 (d, ^3J = 7.5 Hz, 3H).

^{1}**H NMR** (300 MHz, CD_2Cl_2) δ (ppm) 7.79 (m, 2H), 7.53 (m, 2H), 7.47-7.43 (m, 1H), 7.42-7.36 (m, 1H), 7.17 – 7.13 (m, 1H), 4.13 (dd, ^2J = 11.0 Hz, ^3J = 8.0 Hz, 1H), 3.71 (dd, ^2J = 11.0 Hz, ^3J = 9.4 Hz, 1H), 1.99 – 1.84 (m, 1H), 1.18 (d, ^3J = 7.0 Hz, 3H).

^{13}**C NMR** (75 MHz, CD_2Cl_2) δ (ppm) 137.6, 137.4, 129.6, 129.6, 128.3, 122.6, 122.4, 122.3, 122.0, 118.0, 117.9, 57.2, 56.1, $32.0_{(HSQC)}$, $29.1_{(HSQC)}$, 18.9, 14.2.

^{11}**B NMR** (128 MHz, CD_2Cl_2) δ (ppm) -6.4 (br t, ^1J = 112.7 Hz).

FT-IR (ATR) ν (cm^{-1}) 3116 (w), 2943 (w), 2428 (m), 2362 (m), 1597 (m), 1499 (s), 1447 (m), 1269 (m), 1142 (w), 972 (m), 918 (m), 760 (s), 690 (s), 592 (m).

HRMS (ESI, $[M+Na]^+$) not detected

b. Synthetic procedures and spectroscopic data for the preparation of NHC-borane **4**

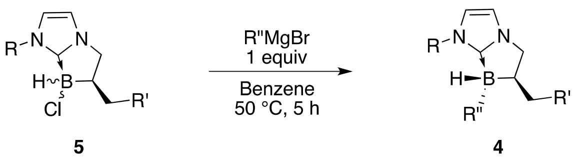

The appropriate chlorinated NHC-borane **5** (0.15 mmol, 1 equiv) was dissolved in dry benzene (1 mL) in a 10 mL Schlenk flask under argon. Then, 1 equivalent of Grignard reagent R”MgX (0.15 mmol) was added via a syringe at room temperature. The Schlenk was sealed and the reaction was stirred at 50 °C for 5 hours. The crude product was filtered through a short plug of silica gel using dichloromethane (10 mL) as eluent to give the desired NHC-borane **4**.

4a

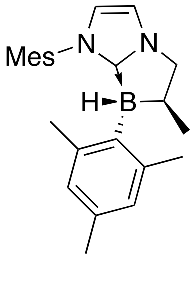

$C_{24}H_{31}BN_2$
Mw: 358.33 g.mol^{-1}

2,4,6-trimethylphenylmagnesium bromide (1.0 M in THF) was used in the process. The crude product was filtered through a short plug of silica gel using dichloromethane (10 mL) as eluent to give **4a** in 93% yield as one diastereomer.
Aspect: white solid.

^{1}H NMR (400 MHz, $CDCl_3$) δ (ppm) 7.01 (d, ^3J = 1.8 Hz, 1H), 6.83 (s, 1H), 6.81 (d, ^3J = 1.8 Hz, 1H), 6.67 (s, 1H), 6.55 (s, 2H), 4.48 (dd, ^2J = 11.2 Hz, ^3J = 9.2 Hz, 1H), 3.64 (dd, ^2J =

11.2 Hz, ^3J = 6.2 Hz, 1H), 2.64 (q, ^1J = 83.6 Hz, 1H), 2.24 (s, 3H), 2.15 (s, 3H), 2.09 – 2.03 (m, 10H), 1.73 (s, 3H), 1.18 (d, ^3J = 7.0 Hz, 3H).
^{13}C NMR (101 MHz, $CDCl_3$) δ (ppm) 141.1, 138.7, 135.6, 134.5, 133.6, 132.8, 128.8, 128.8, 127.4, 123.9, 116.4, 58.7, 29.5$_{(HSQC)}$, 24.1$_{(HSQC)}$, 23.1, 21.1, 20.9, 18.1, 17.3.

^{11}B NMR (128 MHz, $CDCl_3$) δ (ppm) -15.2 (d, ^1J = 83.6 Hz).

HRMS (ESI, $[M+Na]^+$) calcd for $[C_{24}H_{31}N_2BNa]^+$: 381.2477; found: 381.2480

$[\alpha]^{25}_D$: +66.4, c = 0.8, $CHCl_3$

FT-IR (ATR) ν (cm^{-1}) 3156 (w), 3125(m), 2951 (s), 2915 (s), 2853 (m), 2720 (w), 2353 (m), 1721 (w), 1670 (w), 1597 (m), 1543 (m), 1493 (s), 1449 (s), 1287 (m), 1217 (m), 1144 (m), 1032 (m), 1011 (m), 843 (s), 806 (m), 731 (s), 719 (s).

X-Ray: Crystals were obtained from slow evaporation of a product solution in DCM, layered with pentane.

4b

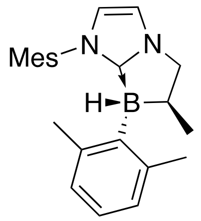

$C_{23}H_{29}BN_2$
Mw: 344.30 g.mol^{-1}

2,6-dimethylphenylmagnesium bromide (1.0 M in THF) was used in the process. The crude product was filtered through a short plug of silica gel using dichloromethane (10 mL) as eluent to give **4b** in 85% yield as one diastereomer.
Aspect: white solid.

^{1}H NMR (400 MHz, $CDCl_3$) δ (ppm) 7.02 (d, ^3J = 1.7 Hz, 1H), 6.85 – 6.79 (m, 2H), 6.76 (dd, ^3J = 8.3, 6.2 Hz, 1H), 6.72 – 6.68 (m, 2H), 6.65 (s, 1H), 4.48 (dd, ^2J = 11.3 Hz, ^3J = 9.7 Hz,

1H), 3.65 (dd, ^2J = 11.3 Hz, ^3J = 6.2 Hz, 1H), 2.22 (s, 3H), 2.17 – 2.01 (m, 10H), 1.69 (s, 3H), 1.18 (d, ^3J = 6.9 Hz, 3H).

^{13}C NMR (101 MHz, $CDCl_3$) δ (ppm) 144.6$_{(HSQC)}$, 142.1$_{(HSQC)}$, 138.8, 135.5, 134.5, 133.5, 132.8, 128.8, 128.7, 126.5, 124.0, 116.4, 58.7, 28.3$_{(HSQC)}$, 24.2$_{(HSQC)}$, 23.2, 21.0, 18.1, 17.1.

^{11}B NMR (128 MHz, $CDCl_3$) δ (ppm) -15.3 (d, ^1J = 84.6 Hz)

HRMS (ESI, $[M+Na]^+$) calcd for $[C_{23}H_{29}N_2BNa]^+$: 367.2320; found: 367.2317

$[\alpha]^{25}_D$: +35.4, c = 0.14, $CHCl_3$

X-Ray: Crystals were obtained from slow evaporation of a product solution in DCM, layered with pentane.

4c

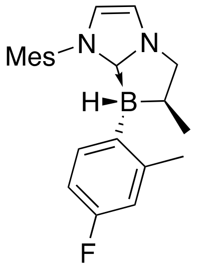

$C_{22}H_{26}BFN_2$
Mw: 348.26 g.mol^{-1}

2-Methyl-4-fluorophenylmagnesium bromide (0.5 M in THF) was used in the process. The crude product was filtered through a short plug of silica gel using dichloromethane (10 mL) as eluent. After evaporation the product was purified by flash chromatography using pentane:DCM 9:1 as eluent to give **4c** in 66% yield as two diastereomers (87:13 *dr*).
Aspect: white solid.

^{1}H NMR (400 MHz, $CDCl_3$) δ (ppm) 7.05 (d, ^3J = 1.5 Hz, 1H), 6.98 - 6.91 (m, 1H), 6.89 (s, 1H), 6.77 (d, ^3J = 1.5 Hz, 1H), 6.70 (s, 1H), 6.60 - 6.51 (m, 2H), 4.39 (dd, ^2J = 10.6 Hz, ^3J = 8.2 Hz, 1H), 3.66 (dd, ^2J = 10.8 Hz, ^3J = 5.6 Hz, 1H), 2.27 (s, 3H), 2.06 (s, 3H), 1.86 (s, 3H), 1.53 (s, 3H), 1.17 (d, ^3J = 6.9 Hz, 3H).

Remarkable signals of the minor diastereomer: 4.22 (dd, ^2J = 10.5 Hz, ^3J = 8.0 Hz, 1H), 3.77 (dd, ^2J = 10.7 Hz, ^3J = 5.7 Hz, 1H), 2.25 (s, 3H), 2.15 (s, 3H), 2.03 (s, 3H), 1.98 - 1.88 (m, 3H), 0.74 (d, ^3J = 7.2 Hz, 3H).

^{13}C NMR (101 MHz, $CDCl_3$) δ (ppm) 161.0 (d, $^1J_{CF}$ = 239.4 Hz), 144.6 (d, $^3J_{CF}$ = 6.1 Hz), 139.2, 139.0, 135.3, 135.0, 134.3, 133.7 (d, $^3J_{CF}$ = 7.0 Hz), 129.3, 128.9, 128.8, 124.0, 123.6, 116.8, 114.8 (d, $^2J_{CF}$ = 18.1 Hz), 110.1 (d, $^2J_{CF}$ = 18.1 Hz), 109.3 (d, $^3J_{CF}$ = 18.8 Hz), 58.5, 58.2, 32.1, 22.2, 21.6, 21.1, 18.1, 17.9, 17.3, 17.1.

^{11}B NMR (128 MHz, $CDCl_3$) δ (ppm) -14.9 (d, ^1J = 88.5 Hz), -15.9 (d, ^1J = 88.5 Hz)

^{19}F NMR (376 MHz, $CDCl_3$) δ (ppm) -121.7 (q, ^1J = 9.5 Hz), -121.9 (q, ^1J = 9.5 Hz).

HRMS (ESI, $[M+Na]^+$) calcd for $[C_{22}H_{26}N_2FBNa]^+$: 371.2069; found: 371.2070

4d

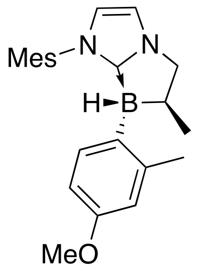

$C_{23}H_{29}BN_2O$
Mw: 360.30 g.mol^{-1}

2-methyl-4-methoxyphenylmagnesium bromide (0.5 M in THF) was used in the process. The crude product was filtered through a short plug of silica gel using dichloromethane (10 mL) as eluent. After evaporation the product was purified by flash chromatography using pentane:DCM 1:1 as eluent to give **4d** in 61% yield as two diastereomers (87:13 *dr*). Unfortunately, traces of bis-functionalized compound were found in mixture.

^{1}H NMR (400 MHz, $CDCl_3$) δ (ppm) 7.04 (d, ^3J = 1.5 Hz, 1H), 6.95 – 6.86 (m, 2H), 6.77 (d, ^3J = 1.5 Hz, 1H), 6.71 (s, 1H), 6.50 – 6.45 (m, 2H), 4.39 (dd, ^2J = 10.7 Hz, ^3J = 8.0 Hz, 1H), 3.72 (s, 3H), 3.65 (dd, ^2J = 10.9 Hz, ^3J = 5.1 Hz, 1H), 2.27 (s, 3H), 2.07 (s, 3H), 1.97 – 1.86 (m, 7H), 1.57 (s, 3H), 1.18 (d, ^3J = 7.0 Hz, 3H).

Remarkable signals of the minor diastereomer: 4.21 (dd, ^2J = 10.3 Hz, ^3J = 8.2 Hz, 1H), 2.25 (s, 3H), 2.15 (s, 3H), 2.04 (s, 3H), 1.90 (s, 3H), 1.01 (d, ^3J = 7.7 Hz, 3H).

^{13}C NMR (101 MHz, $CDCl_3$) δ (ppm) (major diastereomer) 156.7, 139.0, 138.9, 135.6, 135.1, 134.3, 128.9, 123.6, 116.8, 115.0, 114.5, 109.3, 58.6, 58.2, 55.1, 32.0, 23.1, 22.4, 21.9, 21.1, 18.1, 17.9.

^{11}B NMR (128 MHz, $CDCl_3$) δ (ppm) -14.7 (d, ^1J = 86.4 Hz), -15.9 (d, ^1J = 86.4 Hz).

HRMS (ESI, $[M+Na]^+$) calcd for $[C_{23}H_{29}N_2OBNa]^+$: 383.2269; found: 383.2267

4e

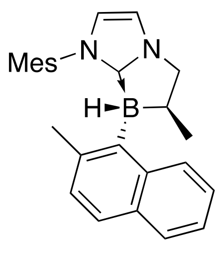

$C_{26}H_{29}BN_2^+$
Mw: 380.33 g.mol^{-1}

2-Methylnaphtylmagnesium bromide (0.25 M in THF) was used in the process. The crude product was filtered through a short plug of silica gel using dichloromethane (10 mL) as eluent. After evaporation the product was purified by flash chromatography using pentane:DCM 9:1 as eluent to give **4e** in 35% yield as one diastereomer.
Aspect: white solid

^{1}H NMR (400 MHz, $CDCl_3$) δ (ppm) 7.86 (bs, 1H), 7.58 (d, ^3J = 7.8 Hz, 1H), 7.33 (d, ^3J = 8.2 Hz, 1H), 7.20 – 7.06 (m, 3H), 7.04 (d, ^3J = 8.3 Hz, 1H), 6.80 (d, ^3J = 1.8 Hz, 1H), 6.64 (s, 1H), 6.35 (s, 1H), 4.56 (dd, ^2J = 10.6 Hz, ^3J = 9.4 Hz, 1H), 3.72 (dd, ^2J = 10.9 Hz, ^3J = 8.4 Hz, 1H), 3.03 (q, ^1J = 80.5 Hz, 1H, BH), 2.41 – 2.24 (m, 4H), 2.07 (s, 3H), 2.06 (s, 3H), 1.43 (s, 3H), 1.17 (d, ^3J = 6.7 Hz, 3H).

^{13}C NMR (101 MHz, $CDCl_3$) δ (ppm) $139.6_{(HMBC)}$, 138.4, 138.2, 135.3, 134.2, 133.1, 133.0, 132.2, 129.0, 128.9, 128.6, 128.4, 128.2, 124.4, 123.9, 123.3, 122.5, 116.5, 58.3, $31.3_{(HSQC)}$, 21.4, 20.9, 18.2, 17.5.

^{11}B NMR (128 MHz, $CDCl_3$) δ (ppm) -15.3 (d, ^{1}J = 84.3 Hz).

HRMS (ESI, $[M+Na]^+$) calcd for $[C_{26}H_{29}N_2BNa]^+$: 403.2321; found: 403.2321

$[\alpha]^{25}_{D}$: +107.4, c = 0.28, $CHCl_3$

4f

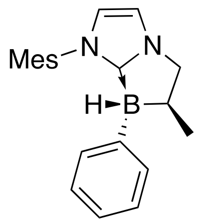

$C_{21}H_{25}BN_2$
Mw: 316.25 g.mol^{-1}

in mixture with

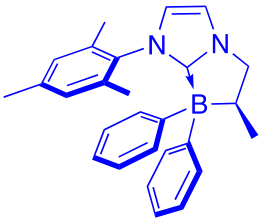

$C_{27}H_{29}BN_2$
Mw: 392.34 g.mol^{-1}

Phenyl Grignard reagent (1.0 M in THF) was used in the process. The crude product was filtered through a short plug of silica gel using dichloromethane (10 mL) as eluent. After evaporation the product was purified by flash chromatography using Pentane:DCM 9:1 as eluent to give **4f** as one diastereomer in mixture with the bis-functionalized product.

^{1}H NMR (400 MHz, $CDCl_3$) δ (ppm) 6.74 (d, ^{3}J = 1.8 Hz, 1H), 4.37 (dd, ^{2}J = 10.7 Hz, ^{3}J = 8.1 Hz, 1H), 3.66 (dd, ^{2}J = 10.8 Hz, ^{3}J = 6.5 Hz, 1H), 2.30 (s, 3H), 2.10 (s, 3H), 1.44 (s, 3H), 0.84 (d, ^{3}J = 6.8 Hz, 3H). The signals of the aromatic part are overlapping with the signals of the bis-functionalized product.

^{1}H NMR (400 MHz, $CDCl_3$) δ (ppm) 7.18 (d, ^{3}J = 1.8 Hz, 1H), 7.09 – 6.91 (m, 4H), 6.87 – 6.71 (m, 5H), 6.73 – 6.68 (m, 2H), 6.53 (s_{app}, 1H), 4.27 – 4.12 (m, 1H,), 3.71 (t_{app}, ^{3}J = 10.7 Hz, 1H), 2.60 – 2.44 (m, 1H), 2.25 (s, 3H), 1.97 (s, 3H), 1.09 (s, 3H), 0.70 (d, ^{3}J = 7.0 Hz, 3H).

^{11}**B NMR** (128 MHz, $CDCl_3$) δ (ppm) -8.9, -12.8 (d, $^1J_{BH}$ = 85.0 Hz)

4g

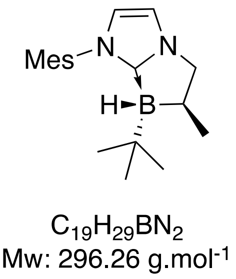

tert-Butylmagnesium bromide (0.75 M in THF) was used in the process. The crude product was filtered through a short plug of silica gel using dichloromethane (10 mL) as eluent to give **4g** in 74% yield as two diastereomers (67:33 *dr*).
Aspect: white solid

^{1}**H NMR** (400 MHz, $CDCl_3$) δ (ppm) 6.97 – 6.92 (m, 3H), 6.73 (d, ^3J = 1.8 Hz, 1H), 4.17 (dd, ^2J = 10.5 Hz, ^3J = 8.2 Hz, 1H), 3.47 (dd, ^2J = 10.5 Hz, ^3J = 7.6 Hz, 1H), 2.34 (s, 3H), 2.06 (s, 3H), 2.02 (s, 3H), 1.54 – 1.45 (m, 1H), 1.11 (d, ^3J = 6.9 Hz, 3H), 1.06 – 0.93 (m, 1H), 0.64 (s, 3H), 0.62 (s, 3H), 0.06 – 0.05 (m, 2H).

^{1}**H NMR** (400 MHz, $CDCl_3$) δ (ppm) 6.97 – 6.92 (m, 3H), 6.71 (d, ^3J = 1.7 Hz, 1H), 4.08 (dd, ^2J = 10.4 Hz, ^3J = 6.6 Hz, 1H), 3.59 (dd, ^2J = 10.4 Hz, ^3J = 1.1 Hz, 1H), 2.33 (s, 3H), 2.04 (s, 3H), 2.01 (s, 3H), 1.84 – 1.75 (m, 1H), 1.06 – 0.93 (m, 1H), 0.89 (d, ^3J = 7.2 Hz, 3H), 0.69 (s, 3H), 0.68 (s, 3H), 0.63 (d, J = 6.5 Hz), - 0.14 – - 0.22 (m, 2H).

^{13}**C NMR** (75 MHz, $CDCl_3$) δ (ppm) 139.9, 139.4, 136.2, 136.1, 135.8, 135.5, 135.3, 134.8, 134.7, 129.7, 129.6, 129.4, 129.2, 123.4, 123.3, 117.1, 116.9, 59.4, 58.5, $32.0_{(HSQC)}$, $28.8_{(HSQC)}$, 29.1, 28.9, 26.5, 26.4, 26.2, 25.8, 25.6, 21.6, 20.8, 18.4, 18.3, 18.3, 18.1, 17.9, 17.8.

^{11}**B NMR** (128 MHz, $CDCl_3$) δ (ppm) -14.8 (d, ^1J = 82.9 Hz), -16.5 (d, ^1J = 82.0 Hz).

FT-IR (ATR) ν (cm^{-1}) 3097 (w), 2951 (m), 2922 (m), 2353 (w), 1606 (w), 1496 (s), 1379 (s), 1064 (s), 817 (s), 665 (s)

HRMS (ESI, $[M+Na]^+$) not detected

4i

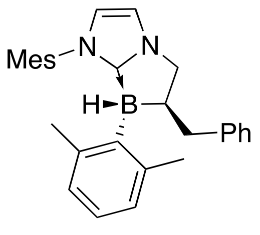

$C_{29}H_{33}BN_2$
Mw: 420.40 g.mol^{-1}

2,6-dimethylphenylmagnesium bromide (1.0 M in THF) was used in the process. The crude product was filtered through a short plug of silica gel using dichloromethane (10 mL) as eluent. After evaporation the product was purified by flash chromatography using pentane:DCM 9:1 as eluent to give **4i** in 63% yield as one diastereomer.
Aspect: white solid.

^{1}H NMR (400 MHz, $CDCl_3$) δ (ppm) 7.28 – 7.17 (m, 4H), 7.16 – 7.11 (m, 1H), 6.97 (d, ^3J = 1.8 Hz, 1H), 6.83 (s, 1H), 6.81 (d, ^3J = 1.7 Hz, 1H), 6.78 (dd, ^3J = 8.1 Hz, ^3J = 6.4 Hz, 1H), 6.74 – 6.69 (m, 2H), 6.63 (s, 1H), 4.21 (dd, ^2J = 11.6 Hz, ^3J = 9.2 Hz, 1H), 3.77 (dd, ^2J = 11.7 Hz, ^3J = 5.9 Hz, 1H), 3.09 (dd, ^2J = 14.3 Hz, ^3J = 4.0 Hz, 1H), 2.66 (dd, ^2J = 14.1 Hz, ^3J = 11.9 Hz, 1H), 2.54 – 2.44 (m, 1H), 2.23 (s, 3H), 2.08 (s, 3H), 2.07 (s, 6H), 1.64 (s, 3H).

^{13}C NMR (101 MHz, $CDCl_3$) δ (ppm) 145.4, 138.9, 135.5, 134.6, 133.4, 128.8, 128.7, 128.1, 126.6, 125.1, 124.2, 124.0, 116.5, 55.9, 44.6, $36.6_{(HSQC)}$, $24.3_{(HSQC)}$, 21.1, 18.1, 17.0.

^{11}B NMR (128 MHz, $CDCl_3$) δ (ppm) -16.1 (d, ^1J = 83.5 Hz).

HRMS (ESI, $[M+Na]^+$) calcd for $[C_{29}H_{33}N_2BNa]^+$: 443.2634; found: 443.2634

$[\alpha]^{25}_D$: -14.5, c = 0.1, $CHCl_3$

4j

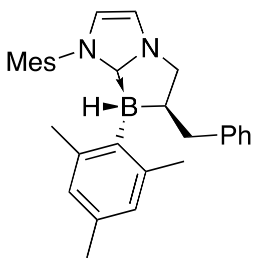

$C_{30}H_{35}BN_2$
Mw: 434.42 g.mol^{-1}

2,4,6-trimethylphenylmagnesium bromide (1.0 M in THF) was used in the process. The crude product was filtered through a short plug of silica gel using dichloromethane (10 mL) as eluent. After evaporation the product was purified by flash chromatography using pentane:DCM 9:1 as eluent to give **4j** in 56% yield as one diastereomer.
Aspect: white solid.

^{1}H NMR (400 MHz, $CDCl_3$) δ (ppm) 7.32 – 7.23 (m, 4H), 7.20 – 7.14 (m, 1H), 7.00 (d, ^3J = 1.7 Hz, 1H), 6.88 (s, 1H), 6.84 (d, ^3J = 1.6 Hz, 1H), 6.70 (s, 1H), 6.60 (s, 2H), 4.24 (dd, ^2J = 11.5 Hz, ^3J = 9.1 Hz, 1H), 3.80 (dd, ^2J = 11.7 Hz, ^3J = 5.9 Hz, 1H), 3.14 (dd, ^2J = 14.3 Hz, ^3J = 3.9 Hz, 1H), 2.69 (dd, ^2J = 13.8 Hz, ^3J = 11.9 Hz, 1H), 2.56 – 2.44 (s, 1H), 2.28 (s, 3H), 2.20 (s, 3H), 2.13 (s, 3H), 2.08 (s, 6H), 1.71 (s, 3H).

^{13}C NMR (101 MHz, $CDCl_3$) δ (ppm) 145.5, 142,3, 138.8, 135.5, 134.6, 133.5, 133.0, 128.8, 128.8, 128.1, 127.5, 125.1, 124.0, 116.5, 55.8, 44.5, 36.9$_{(HSQC)}$, 24.0$_{(HSQC)}$, 21.1, 21.0, 18.1, 17.1.

^{11}B NMR (128 MHz, $CDCl_3$) δ (ppm) -16.1 (d, ^1J = 81.7 Hz).

HRMS (ESI, $[M+Na]^+$) calcd for $[C_{30}H_{35}N_2BNa]^+$: 457.2791; found: 457.2791

$[\alpha]^{25}_D$: +19.4, c = 0.27, $CHCl_3$

4k

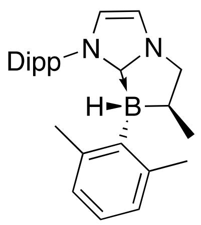

$C_{26}H_{35}BN_2$
Mw: 386.38 g.mol^{-1}

2,6-dimethylphenylmagnesium bromide (1.0 M in THF) was used in the process. The crude product was filtered through a short plug of silica gel using dichloromethane (10 mL) as eluent. After evaporation the product was purified by flash chromatography using pentane:DCM 9:1 as eluent to give **4k** in 38% yield as one diastereomer.
Aspect: Colorless oil.

^{1}H NMR (400 MHz, CD_2Cl_2) δ (ppm) 7.32 (t, ^3J = 7.8 Hz, 1H), 7.20 (dd, ^3J = 7.8 Hz, ^4J = 1.5 Hz, 1H), 7.08 (d, ^3J = 1.9 Hz, 1H), 6.98 (dd, ^3J = 7.8 Hz, ^4J = 1.5 Hz, 1H), 6.90 (d, ^3J = 1.8 Hz, 1H), 6.69 – 6.65 (m, 3H), 4.55 (dd, ^2J = 11.7 Hz, ^3J = 9.3 Hz, 1H), 3.69 (dd, ^2J = 11.6 Hz, ^3J = 5.0 Hz, 1H), 2.49 (sept, ^3J = 6.8 Hz, 1H), 2.35 (sept, ^3J = 6.8 Hz, 1H), 2.02 – 1.93 (m, 7H), 1.35 (d, ^3J = 6.8 Hz, 3H), 1.14 (d, ^3J = 7.1 Hz, 3H), 1.03 (d, ^3J = 6.9 Hz, 3H), 0.99 (d, ^3J = 6.8 Hz, 3H), 0.52 (d, ^3J = 6.8 Hz, 3H).

^{13}C NMR (101 MHz, CD_2Cl_2) δ (ppm) 146.8, 146.0, 134.0, 130.1, 127.4, 126.2, 124.2, 124.1, 123.7, 116.7, 59.2, 29.0$_{(HSQC)}$, 28.7, 26.0, 25.7, 24.3, 22.8, 21.9.

^{11}B NMR (128 MHz, CD_2Cl_2) δ (ppm) -15.5 (d, ^1J = 88.2 Hz).

HRMS (ESI, $[M+Na]^+$) calcd for $[C_{26}H_{35}N_2BNa]^+$: 409.2790; found: 409.2795

$[\alpha]^{25}_D$: +18.87, c = 0.53, $CHCl_3$

4l

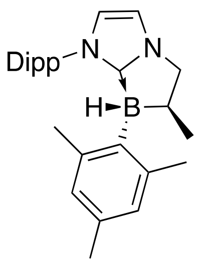

$C_{27}H_{37}BN_2$
Mw: 400.41 g.mol^{-1}

2,4,6-trimethylphenylmagnesium bromide (1.0 M in THF) was used in the process. The crude product was filtered through a short plug of silica gel using dichloromethane (10 mL) as eluent. After evaporation the product was purified by flash chromatography using pentane:DCM 8:2 as eluent to give **4l** in 38% yield as one diastereomer.
Aspect: Colorless oil.

^{1}H NMR (400 MHz, $CDCl_3$) δ (ppm) 7.31 (t, ^{3}J = 7.8 Hz, 1H), 7.18 (dd, ^{3}J = 7.8 Hz, ^{4}J = 1.4 Hz, 1H), 7.02 (d, ^{3}J = 1.8 Hz, 1H), 6.95 (dd, ^{3}J = 7.8 Hz, ^{4}J = 1.4 Hz, 1H), 6.86 (d, ^{3}J = 1.8 Hz, 1H), 6.54 (brs, 2H), 4.55 (dd, ^{2}J = 11.7 Hz, ^{3}J = 9.3 Hz, 1H), 3.69 (dd, ^{2}J = 11.6 Hz, ^{3}J = 4.4 Hz, 1H), 2.42 (sep, ^{3}J = 6.8 Hz, 1H), 2.28 (sep, ^{3}J = 6.8 Hz, 1H), 2.10 (s, 3H), 2.03 – 1.93 (m, 7H), 1.35 (d, ^{3}J = 6.8 Hz, 3H), 1.15 (d, ^{3}J = 7.1 Hz, 3H), 1.04 (d, ^{3}J = 6.9 Hz, 3H), 0.96 (d, ^{3}J = 6.9 Hz, 3H), 0.46 (d, ^{3}J = 6.7 Hz, 3H).

^{13}C NMR (101 MHz, $CDCl_3$) δ (ppm) 146.4, 145.7, 133.6, 132.9, 129.9, 127.7, 125.7, 123.8, 123.4, 116.1, 59.2, $28.7_{(HSQC)}$, 28.4, 26.1, 25.6, 24.7, $24.5_{(HSQC)}$, $23.0_{(HSQC)}$, 22.9, 21.5, 20.9.

^{11}B NMR (128 MHz, $CDCl_3$) δ (ppm) -15.61 (d, ^{1}J = 88.5 Hz).

HRMS (ESI, $[M+Na]^+$) calcd for $[C_{27}H_{37}N_2BNa]^+$: 423.2947; found: 423.2947

$[\alpha]^{25}_{D}$: +18.29, c = 0.35, $CHCl_3$

4m

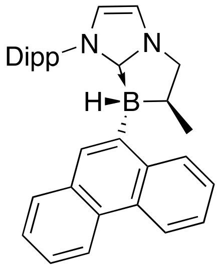

$C_{32}H_{35}BN_2$
Mw: 458.44 g.mol^{-1}

9-Phenanthrylmagnesium bromide (0.5 M in THF) was used in the process. The crude product was filtered through a short plug of silica gel using dichloromethane (10 mL) as eluent. After evaporation the product was purified by flash chromatography using pentane:DCM 7:3 as eluent to give **4m** in 57% yield as one diastereomer.
Aspect: Colorless oil.

^{1}H NMR (300 MHz, CD_2Cl_2) δ (ppm) 8.59 – 8.50 (m, 2H), 8.17 (dd, ^{3}J = 8.3 Hz, ^{4}J = 1.4 Hz, 1H), 7.68 – 7.61 (m, 1H), 7.49 – 7.37 (m, 3H), 7.35 (s, 1H), 7.24 (d, ^{3}J = 1.2 Hz, 1H), 7.24 – 7.21 (m, 3H), 6.95 (d, ^{3}J = 1.8 Hz, 1H), 6.82 – 6.62 (m, 1H), 4.51 (dd, ^{2}J = 11.0 Hz, ^{3}J = 7.2 Hz, 1H), 3.75 (dd, ^{2}J = 11.0, ^{3}J = 4.1 Hz, 1H), 2.64 (sep, ^{3}J = 6.8 Hz, 1H), 2.44 (p, ^{3}J = 6.8 Hz, 1H), 2.09 – 1.94 (m, 1H), 1.46 (d, ^{3}J = 6.8 Hz, 3H), 1.23 (d, ^{3}J = 7.2 Hz, 3H), 1.09 (d, ^{3}J = 6.9 Hz, 3H), 0.95 (d, ^{3}J = 6.8 Hz, 3H), 0.14 (d, ^{3}J = 6.8 Hz, 3H).

^{13}C NMR (75 MHz, CD_2Cl_2) δ (ppm) 146.5, 145.8, 137.6, 134.1, 133.0, 130.3, 130.0, 129.9, 129.8, 127.9, 126.0, 125.9, 125.5, 124.9, 124.6, 124.3, 123.9, 122.4, 122.3, 117.5, 58.8, $32.3_{(HSQC)}$, 28.8, 28.6, 26.0, 25.7, 23.1, 21.8.

^{11}B NMR (128 MHz, CD_2Cl_2) δ (ppm) -14.29 (d, ^{1}J = 91.5 Hz).

HRMS (ESI, $[M+Na]^+$) calcd for $[C_{32}H_{35}BN_2Na]^+$: 481.2791; found: 481.2790

$[\alpha]^{25}_{D}$: +4.64, c = 0.14, $CHCl_3$

4n

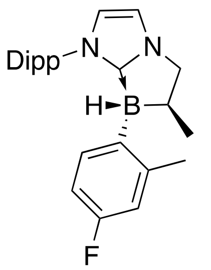

$C_{25}H_{32}BFN_2$
Mw: 229.08 g.mol^{-1}

2-Methyl-4-fluorophenylmagnesium bromide (0.5 M in THF) was used in the process. The crude product was filtered through a short plug of silica gel using dichloromethane (10 mL) as eluent. After evaporation the product was purified by flash chromatography using pentane:DCM 8 :2 as eluent to give **4n** in 43% yield as two diastereomers (95:5 *dr*).
Aspect: Colorless oil.

^{1}H NMR (400 MHz, CD_2Cl_2) δ (ppm) 7.37 (t, ^3J = 7.8 Hz, 1H), 7.23 (dd, ^3J = 7.8 Hz, ^4J = 1.5 Hz, 1H), 7.12 (d, ^3J = 1.8 Hz, 1H), 7.06 (dd, ^3J = 7.8 Hz, ^4J = 1.5 Hz, 1H), 6.95 – 6.89 (m, 1H), 6.89 (d, ^3J = 1.8 Hz, 1H), 6.57 – 6.50 (m, 2H), 4.41 (dd, ^2J = 11.1 Hz, ^3J = 7.7 Hz, 1H), 3.66 (dd, ^2J = 11.1 Hz, ^3J = 5.1 Hz, 1H), 2.54 (sep, ^3J = 6.8 Hz, 1H), 2.27 (sep, ^3J = 6.8 Hz, 1H), 1.85 (brs, 3H), 1.81 – 1.77 (m, 1H), 1.31 (d, ^3J = 6.8 Hz, 3H), 1.12 (d, ^3J = 6.4 Hz, 3H), 1.05 (d, ^3J = 6.9 Hz, 3H), 0.99 (d, ^3J = 6.8 Hz, 3H), 0.62 (d, ^3J = 6.8 Hz, 3H).

^{13}C NMR (101 MHz, CD_2Cl_2) δ (ppm) 162.4, 160.0, 146.6, 146.0, 144.8, 134.0, 130.3, 125.8, 124.3, 123.9, 117.1, 115.0, 110.6, 58.6, 32.8$_{(HSQC)}$, 28.7, 26.1, 25.5, 22.9, 22.5, 21.9, 21.7.
^{11}B NMR (128 MHz, CD_2Cl_2) δ (ppm) -14.85 (d, ^1J = 90.4 Hz).

^{19}F NMR (376 MHz, CD_2Cl_2) δ (ppm) -122.37.

HRMS (ESI, $[M+Na]^+$) calcd for $[C_{25}H_{32}N_2BFNa]^+$: 413.2539; found: 413.2546

FT-IR (ATR) ν (cm^{-1}) 3132 (w), 2961 (s), 2928 (s), 2870 (m), 2851 (m), 2359 (m), 2338 (m), 1578 (m), 1483 (s), 1360 (w), 1269 (m), 1223 (s), 1038 (m), 947 (m), 853 (m), 733 (s)

4o

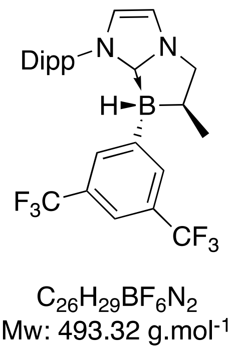

3,5-Bis(trifluoromethyl)phenylmagnesium bromide (0.5 M in THF) was used in the process. The crude product was filtered through a short plug of silica gel using dichloromethane (10 mL) as eluent. After evaporation the product was purified by flash chromatography using Pentane:DCM 8:2 as eluent to give **4o** in 14% yield as one diastereomer.

^{1}H NMR (400 MHz, $CDCl_3$) δ (ppm) 7.40 – 7.34 (m, 2H), 7.33 – 7.29 (m, 3H), 7.07 (d, ^{3}J = 1.8 Hz, 1H), 6.91 (dd, ^{3}J = 7.7 Hz, ^{4}J = 1.5 Hz, 1H), 6.81 (d, ^{3}J = 1.8 Hz, 1H), 4.38 (dd, ^{2}J = 10.7 Hz, ^{3}J = 8.8 Hz, 1H), 3.70 (dd, ^{2}J = 10.7 Hz, ^{3}J = 8.8 Hz, 1H), 2.61 (sep, ^{3}J = 6.9 Hz, 1H), 2.25 – 2.14 (m, 1H), 1.92 (sep, ^{3}J = 6.8 Hz, 1H), 1.42 (d, ^{3}J = 6.8 Hz, 3H), 1.19 (d, ^{3}J = 6.8 Hz, 3H), 1.10 (d, ^{3}J = 6.9 Hz, 3H), 0.90 (d, ^{3}J = 6.8 Hz, 3H), 0.23 (d, ^{3}J = 6.8 Hz, 3H).

^{13}C NMR (101 MHz, $CDCl_3$) δ (ppm) 145.8, 145.3, 133.6, 132.8, 130.7, 129.2, 125.1, 124.1, 123.7, 118.1, 116.5, 57.5, $30.2_{(HSQC)}$, 28.4, 28.2, 25.6, 25.4, 23.1, 21.3, 19.1.

^{11}B NMR (128 MHz, $CDCl_3$) δ (ppm) -13.47 (d, ^{1}J = 88.1 Hz).

^{19}F NMR (376 MHz, $CDCl_3$) δ (ppm) -62.39.

HRMS (ESI, $[M+Na]^+$) calcd for $[C_{26}H_{29}N_2BF_6Na]^+$: 517.2225; found: 517.2218

$[\alpha]^{25}_{D}$: -39.68, c = 0.15, $CHCl_3$

4p

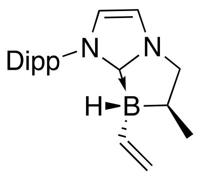

$C_{20}H_{29}BN_2$
Mw: 308.27 g.mol^{-1}

Vinylmagnesium bromide (0.5 M in THF) was used in the process. The crude product was filtered through a short plug of silica gel using dichloromethane (10 mL) as eluent to afford the product **4p** as two diastereomers (81:19 *dr*) in mixture with the bis-functionalized product. Unfortunately, the product decomposed during column chromatography.

^{1}H NMR (400 MHz, CD_2Cl_2) δ (ppm) 7.45 (t, ^{3}J = 7.6 Hz, 1H), 7.25 (t_{app}, J = 7.8 Hz, 2H), 7.04 (d, ^{3}J = 1.9 Hz, 1H), 6.83 (d, ^{3}J = 1.9 Hz, 1H), 5.89 – 5.71 (m, 1H), 4.99 – 4.78 (m, 2H), 4.23 (dd, ^{2}J = 10.7 Hz, ^{3}J = 8.1 Hz, 1H), 3.54 (dd, ^{2}J = 10.7 Hz, ^{3}J = 7.7 Hz, 1H), 2.53 (sep, ^{3}J = 6.9 Hz, 1H), 2.44 (sep, ^{3}J = 6.8 Hz, 1H), 1.78 – 1.69 (m, 1H), 1.25 (d, ^{3}J = 6.8, 4.1 Hz, 3H), 1.17 (d, ^{3}J = 6.8 Hz, 3H), 1.13 – 1.03 (m, 9H).

^{11}B NMR (128 MHz, CD_2Cl_2) δ (ppm) -14.8 (d, ^{1}J = 88.2 Hz).

4r

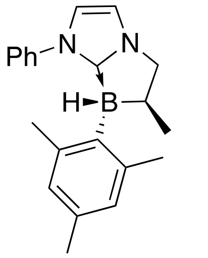

$C_{21}H_{25}BN_2$
Mw: 316.25 g.mol^{-1}

2,4,6-trimethylphenylmagnesium bromide (1.0 M in THF) was used in the process. The crude product was filtered through a short plug of silica gel using dichloromethane (10 mL) as eluent. After evaporation the product was purified by flash chromatography using pentane:DCM 8:2 as eluent to give **4r** in 89% yield as two diastereomers (92:8 *dr*).
Aspect: Colorless oil.

^{1}**H NMR** (400 MHz, CD_2Cl_2) δ (ppm) 7.51-7.45 (m, 2H), 7.33 (d, ^{3}J = 1.98 Hz, 1H), 7.28-7.17 (m, 3H), 7.08 (d, ^{3}J = 2.0 Hz, 1H), 6.82 (brs, 1H), 6.66 – 6.54 (m, 1H), 4.35 (dd, ^{2}J = 11.0 Hz, ^{3}J = 8.8 Hz, 1H), 3.62 (dd, ^{2}J = 10.8 Hz, ^{3}J = 8.2 Hz, 1H), 3.00 (q, ^{1}J = 86.4 Hz, 1H), 2.29 (brs, 6H), 2.15 (brs, 3H), 1.88 – 1.84 (m, 1H), 1.21 (d, ^{3}J = 6.9 Hz, 3H).

^{13}**C NMR** (75 MHz, CD_2Cl_2) δ (ppm) 138.1, 138.0, 133.2, 129.6, 129.5, 129.4, 129.4, 128.6, 128.0, 127.6, 121.6, 121.5, 120.8, 117.8, 58.1, $31.8_{(HSQC)}$, 21.4, 21.0, 20.9, 19.9.

^{11}**B NMR** (128 MHz, CD_2Cl_2) δ (ppm) -14.9 (d, ^{1}J = 86.2 Hz).

HRMS (ESI, $[M+Na]^+$) calcd for $[C_{21}H_{25}BN_2Na]^+$: 339.2007; found: 339.2003.

FT-IR (ATR) ν (cm^{-1}) 3132 (w), 2918 (m), 2853 (m), 2313 (w), 2280 (w), 1597 (m), 1501 (s), 1435 (m), 1267 (m), 1198 (m), 1061 (m), 962 (m), 760 (s), 691 (s).

4s

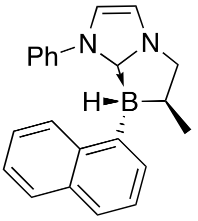

$C_{22}H_{21}BN_2$
Mw: 324.23 g.mol^{-1}

1-Naphtylmagnesium bromide (0.25 M in THF) was used in the process. The crude product was filtered through a short plug of silica gel using dichloromethane (10 mL) as eluent. After evaporation the product was purified by flash chromatography using pentane:DCM 8:2 as eluent to give **4s** in 74% yield as two diastereomers (87:13 *dr*).
Aspect: Colorless oil.

^{1}**H NMR** (300 MHz, CD_2Cl_2) δ (ppm) 8.38 – 8.30 (m, 1H), 7.74 – 7.68 (m, 1H), 7.61 – 7.55 (m, 2H), 7.50 – 7.45 (m, 2H), 7.38 – 7.33 (m, 2H), 7.25 – 7.18 (m, 4H), 7.08 (dd, ^{2}J = 8.1 Hz, ^{3}J = 6.7 Hz, 1H), 6.77 (d, ^{3}J = 6.7 Hz, 1H), 4.25 (dd, ^{2}J = 10.9 Hz, ^{3}J = 7.5 Hz, 1H), 3.65 (dd, ^{2}J = 11.0 Hz, ^{3}J = 4.6 Hz, 1H), 3.15 (q, ^{1}J = 92.1 Hz, 1H), 1.84 – 1.72 (m, 1H), 1.27 (d, ^{3}J = 8.2 Hz, 3H).

^{1}H NMR (300 MHz, CD_2Cl_2) δ (ppm) 8.60-8.50 (m, 1H), 7.74 – 7.68 (m, 1H), 7.61 – 7.55 (m, 2H), 7.50 – 7.45 (m, 2H), 7.38 – 7.33 (m, 2H), 7.25 – 7.18 (m, 4H), 6.97 (dd, ^2J = 8.1 Hz, ^3J = 6.7 Hz, 1H), 6.57 (d, ^3J = 6.7 Hz, 1H), 4.31-4.24(m, 1H), 3.72 (dd, ^2J = 10.9 Hz, ^3J = 5.1 Hz, 1H), 3.15 (q, ^1J = 92.1 Hz, 1H), 2.39 – 2.25 (m, 1H), 0.51 (d, ^3J = 7.3 Hz, 3H).

^{13}C NMR (75 MHz, CD_2Cl_2) δ (ppm) 138.2, 134.1, 130.8, 129.9, 129.8, 129.6, 128.4, 128.3, 128.0, 127.9, 125.6, 124.8, 124.6, 124.6, 124.2, 124.1, 122.5, 122.3, 122.1, 121.8, 118.2, 58.3, $32.8_{(HSQC)}$, $27.9_{(HSQC)}$, 21.7, 18.3.

^{11}B NMR (128 MHz, CD_2Cl_2) δ (ppm) -12.6 (d, ^1J = 90.6 Hz), -14.9 (d, ^1J = 89.0 Hz).

HRMS (ESI, $[M+Ag]^+$) calcd for $[C_{22}H_{21}BN_2Ag]^+$: 431.0847; found: 431.0846.

FT-IR (ATR) ν (cm^{-1}) 3163 (w), 2928 (m), 2851 (m), 2324 (m), 1599 (m), 1502 (s), 1445 (m), 1186 (w), 1065 (w), 956 (w), 783 (s), 679 (s).

4t

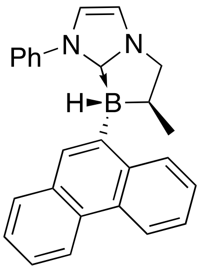

$C_{26}H_{23}BN_2$
Mw: 374.28 $g.mol^{-1}$

9-Phenanthryl Grignard reagent (0.5 M in THF) was used in the process. The crude product was filtered through a short plug of silica gel using dichloromethane (10 mL) as eluent. After evaporation the product was purified by flash chromatography using pentane:DCM 8:2 as eluent to give **4t** in 52% yield as two diastereomers (85:15 *dr*).

^{1}H NMR (300 MHz, CD_2Cl_2) δ (ppm) 8.73 – 8.65 (m, 1H), 8.64 – 8.57 (m, 1H), 8.53 – 8.46 (m, 1H), 7.70 – 7.10 (m, 12H), 7.00 (brs, 1H), 4.25 (dd, ^2J = 11.2 Hz, ^3J = 7.5 Hz, 1H), 3.65 (dd, ^2J = 11.2 Hz, ^3J = 3.9 Hz, 1H), 3.06 (q, ^1J = 100.0 Hz, 1H), 1.80 – 1.62 (m, 1H), 1.18 (d, ^3J = 7.1 Hz, 3H).

^{1}**H NMR** (300 MHz, CD_2Cl_2) δ (ppm) 8.73 – 8.65 (m, 1H), 8.64 – 8.57 (m, 1H), 8.53 – 8.46 (m, 1H), 7.70 – 7.10 (m, 12H), 6.83 (bs, 1H), 4.31 (dd, ^{2}J = 10.4 Hz, ^{3}J = 7.5 Hz, 1H), 3.60 (dd, ^{2}J = 11.0 Hz, ^{3}J = 4.3 Hz, 1H), 3.06 (q, ^{1}J = 100.0 Hz, 1H), 1.78 – 1.65 (m, 1H), 0.42 (d, ^{3}J = 7.3 Hz, 3H).

^{13}**C NMR** (75 MHz, CD_2Cl_2) δ (ppm) 138.3, 137.6, 132.9, 131.0,130.4, 130.3, 129.7, 129.7, 129.6, 129.6, 128.1, 128.0, 127.7, 127.5, 126.1, 126.0, 125.8, 125.7, 125.4, 125.3, 124.8, 124.6, 122.8, 122.7, 122.6, 122.5, 122.5, 122.0, 121.9, 118.3, 118.2, 58.4, 58.3, $32.2_{(HSQC)}$, $27.7_{(HSQC)}$, 22.0, 18.5.

^{11}**B NMR** (128 MHz, CD_2Cl_2) δ (ppm) -12.4 (d, ^{1}J = 91.1 Hz), -14.5 (d, ^{1}J = 90.3 Hz).

HRMS (ESI, $[M+Na]^+$) calcd for $[C_{26}H_{23}BN_2Na]^+$: 397.1851; found: 397.1852.

FT-IR (ATR) ν (cm^{-1}) 3067 (w), 2918 (m), 2853 (m), 2309 (w), 1591 (w), 1497 (m), 1441 (m), 1134 (w), 1069 (w), 731 (s), 687 (s).

4. NMR Spectra

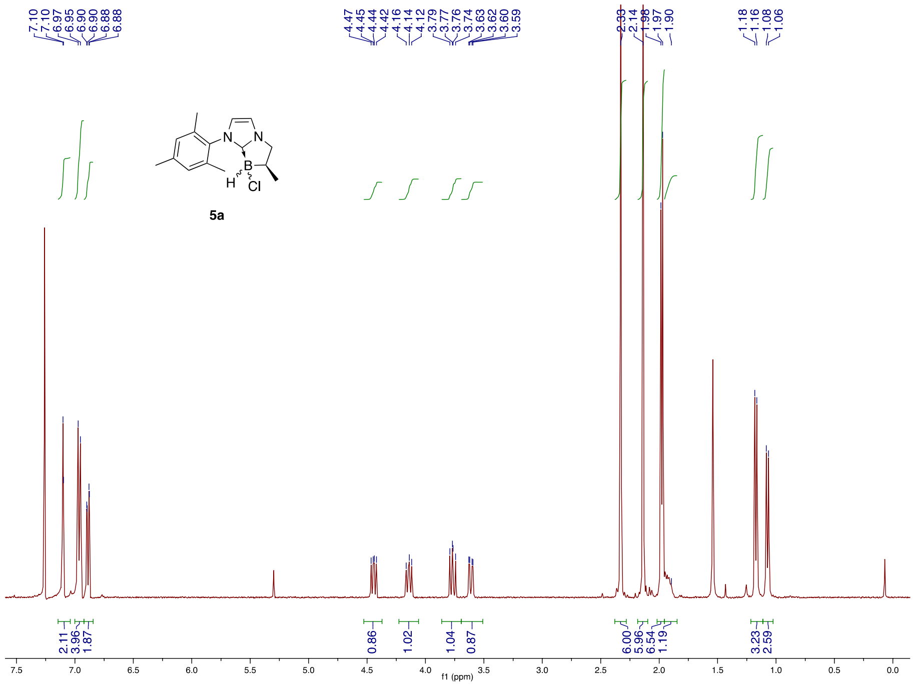

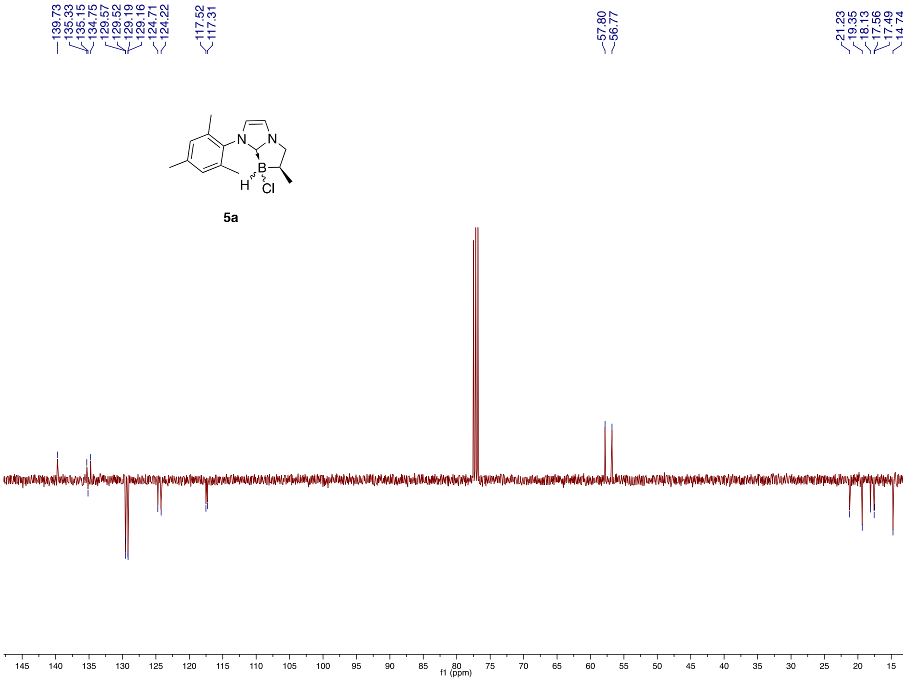

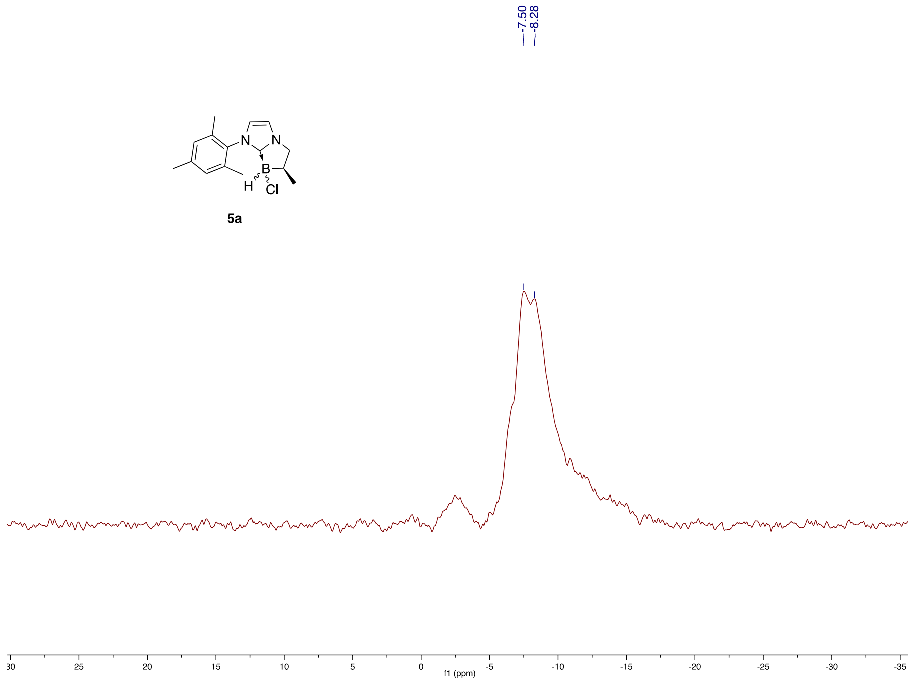
-7.50
-8.28
N
N
B
H
Cl
f1 (ppm)

5a

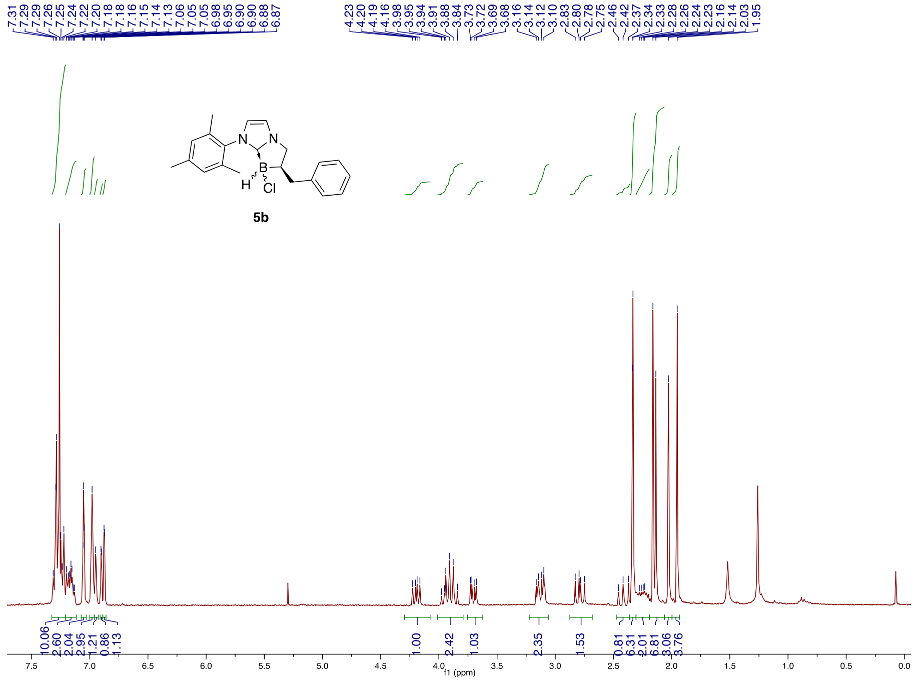
7.31
7.29
7.26
7.25
7.24
7.22
7.20
7.18
7.16
7.15
7.14
7.13
7.06
7.05
6.98
6.95
6.90
6.88
6.87
4.23
4.20
4.19
4.16
3.95
3.94
3.91
3.88
3.84
3.73
3.72
3.69
3.68
3.16
3.14
3.12
3.10
2.83
2.80
2.78
2.75
2.46
2.42
2.37
2.34
2.33
2.28
2.26
2.24
2.23
2.16
2.14
2.03
1.95
N
N
B
H
Cl
10.06
2.60
2.04
2.95
1.21
0.86
1.13
1.00
2.42
1.03
2.35
1.53
0.81
6.31
2.01
6.81
3.06
3.76
f1 (ppm)

5b

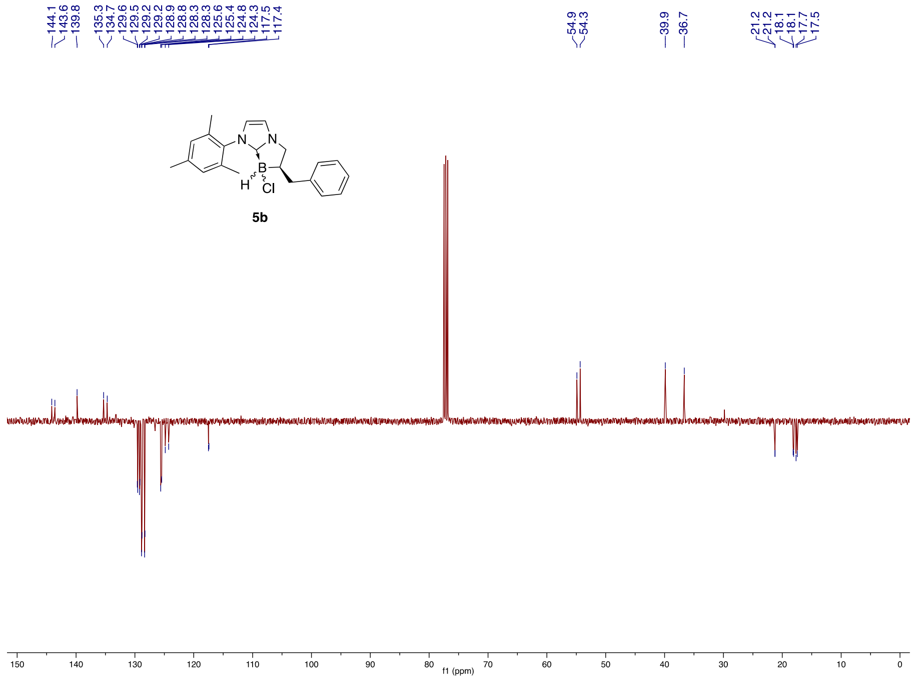

144.1
143.6
139.8
135.3
134.7
129.6
129.5
129.2
129.2
128.9
128.8
128.3
128.3
125.6
125.4
124.8
124.3
117.5
117.4
54.9
54.3
39.9
36.7
21.2
21.2
18.1
17.7
17.5
5b
f1 (ppm)

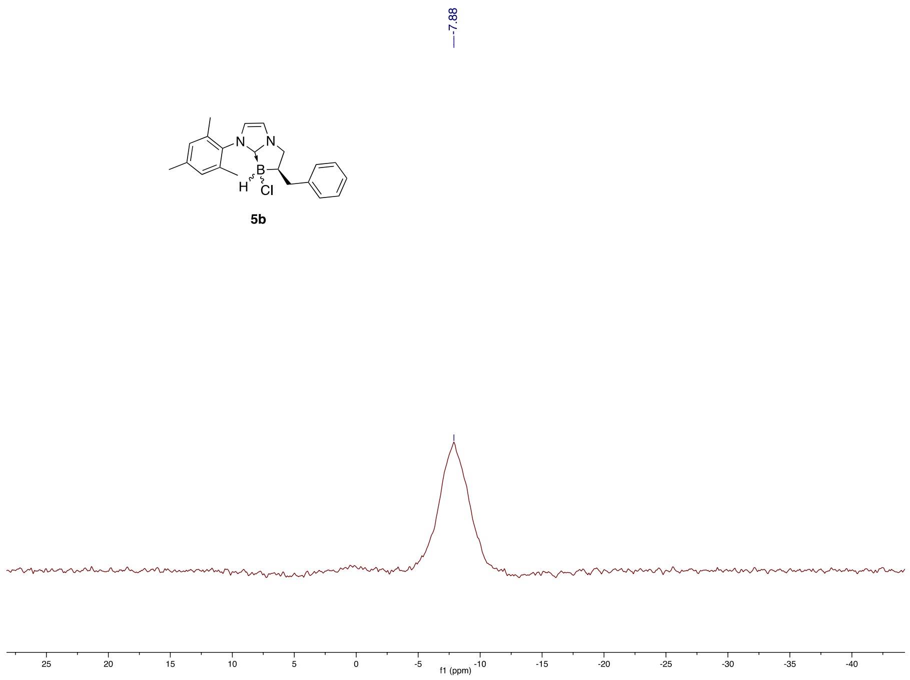

-7.88
5b
f1 (ppm)

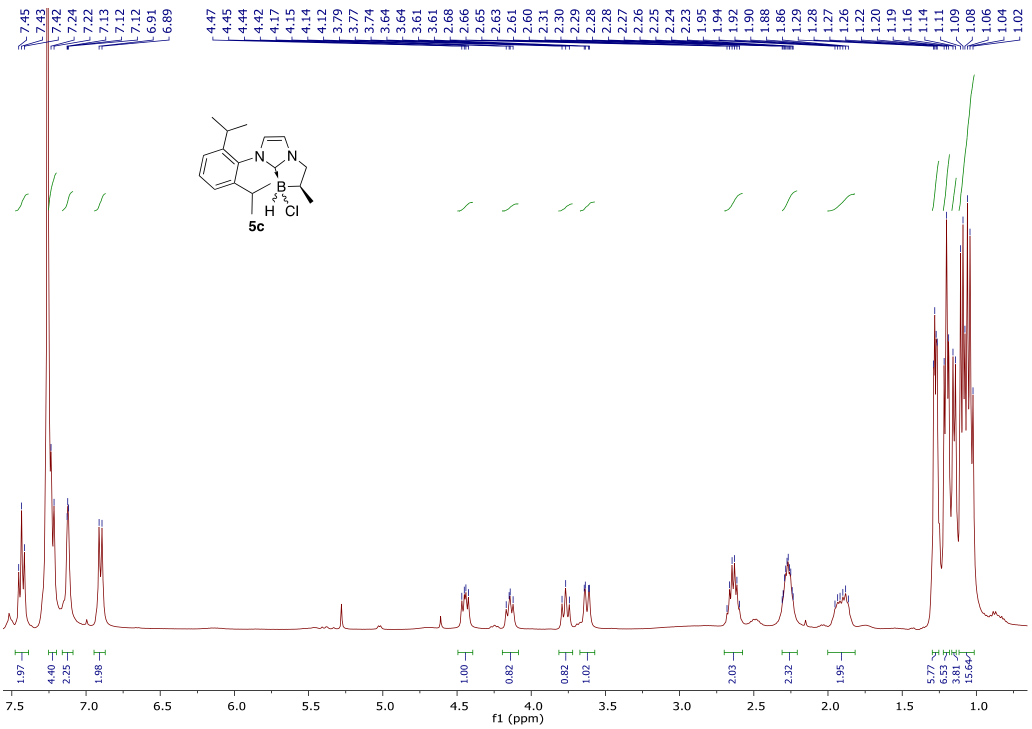
7.45 7.43 7.42 7.24 7.22 7.13 7.12 6.91 6.89
4.47 4.45 4.44 4.42 4.17 4.15 4.14 4.12 3.79 3.77 3.74 3.64 3.64 3.61 3.61 2.68 2.66 2.65 2.63 2.61 2.60 2.31 2.30 2.29 2.28 2.28 2.27 2.26 2.25 2.24 2.23 1.95 1.94 1.92 1.90 1.88 1.86 1.29 1.28 1.27 1.26 1.22 1.20 1.19 1.16 1.14 1.11 1.09 1.08 1.06 1.04 1.02
N
B
H
Cl
5c
1.97 4.40 2.25 1.98 1.00 0.82 0.82 1.02 2.03 2.32 1.95 5.77 6.53 3.81 15.64
f1 (ppm)

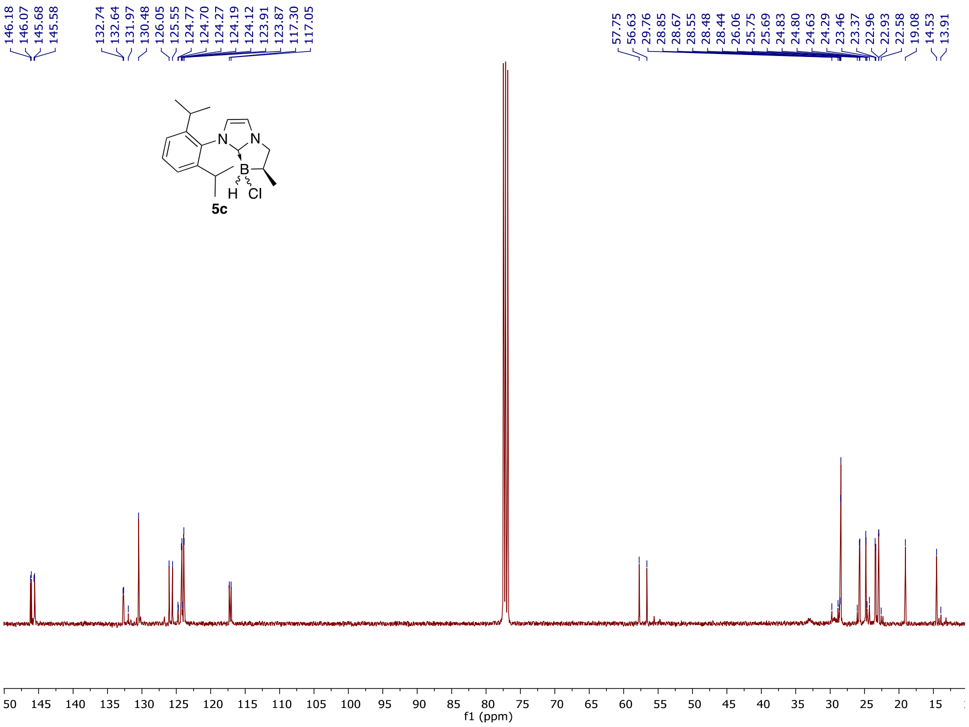
146.18 146.07 145.68 145.58
132.74 132.64 131.97 130.48 126.05 125.55 124.77 124.70 124.27 124.19 124.12 123.91 123.87 117.30 117.05
57.75 56.63 29.76 28.85 28.67 28.55 28.48 28.44 26.06 25.75 25.69 24.83 24.80 24.63 24.29 23.46 23.37 22.96 22.93 22.58 19.08 14.53 13.91
N
B
H
Cl
5c
f1 (ppm)

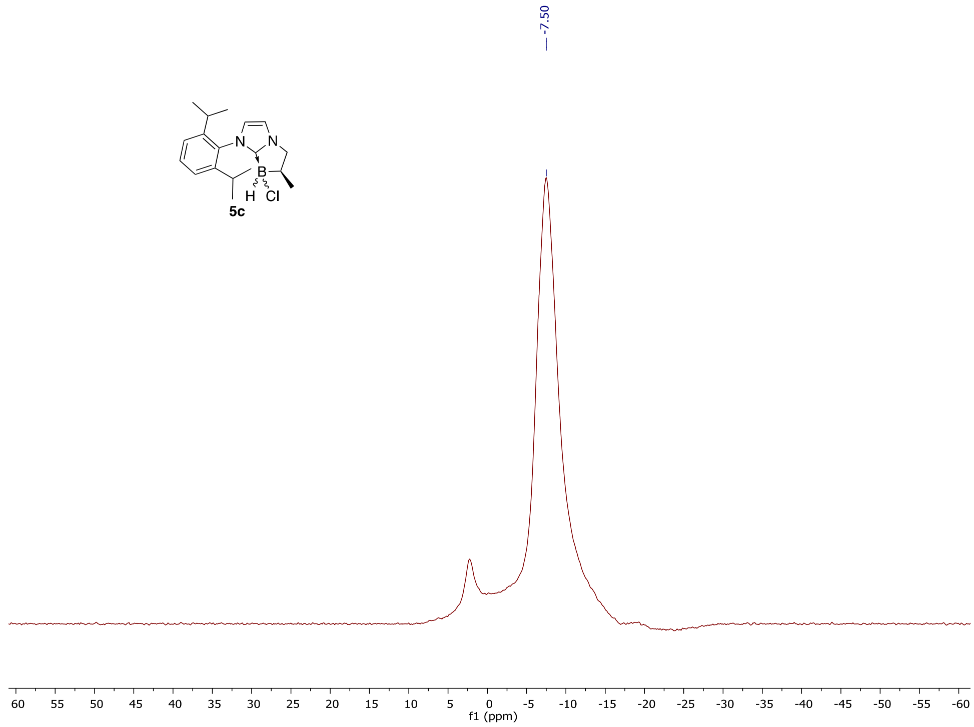
-7.50
N
N
B
H
Cl
5c
60 55 50 45 40 35 30 25 20 15 10 5 0 -5 -10 -15 -20 -25 -30 -35 -40 -45 -50 -55 -60
f1 (ppm)

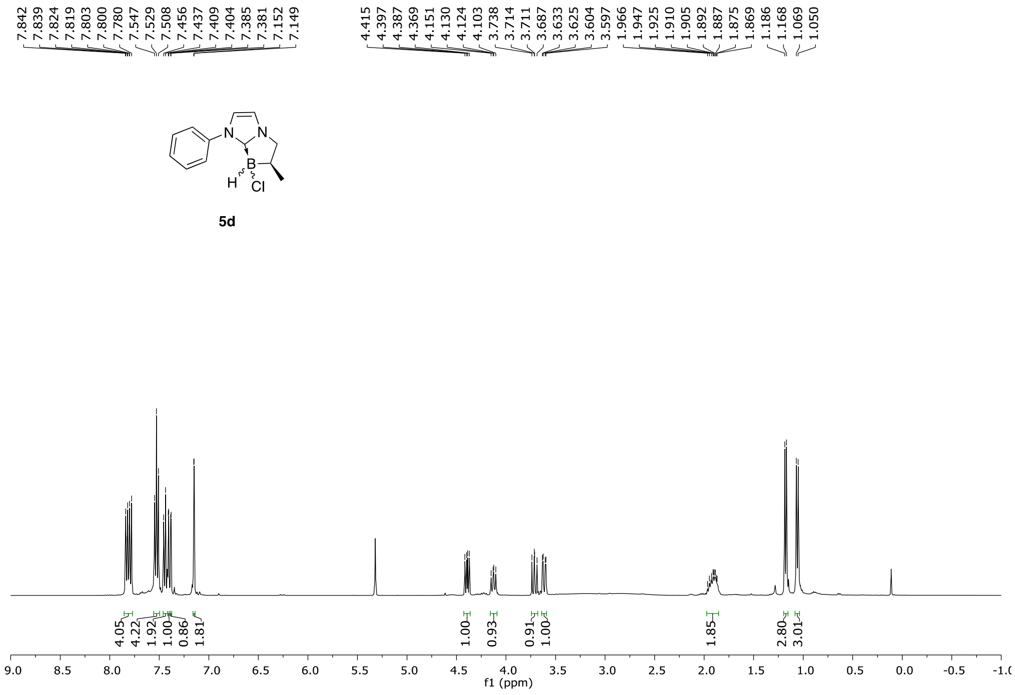
7.842 7.839 7.824 7.819 7.803 7.800 7.780 7.547 7.529 7.508 7.456 7.437 7.409 7.404 7.385 7.381 7.152 7.149
4.415 4.397 4.387 4.369 4.151 4.130 4.124 4.103 3.738 3.714 3.711 3.687 3.633 3.625 3.604 3.597 1.966 1.947 1.925 1.910 1.905 1.892 1.887 1.875 1.869 1.186 1.168 1.069 1.050
N
N
B
H
Cl
5d
4.05 4.22 1.92 1.00 0.86 1.81
1.00 0.93 0.91 1.00
1.85
2.80 3.01
9.0 8.5 8.0 7.5 7.0 6.5 6.0 5.5 5.0 4.5 4.0 3.5 3.0 2.5 2.0 1.5 1.0 0.5 0.0 -0.5 -1.0
f1 (ppm)

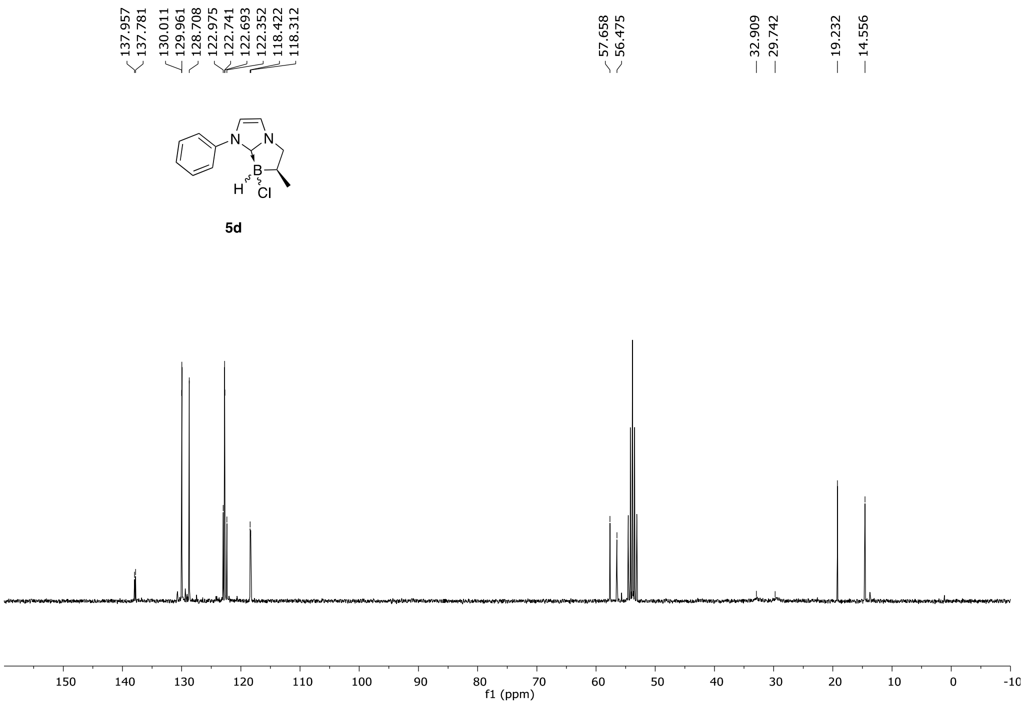
137.957
137.781
130.011
129.961
128.708
122.975
122.741
122.693
122.352
118.422
118.312
57.658
56.475
32.909
29.742
19.232
14.556
N
N
B
H
Cl
5d
150 140 130 120 110 100 90 80 70 60 50 40 30 20 10 0 -10
f1 (ppm)

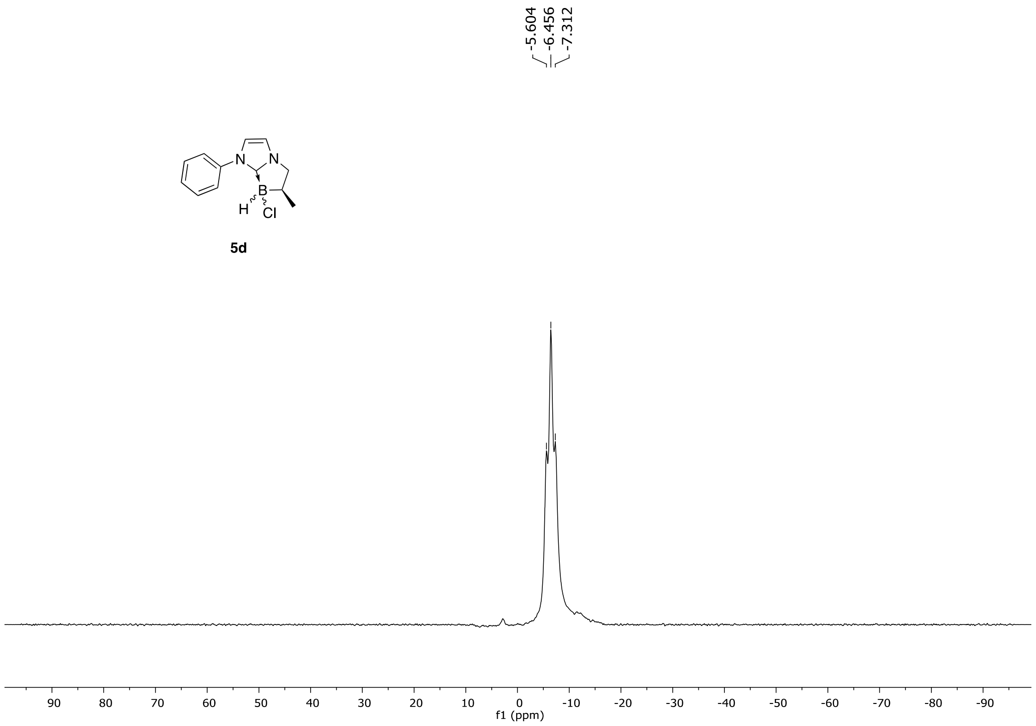
-5.604
-6.456
-7.312
N
N
B
H
Cl
5d
90 80 70 60 50 40 30 20 10 0 -10 -20 -30 -40 -50 -60 -70 -80 -90
f1 (ppm)

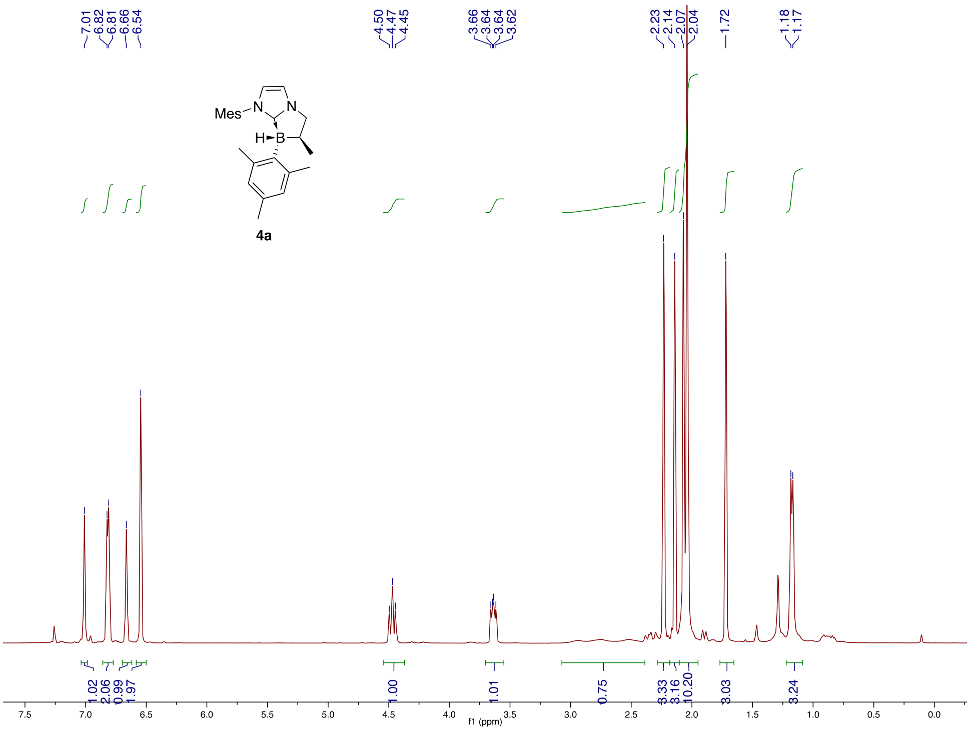
7.01
6.82
6.81
6.66
6.54
4.50
4.47
4.45
3.66
3.64
3.64
3.62
2.23
2.14
2.07
2.04
1.72
1.18
1.17
Mes
N
N
H
B
4a
1.02
2.06
0.99
1.97
1.00
1.01
0.75
3.33
3.16
10.20
3.03
3.24
f1 (ppm)

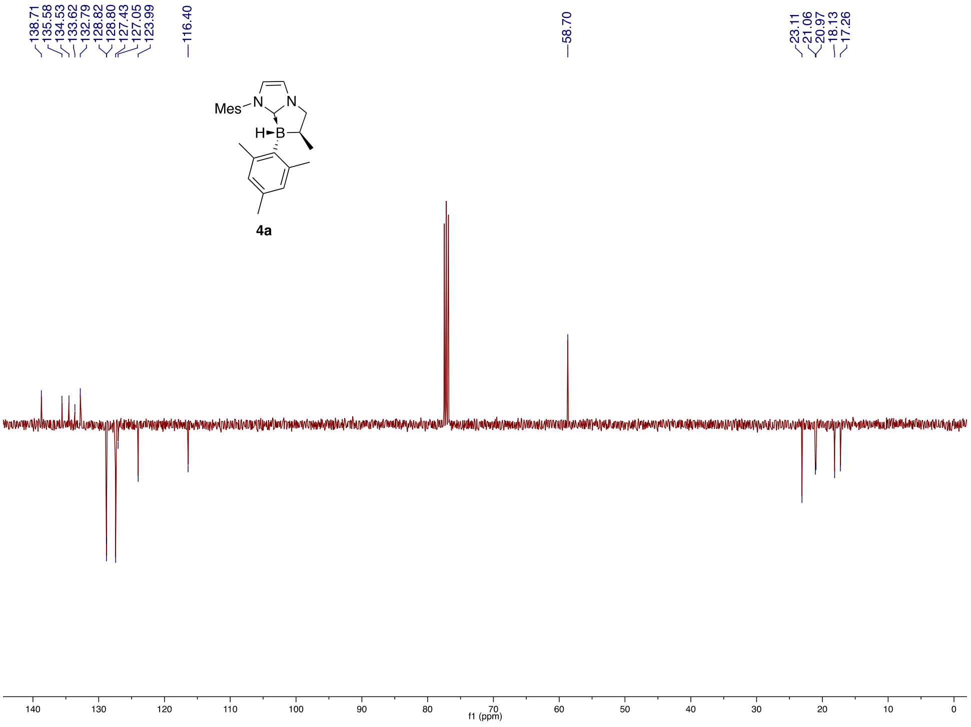
138.71
135.58
134.53
133.62
132.79
128.82
128.80
127.43
127.05
123.99
116.40
58.70
23.11
21.06
20.97
18.13
17.26
Mes
N
N
H
B
4a
f1 (ppm)

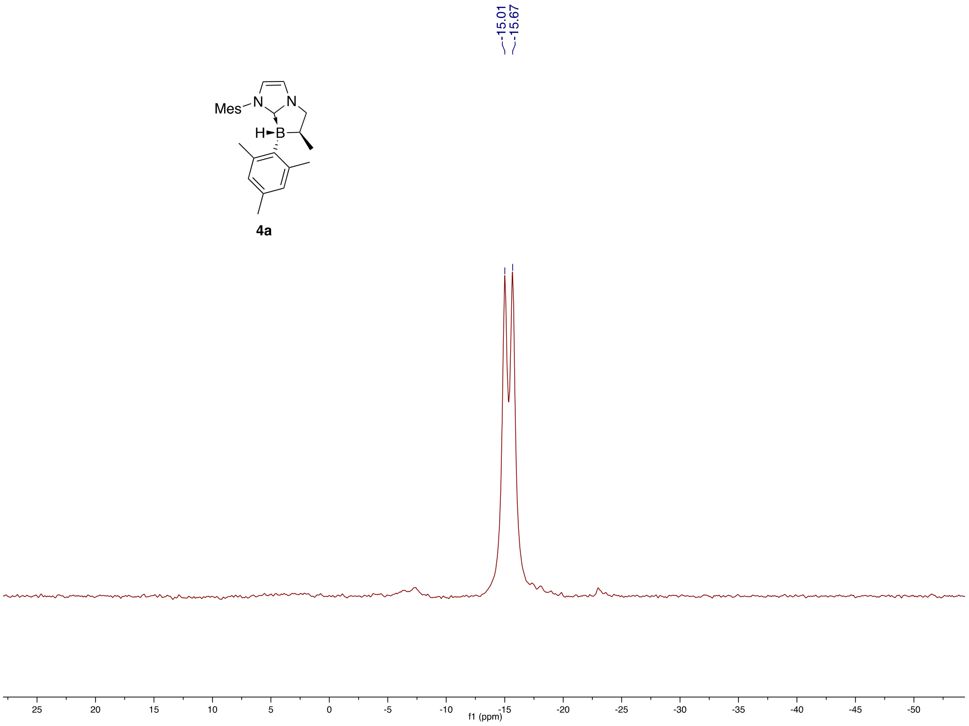
-15.01
-15.67
Mes
N
N
H
B
4a
f1 (ppm)

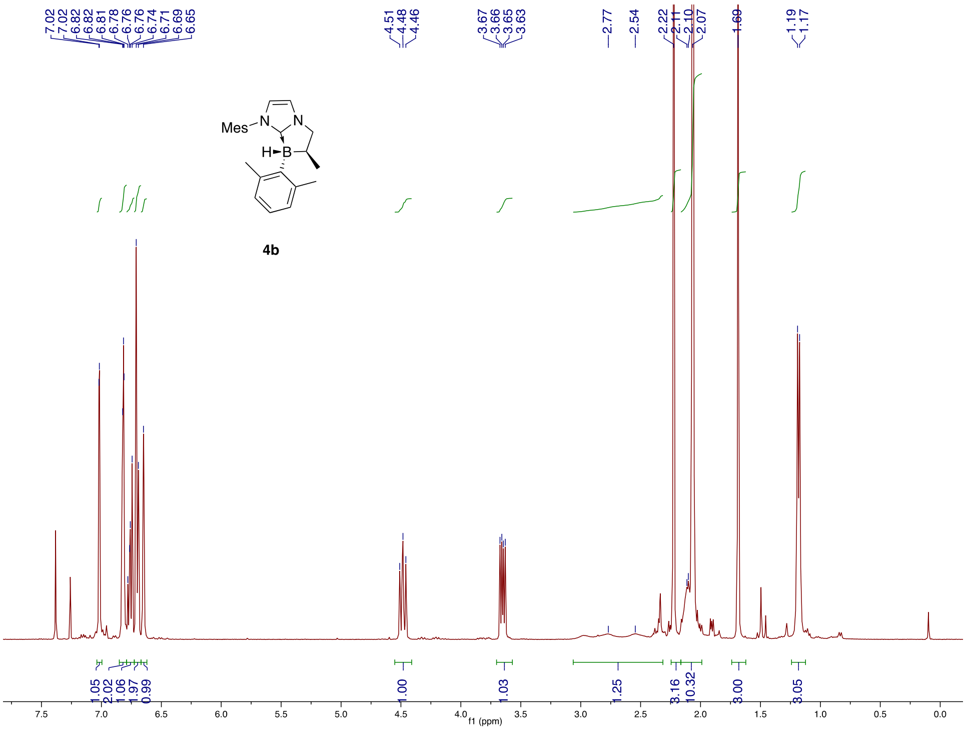
7.02
7.02
6.82
6.81
6.78
6.76
6.76
6.74
6.71
6.69
6.65
4.51
4.48
4.46
3.67
3.66
3.65
3.63
2.77
2.54
2.22
2.11
2.10
2.07
1.69
1.19
1.17
Mes
N
N
H
B
4b
1.05
2.02
1.06
1.97
0.99
1.00
1.03
1.25
3.16
10.32
3.00
3.05
f1 (ppm)

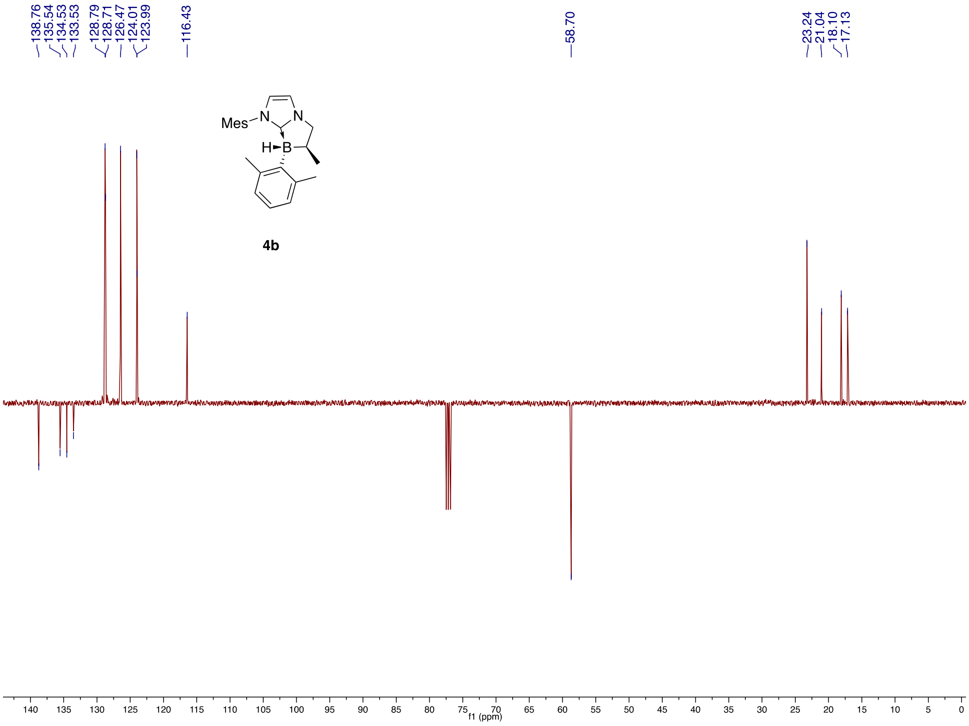
138.76
135.54
134.53
133.53
128.79
128.71
126.47
124.01
123.99
116.43
58.70
23.24
21.04
18.10
17.13
Mes
N
N
H
B
4b
f1 (ppm)

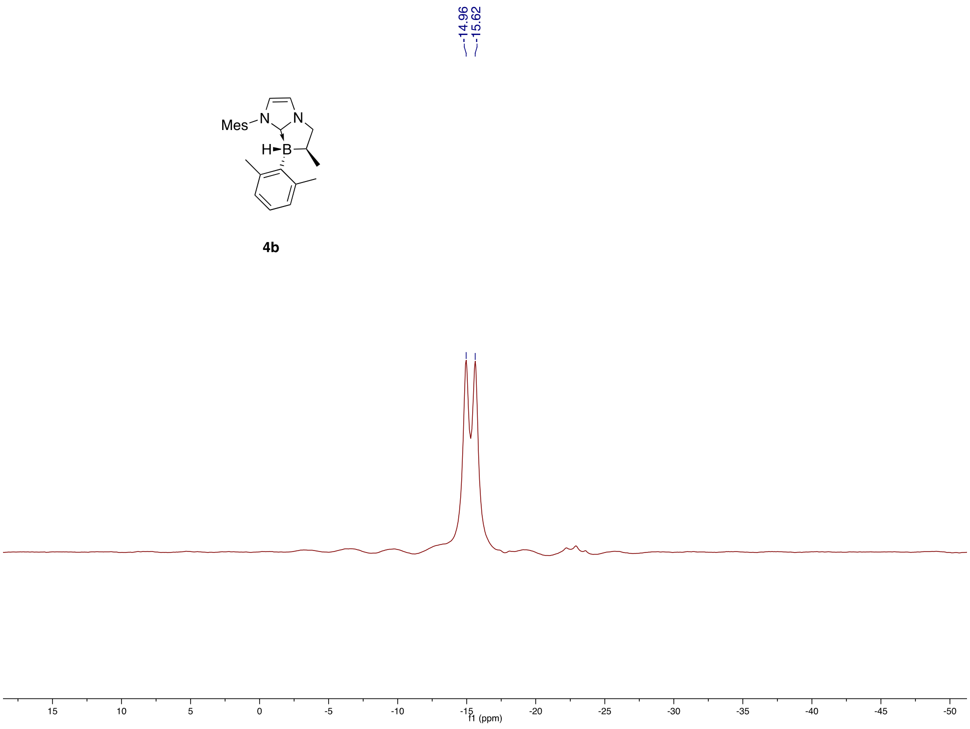
-14.96
-15.62
Mes
N
N
H
B
4b
f1 (ppm)

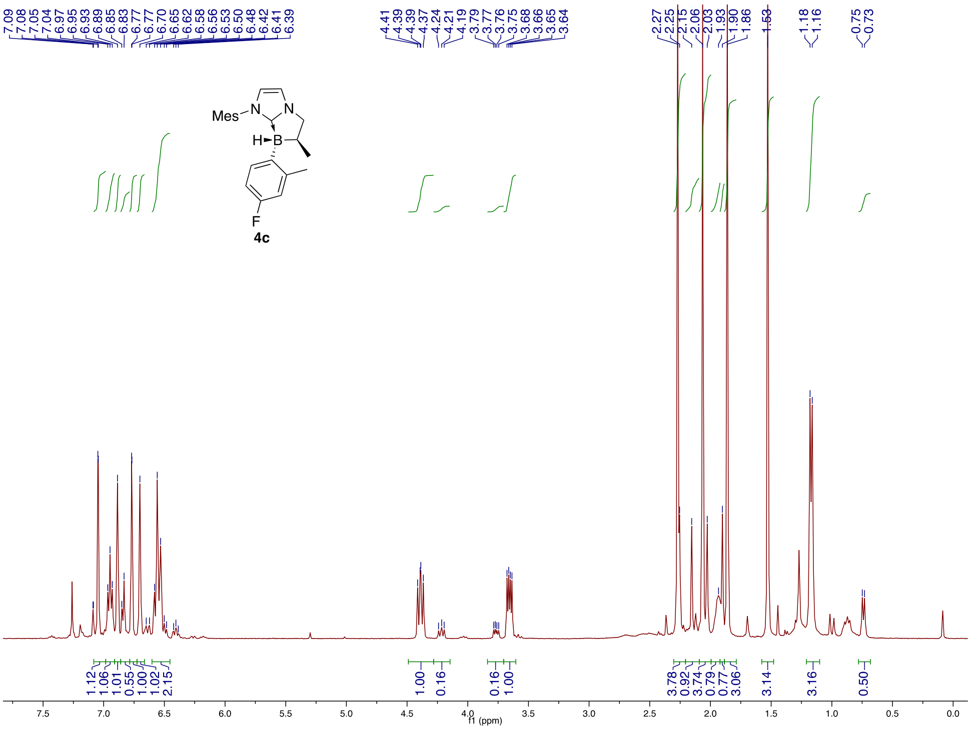
7.09 7.08 7.05 7.04 6.97 6.95 6.93 6.89 6.85 6.83 6.77 6.77 6.70 6.65 6.62 6.58 6.56 6.53 6.50 6.48 6.42 6.41 6.39
4.41 4.39 4.39 4.37 4.24 4.21 4.19 3.79 3.77 3.76 3.75 3.68 3.66 3.65 3.64
2.27 2.25 2.15 2.06 2.03 1.93 1.90 1.86 1.53
1.18 1.16
0.75 0.73
Mes
N
N
H
B
F
4c
1.12 1.06 1.01 0.55 1.00 1.02 2.15
1.00 0.16
0.16 1.00
3.78 0.92 3.74 0.79 0.77 3.06
3.14
3.16
0.50
f1 (ppm)

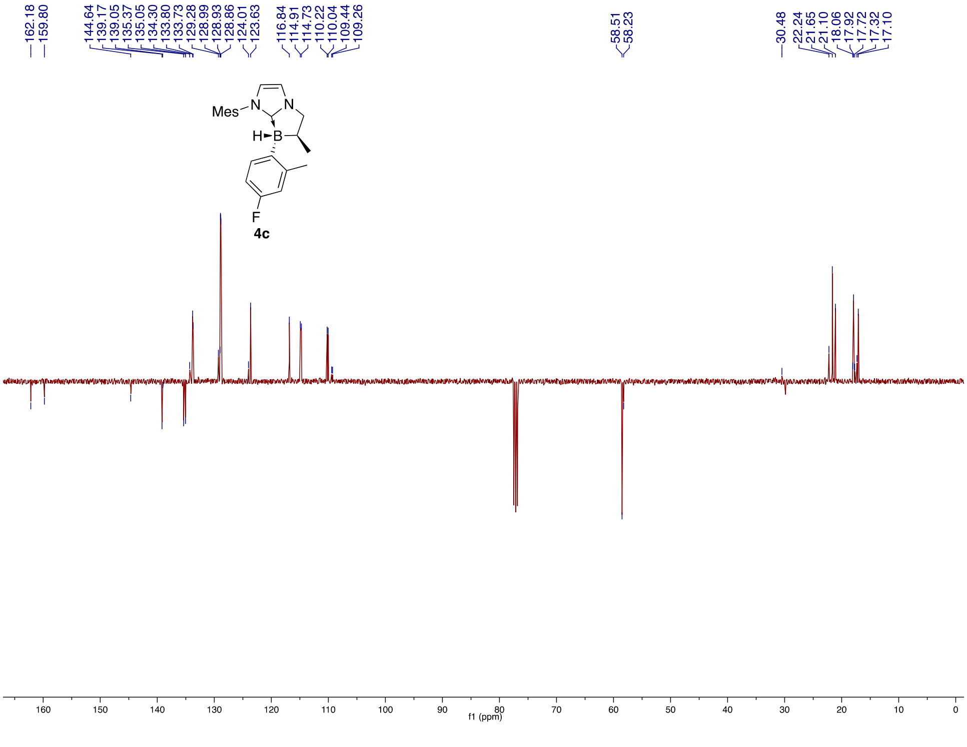
162.18 159.80
144.64 139.17 139.05 135.37 135.05 134.30 133.80 133.73 129.28 128.99 128.93 128.86 124.01 123.63
116.84 114.91 114.73 110.22 110.04 109.44 109.26
58.51 58.23
30.48 22.24 21.65 21.10 18.06 17.92 17.72 17.32 17.10
Mes
N
N
H
B
F
4c
f1 (ppm)

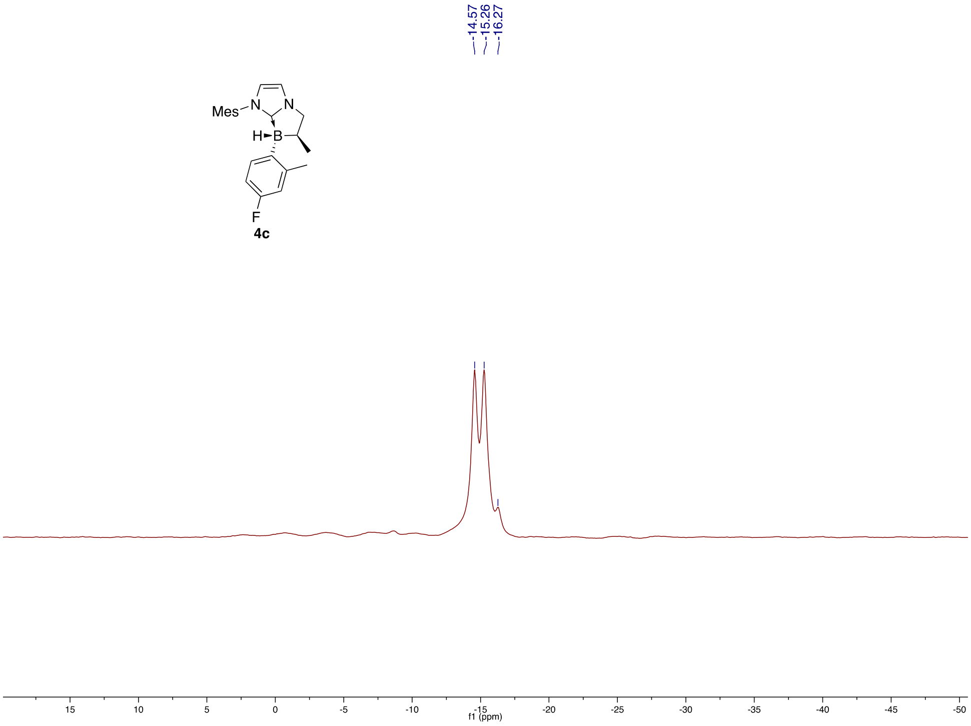

-14.57
-15.26
-16.27
Mes
N
N
H
B
F
4c
15 10 5 0 -5 -10 -15 -20 -25 -30 -35 -40 -45 -50
f1 (ppm)

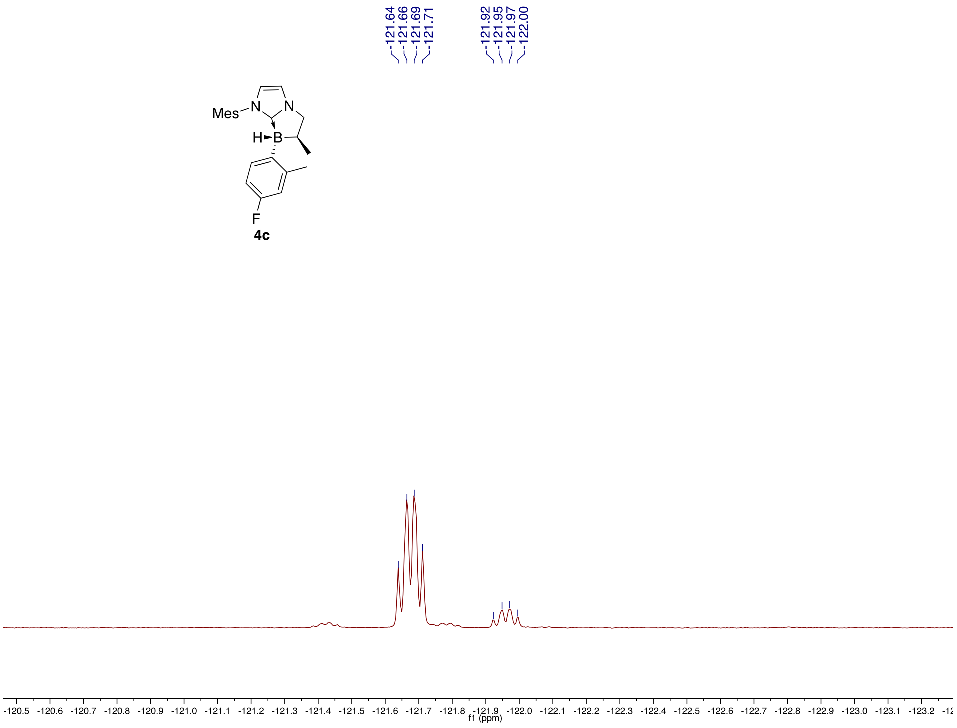

-121.64
-121.66
-121.69
-121.71
-121.92
-121.95
-121.97
-122.00
Mes
N
N
H
B
F
4c
-120.5 -120.6 -120.7 -120.8 -120.9 -121.0 -121.1 -121.2 -121.3 -121.4 -121.5 -121.6 -121.7 -121.8 -121.9 -122.0 -122.1 -122.2 -122.3 -122.4 -122.5 -122.6 -122.7 -122.8 -122.9 -123.0 -123.1 -123.2
f1 (ppm)

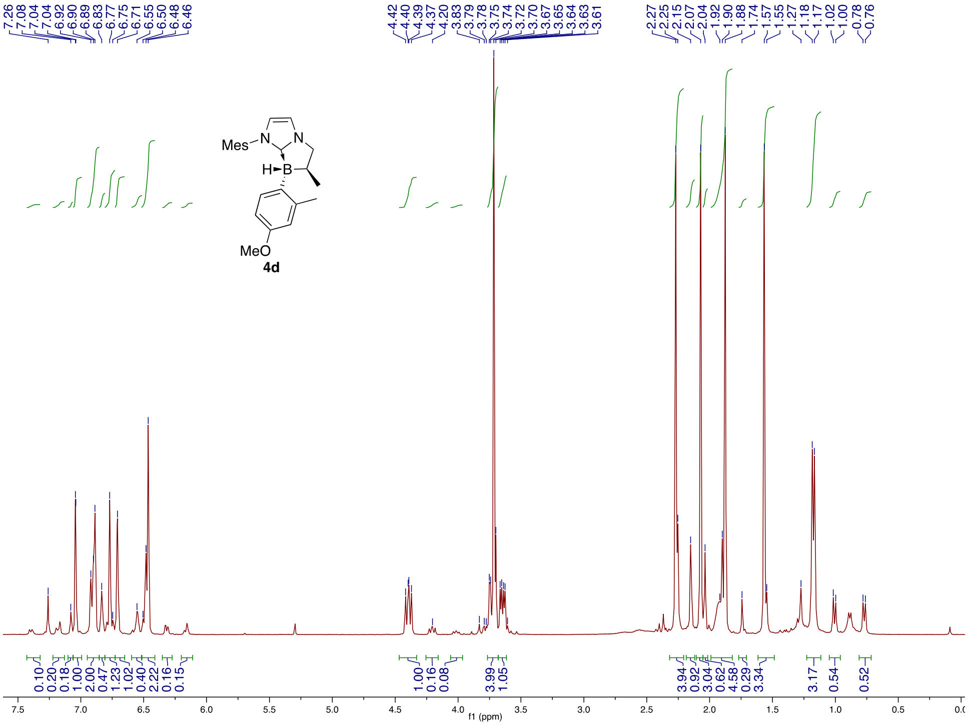

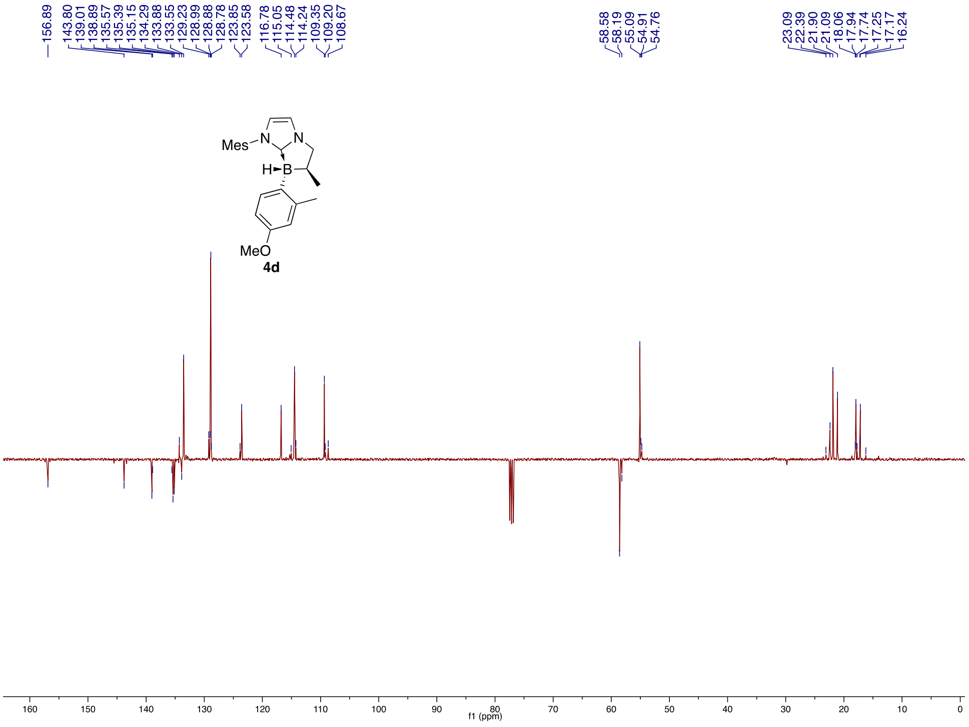

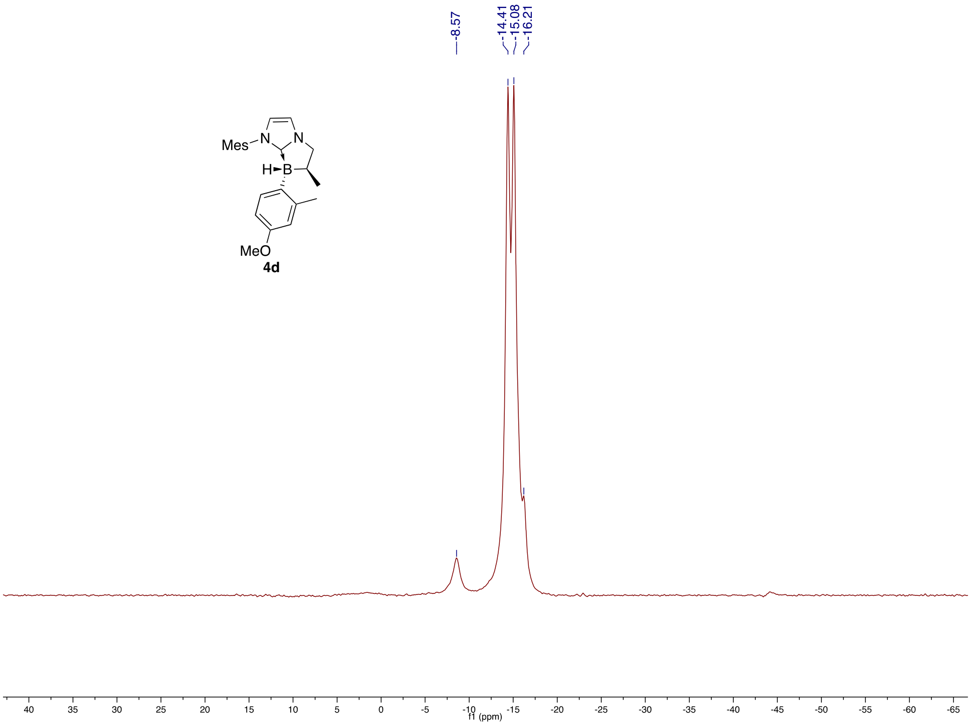

-8.57
-14.41
-15.08
-16.21
Mes
N
N
H
B
MeO
4d
f1 (ppm)

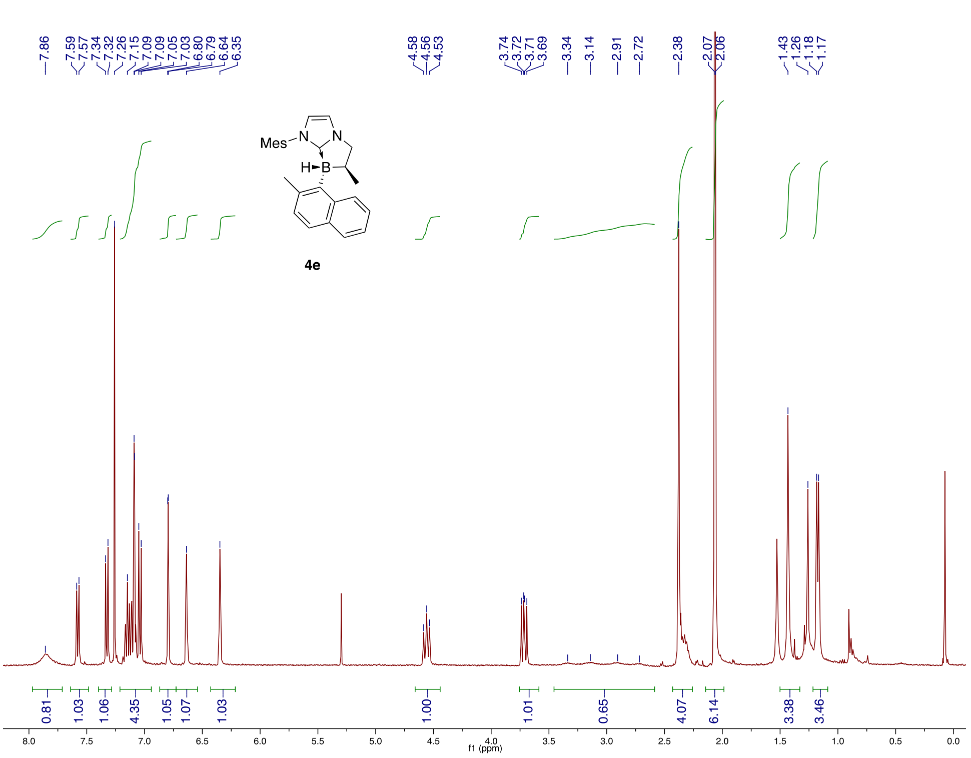

7.86
7.59
7.57
7.34
7.32
7.26
7.15
7.09
7.09
7.05
7.03
6.80
6.79
6.64
6.35
4.58
4.56
4.53
3.74
3.72
3.71
3.69
3.34
3.14
2.91
2.72
2.38
2.07
2.06
1.43
1.26
1.18
1.17
Mes
N
N
H
B
4e
0.81
1.03
1.06
4.35
1.05
1.07
1.03
1.00
1.01
0.65
4.07
6.14
3.38
3.46
f1 (ppm)

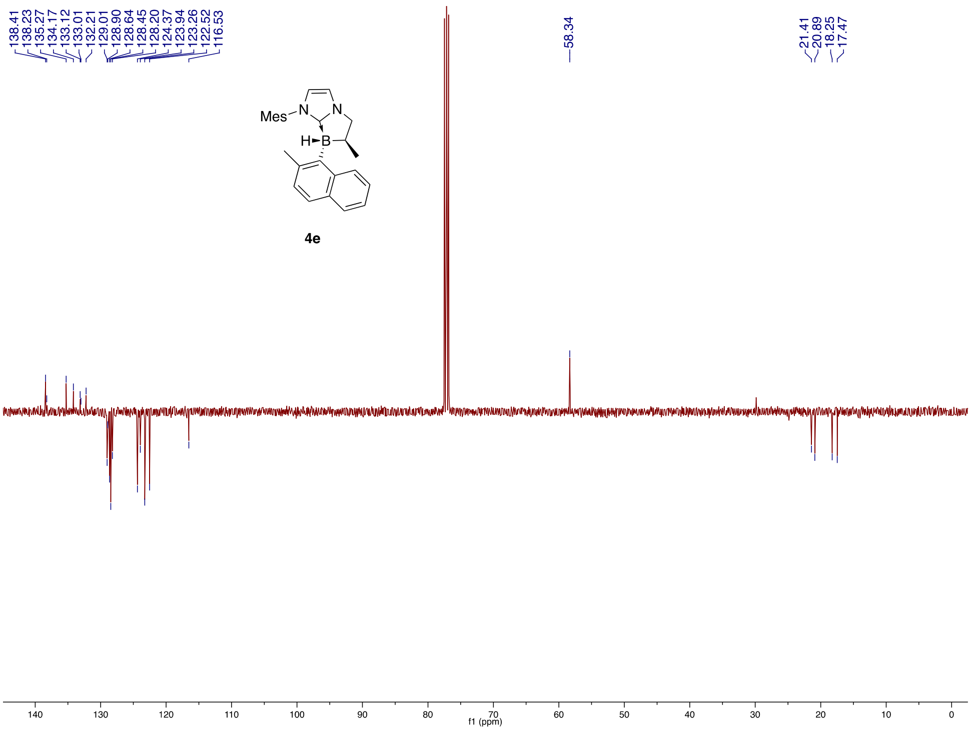
138.41
138.23
135.27
134.17
133.12
133.01
132.21
129.01
128.90
128.64
128.45
128.20
124.37
123.94
123.26
122.52
116.53
58.34
21.41
20.89
18.25
17.47
Mes
N
N
H
B
4e
f1 (ppm)

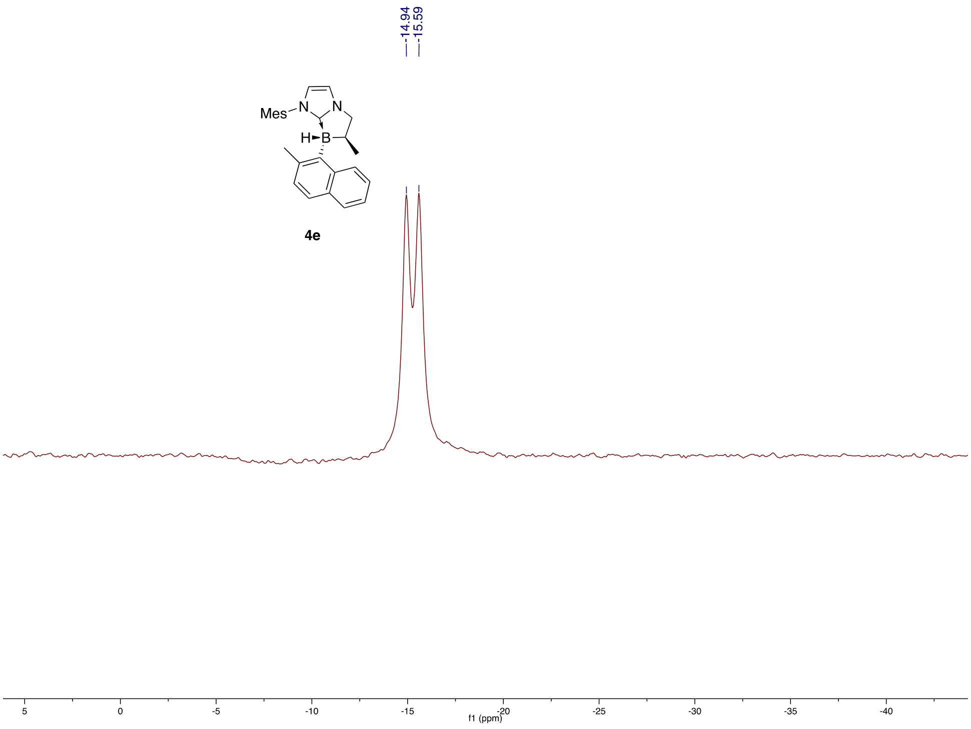
-14.94
-15.59
Mes
N
N
H
B
4e
f1 (ppm)

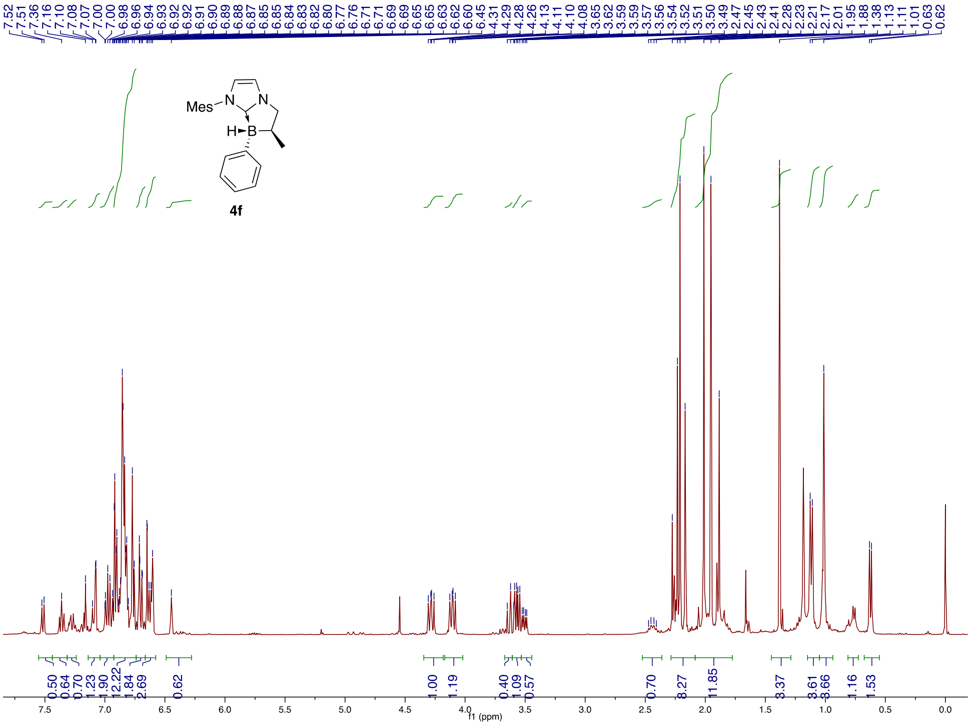

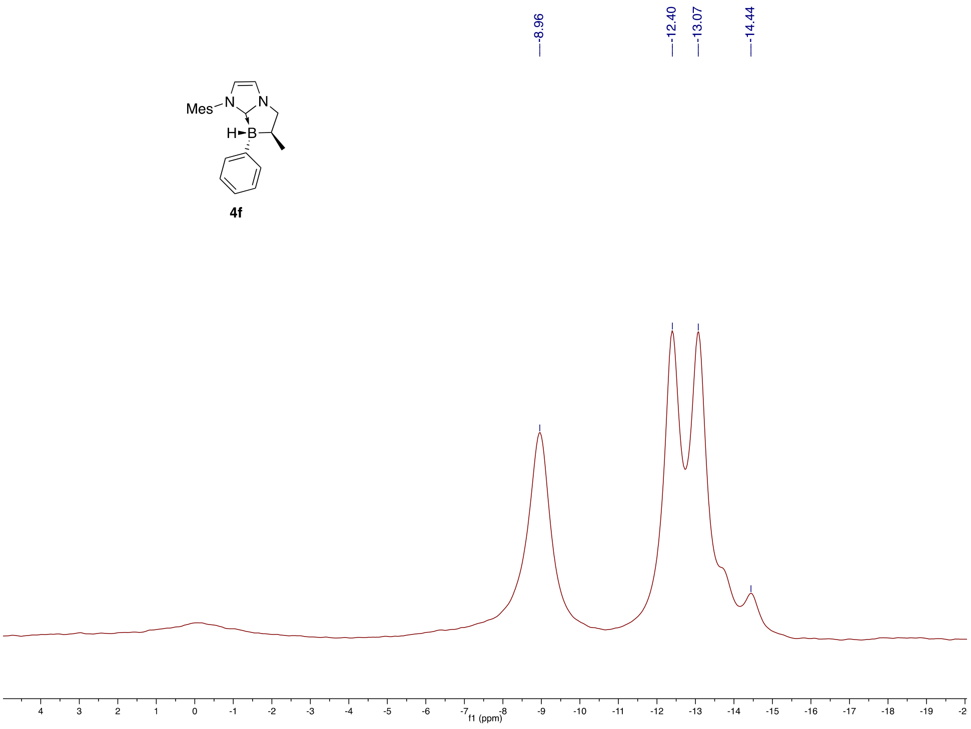

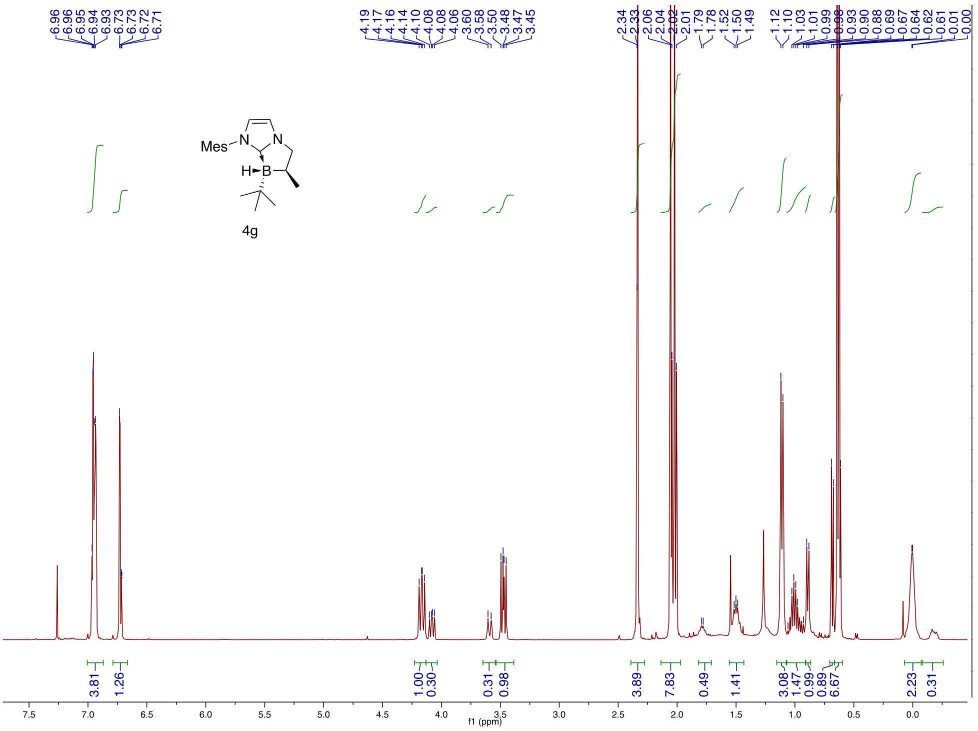

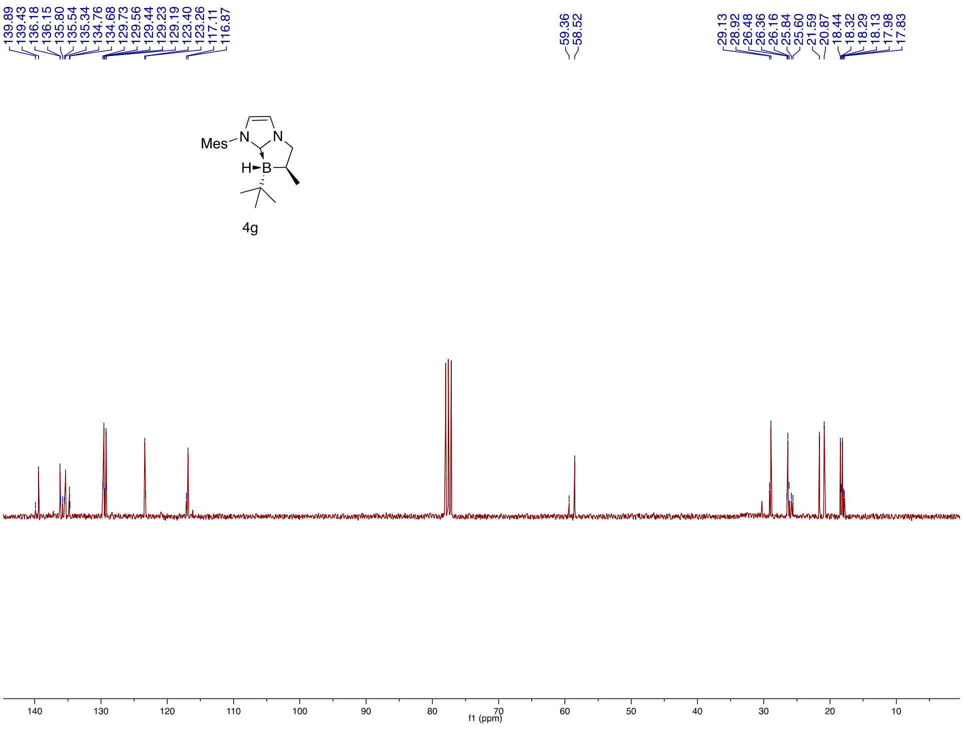

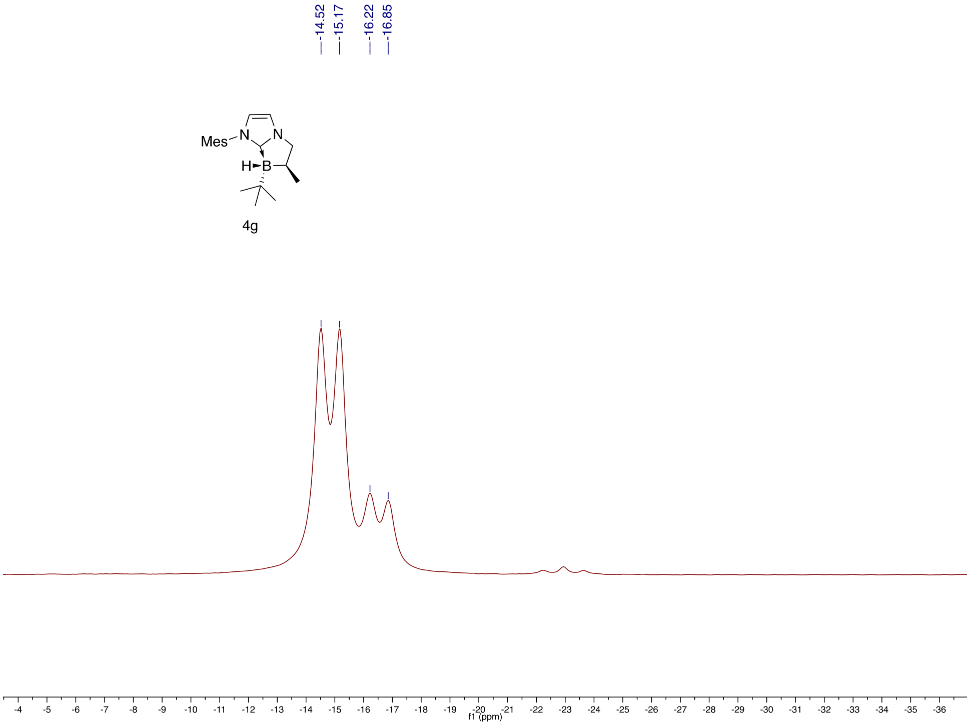

-14.52
-15.17
-16.22
-16.85
Mes
N
N
H
B
4g
f1 (ppm)

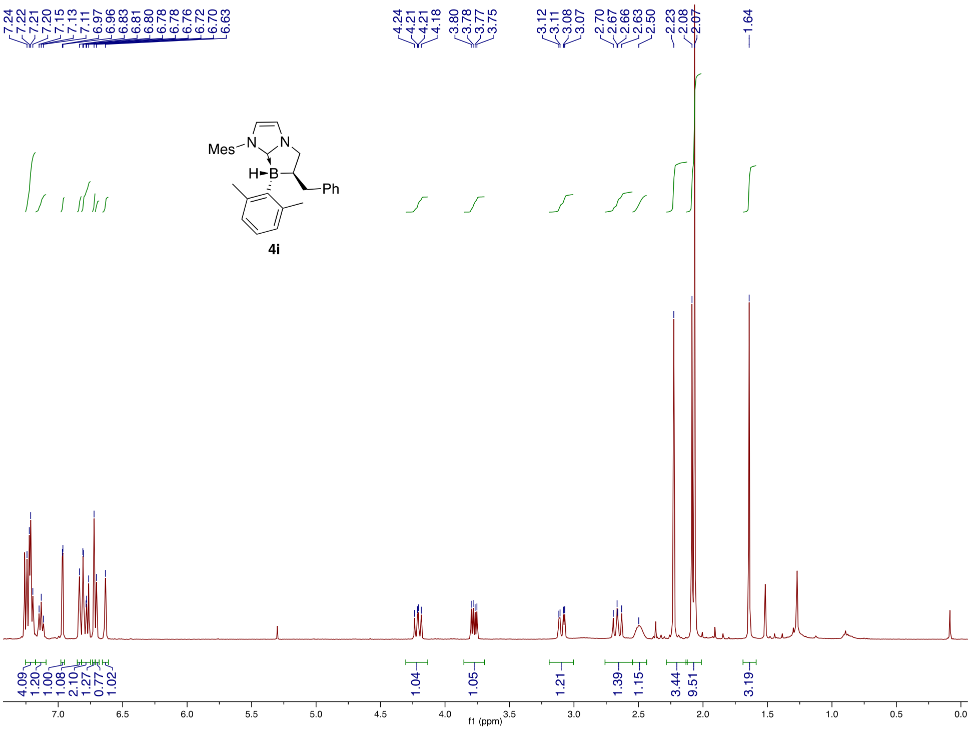

7.24
7.22
7.21
7.20
7.15
7.13
7.11
6.97
6.96
6.83
6.81
6.80
6.78
6.78
6.76
6.72
6.70
6.63
4.24
4.21
4.21
4.18
3.80
3.78
3.77
3.75
3.12
3.11
3.08
3.07
2.70
2.67
2.66
2.63
2.50
2.23
2.08
2.07
1.64
Mes
N
N
H
B
Ph
4i
4.09
1.20
1.00
1.08
2.10
1.27
0.77
1.02
1.04
1.05
1.21
1.39
1.15
3.44
9.51
3.19
f1 (ppm)

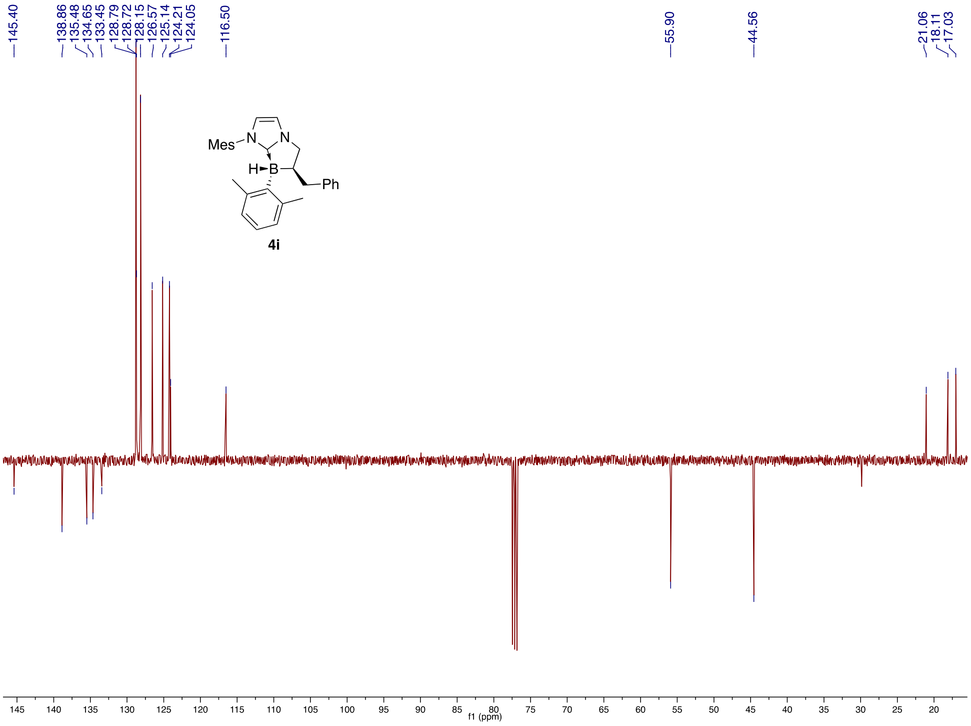

145.40
138.86
135.48
134.65
133.45
128.79
128.72
128.15
126.57
125.14
124.21
124.05
116.50
55.90
44.56
21.06
18.11
17.03
Mes
N
N
H
B
Ph
4i
f1 (ppm)

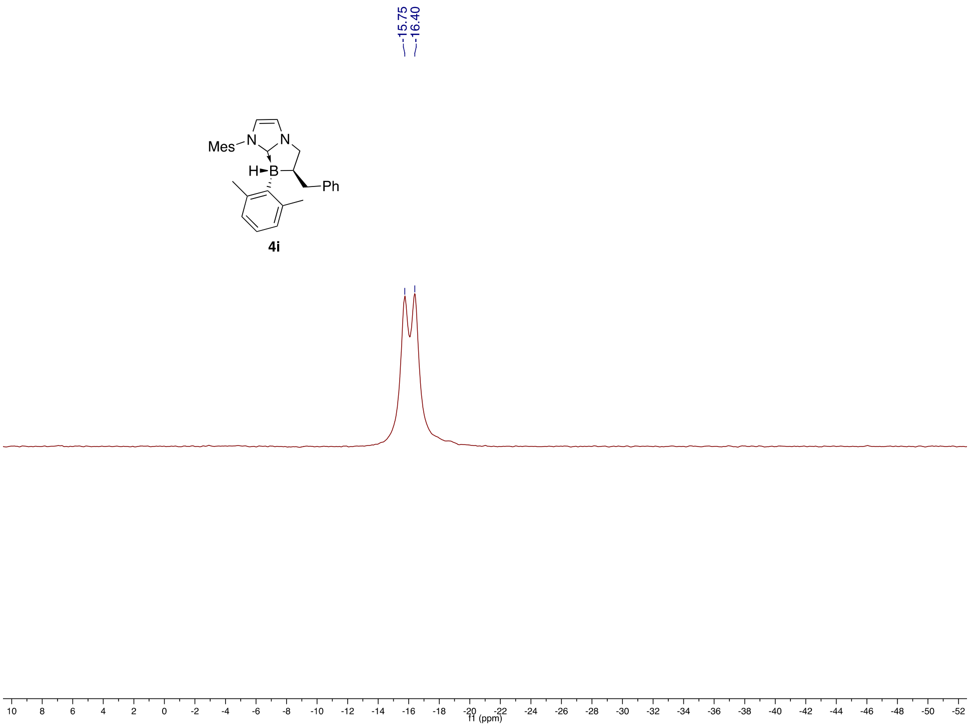

-15.75
-16.40
Mes
N
N
H
B
Ph
4i
f1 (ppm)

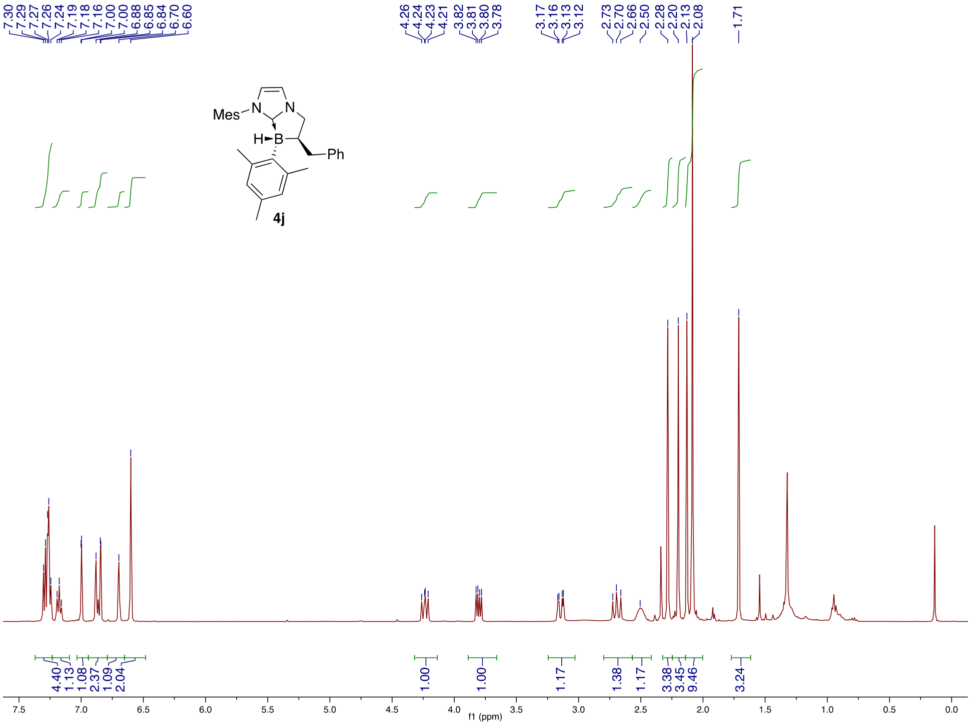
7.30
7.29
7.27
7.26
7.24
7.19
7.18
7.16
7.00
7.00
6.88
6.85
6.84
6.70
6.60
4.26
4.24
4.23
4.21
3.82
3.81
3.80
3.78
3.17
3.16
3.13
3.12
2.73
2.70
2.66
2.50
2.28
2.20
2.13
2.08
1.71
Mes
N
N
H
B
Ph
4j
4.40
1.13
1.08
2.37
1.09
2.04
1.00
1.00
1.17
1.38
1.17
3.38
3.45
9.46
3.24
f1 (ppm)

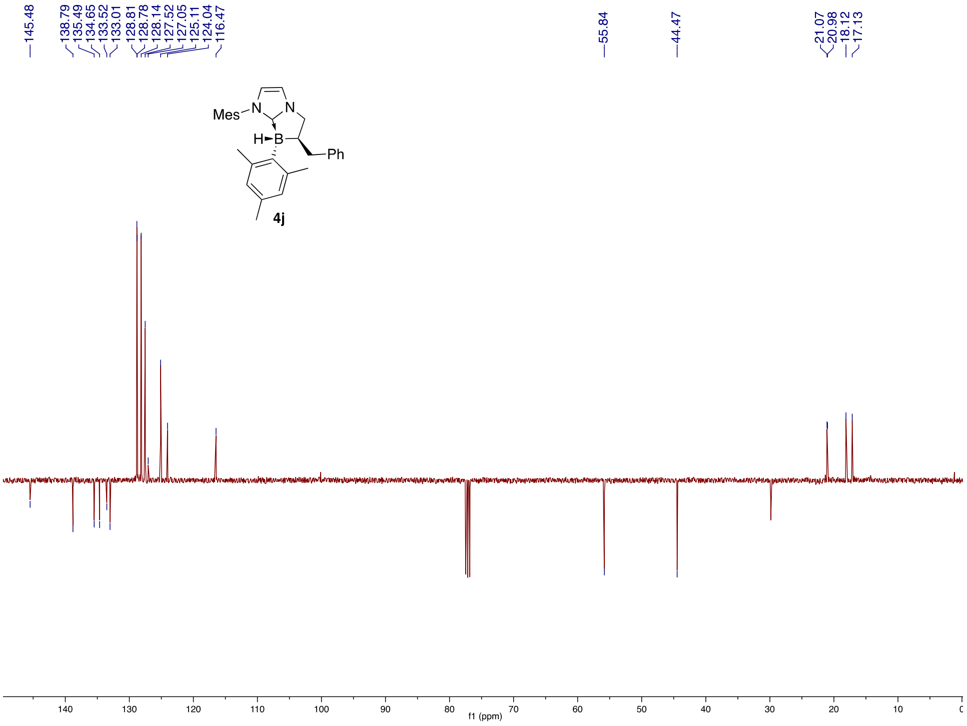
145.48
138.79
135.49
134.65
133.52
133.01
128.81
128.78
128.14
127.52
127.05
125.11
124.04
116.47
55.84
44.47
21.07
20.98
18.12
17.13
Mes
N
N
H
B
Ph
4j
f1 (ppm)

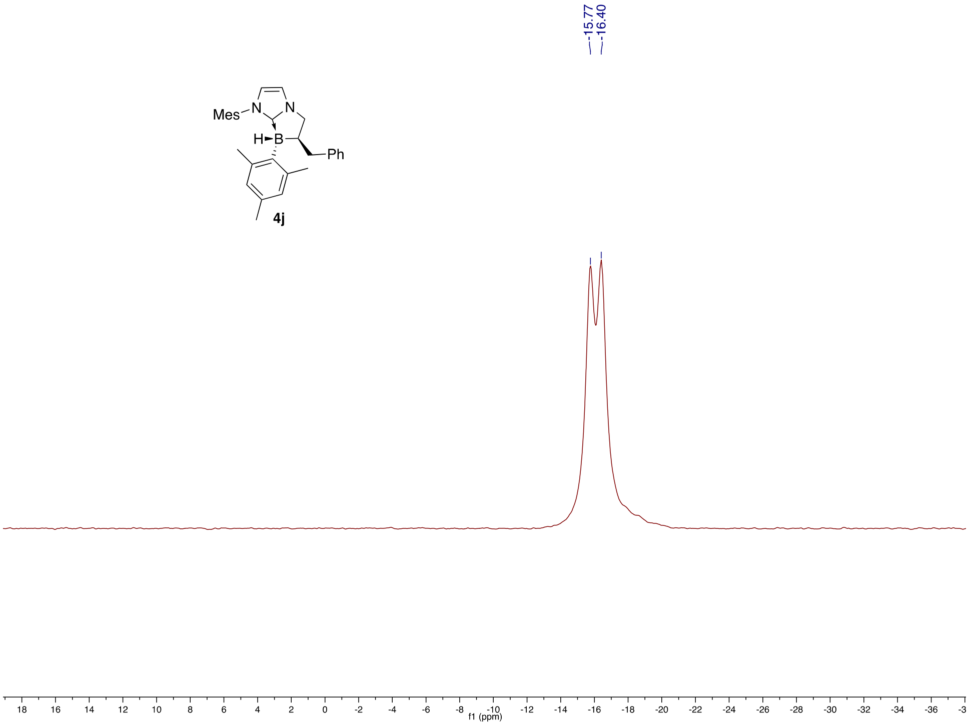
-15.77
-16.40
Mes
N
N
H
B
Ph
4j
f1 (ppm)

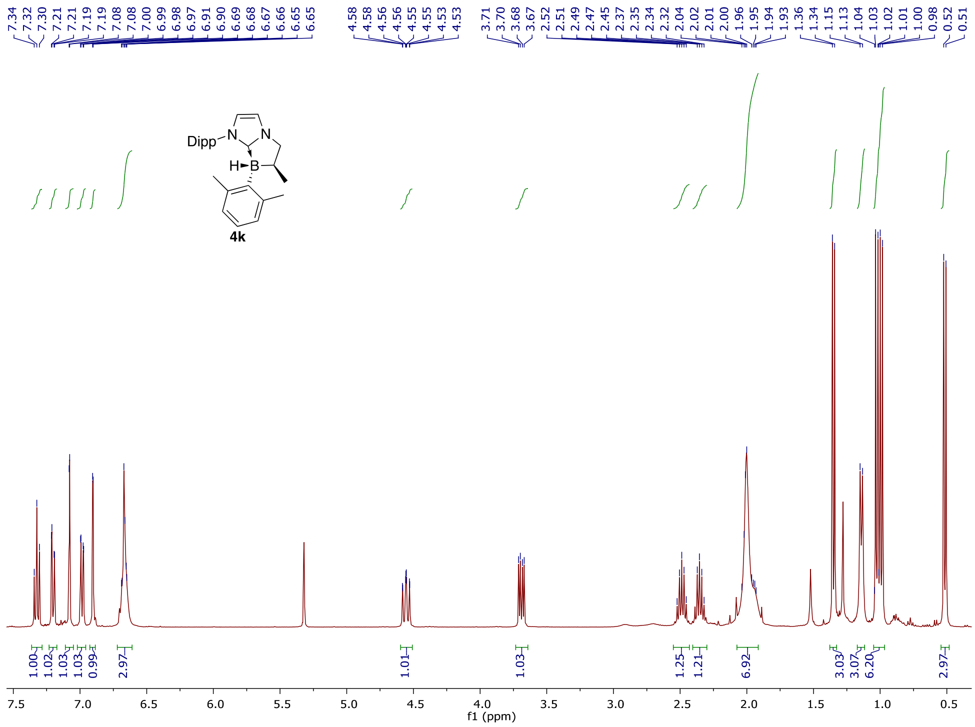
7.34 7.32 7.30 7.21 7.21 7.19 7.19 7.08 7.08 7.00 6.99 6.98 6.97 6.91 6.90 6.69 6.68 6.67 6.66 6.65 6.65
4.58 4.58 4.56 4.56 4.55 4.55 4.53 4.53
3.71 3.70 3.68 3.67
2.52 2.51 2.49 2.47 2.45 2.37 2.35 2.34 2.32 2.04 2.02 2.01 2.00 1.96 1.95 1.94 1.93
1.36 1.34 1.15 1.13 1.04 1.03 1.02 1.01 1.00 0.98 0.52 0.51
Dipp
N
N
H
B
4k
1.00 1.02 1.03 1.03 0.99 2.97 1.01 1.03 1.25 1.21 6.92 3.03 3.07 6.20 2.97
f1 (ppm)

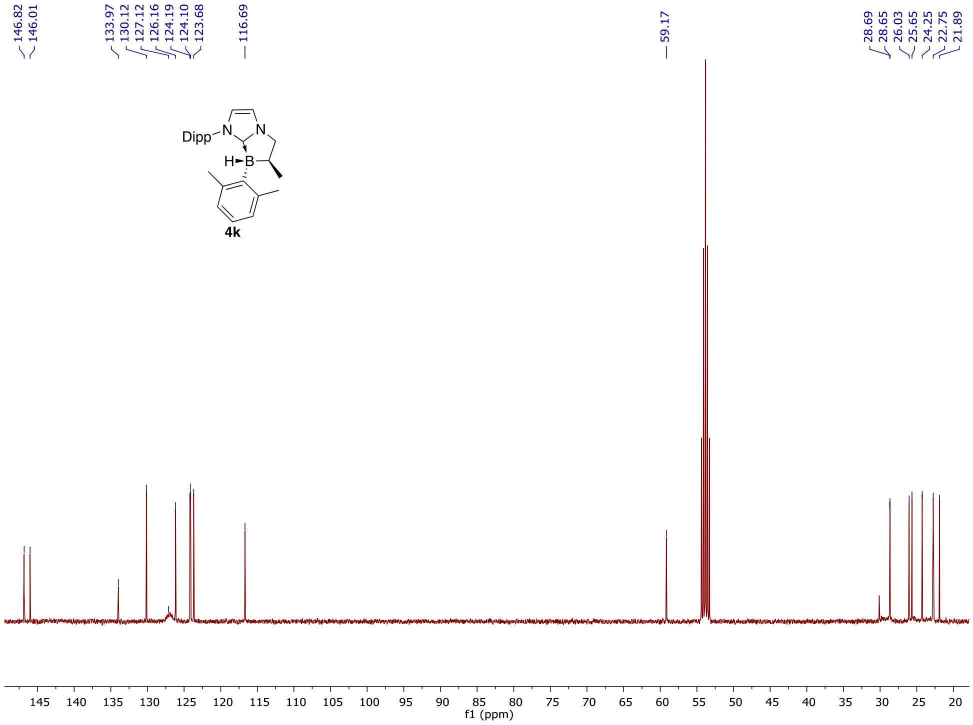
146.82
146.01
133.97
130.12
127.12
126.16
124.19
124.10
123.68
116.69
59.17
28.69
28.65
26.03
25.65
24.25
22.75
21.89
Dipp
N
N
H
B
4k
f1 (ppm)

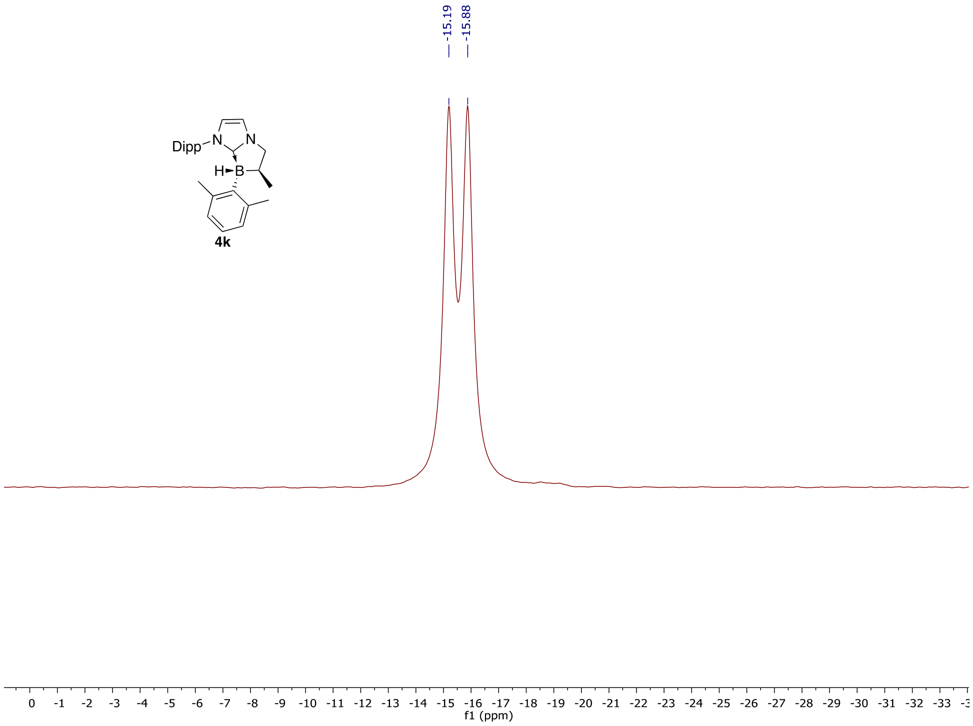
-15.19
-15.88
Dipp
N
N
H
B
4k
f1 (ppm)

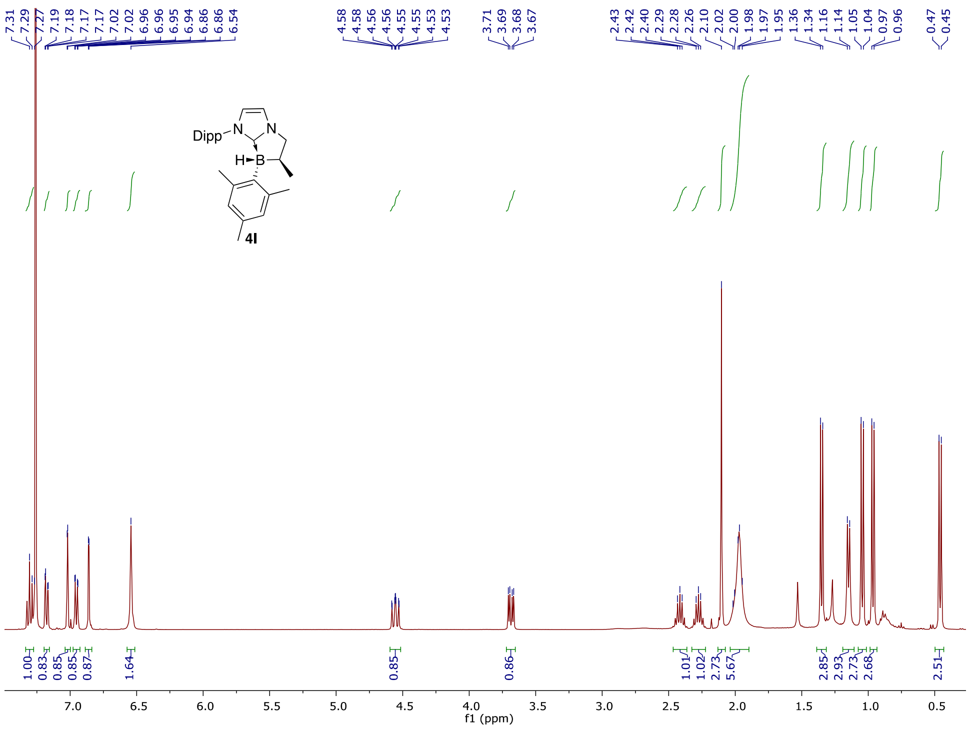
7.31 7.29 7.27 7.19 7.18 7.17 7.17 7.02 7.02 6.96 6.96 6.95 6.94 6.86 6.86 6.54
4.58 4.58 4.56 4.56 4.55 4.55 4.53 4.53
3.71 3.69 3.68 3.67
2.43 2.42 2.40 2.29 2.28 2.26 2.10 2.02 2.00 1.98 1.97 1.95 1.36 1.34 1.16 1.14 1.05 1.04 0.97 0.96
0.47 0.45
Dipp
N
N
H
B
4l
1.00 0.83 0.85 0.85 0.87 1.64 0.85 0.86 1.01 1.02 2.73 5.67 2.85 2.93 2.73 2.68 2.51
f1 (ppm)

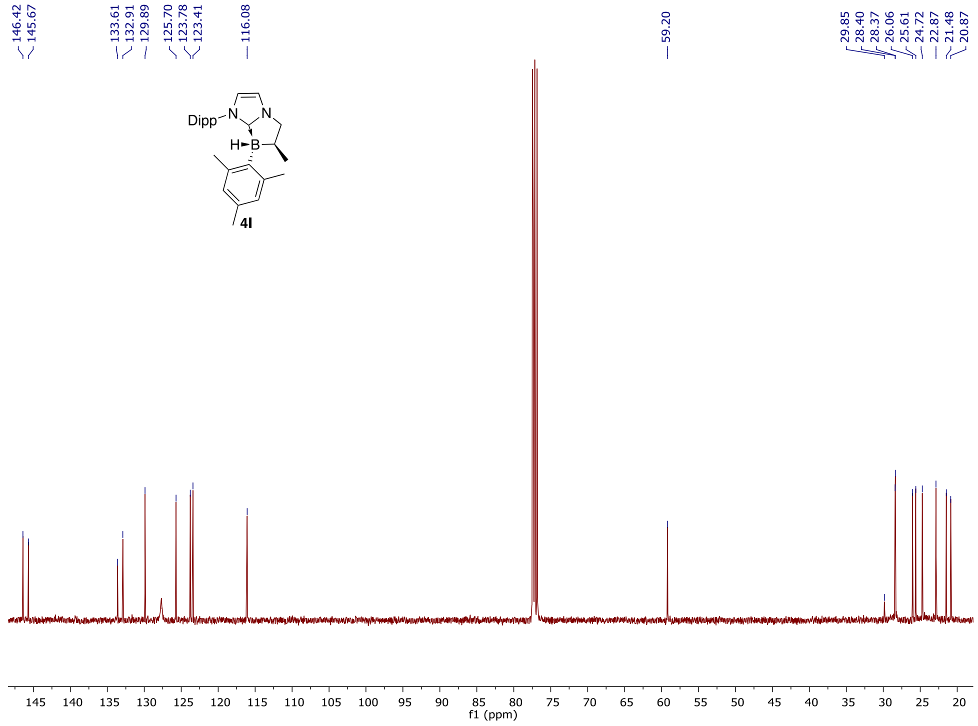
146.42 145.67
133.61 132.91 129.89 125.70 123.78 123.41 116.08
59.20
29.85 28.40 28.37 26.06 25.61 24.72 22.87 21.48 20.87
Dipp
N
N
H
B
4l
f1 (ppm)

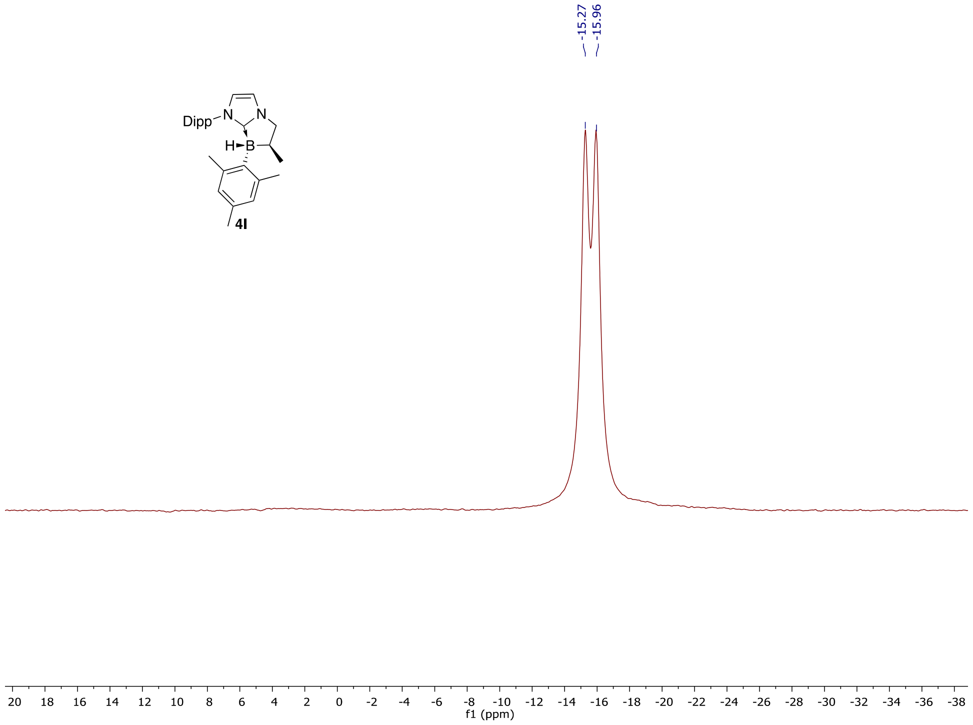

-15.27
-15.96
Dipp
N
N
H
B
4l
20 18 16 14 12 10 8 6 4 2 0 -2 -4 -6 -8 -10 -12 -14 -16 -18 -20 -22 -24 -26 -28 -30 -32 -34 -36 -38
f1 (ppm)

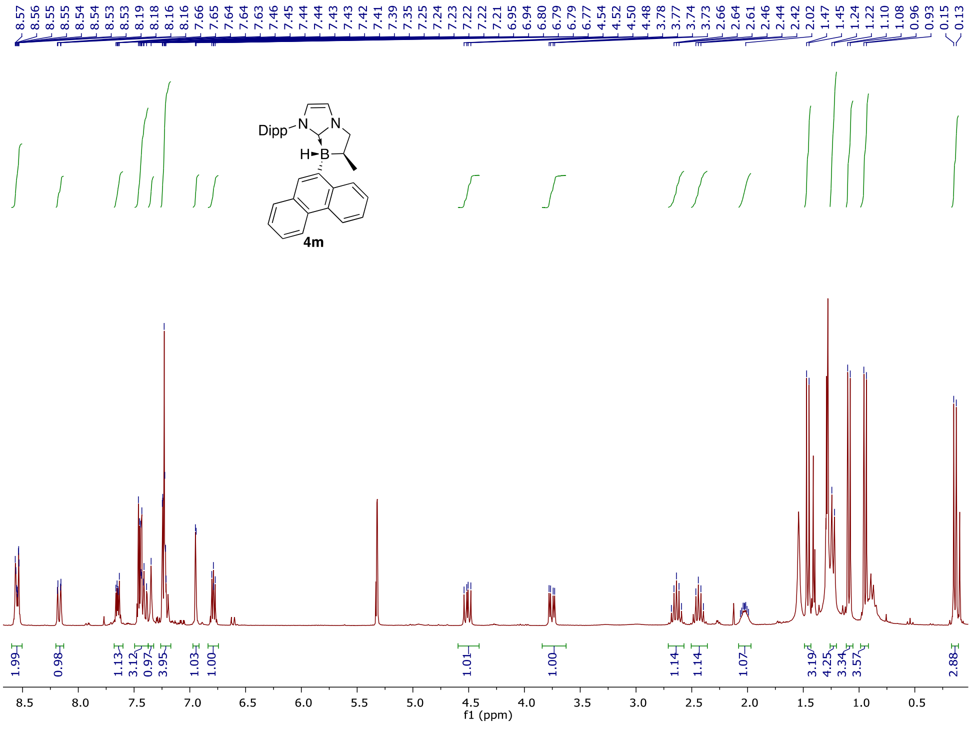

8.57 8.56 8.55 8.55 8.54 8.54 8.53 8.19 8.18 8.16 8.16 7.66 7.65 7.64 7.64 7.63 7.46 7.45 7.44 7.43 7.43 7.42 7.41 7.39 7.35 7.25 7.24 7.23 7.22 7.22 7.21 6.95 6.94 6.80 6.79 6.79 6.77 4.54 4.52 4.50 4.48 3.78 3.77 3.74 3.73 2.66 2.64 2.61 2.46 2.44 2.42 2.02 1.47 1.45 1.24 1.22 1.10 1.08 0.96 0.93 0.15 0.13
Dipp
N
N
H
B
4m
1.99 0.98 1.13 3.12 0.97 3.95 1.03 1.00 1.01 1.00 1.14 1.14 1.07 3.19 4.25 3.34 3.57 2.88
8.5 8.0 7.5 7.0 6.5 6.0 5.5 5.0 4.5 4.0 3.5 3.0 2.5 2.0 1.5 1.0 0.5
f1 (ppm)

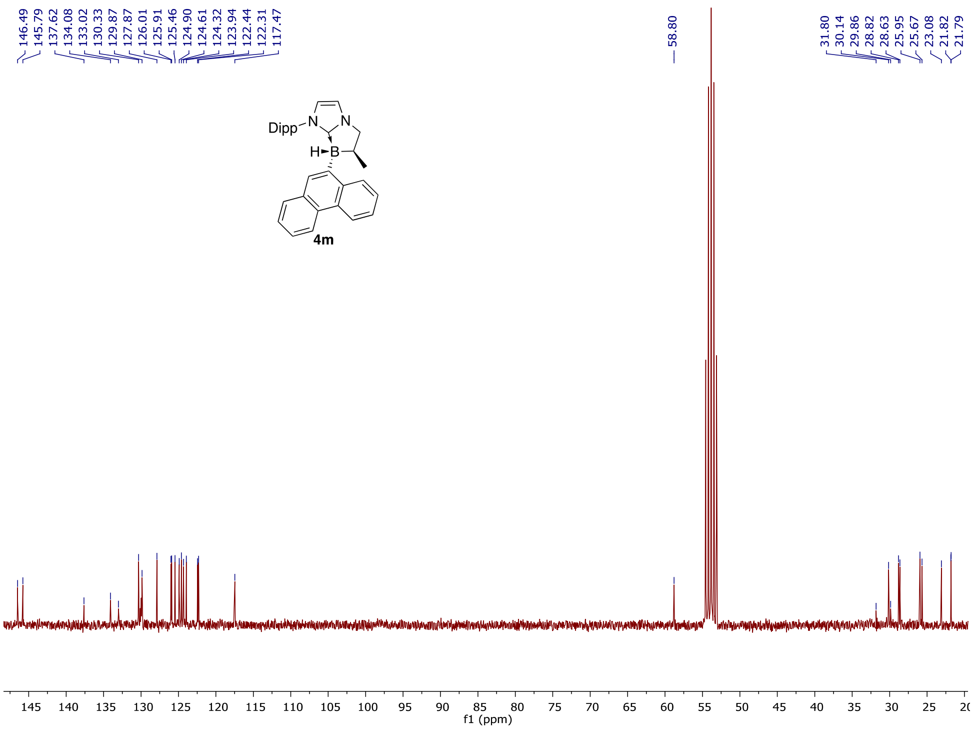

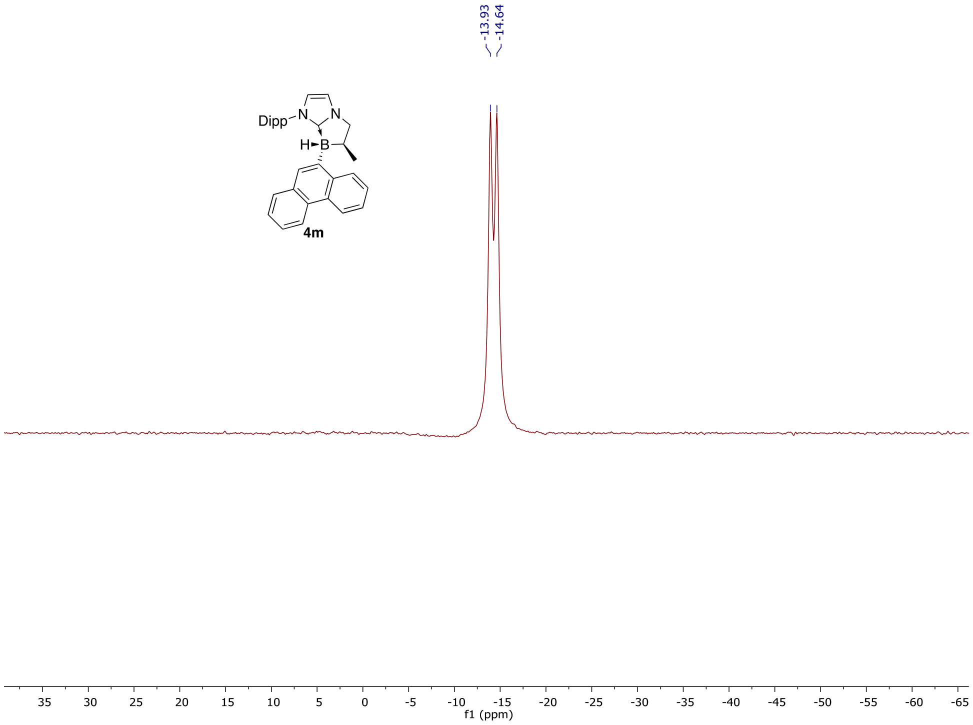

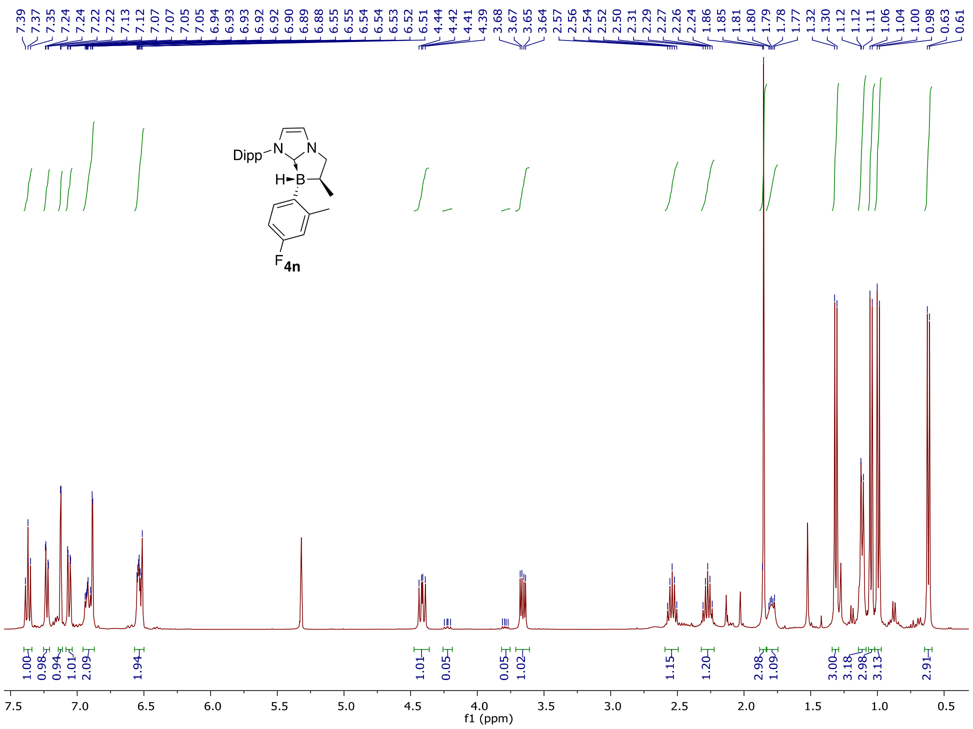

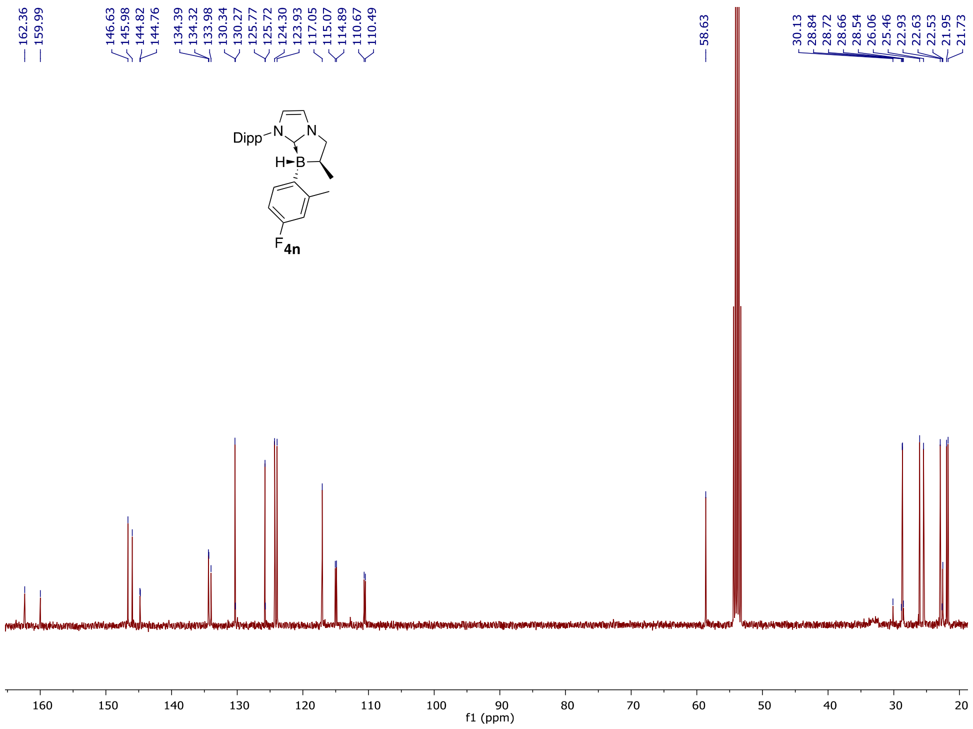

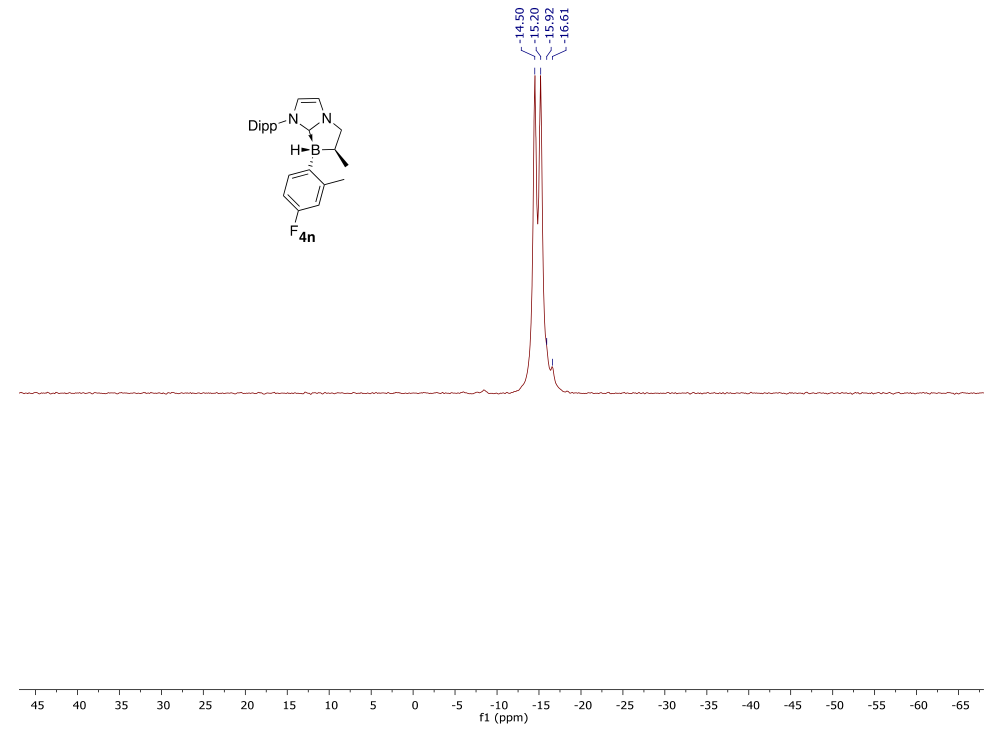

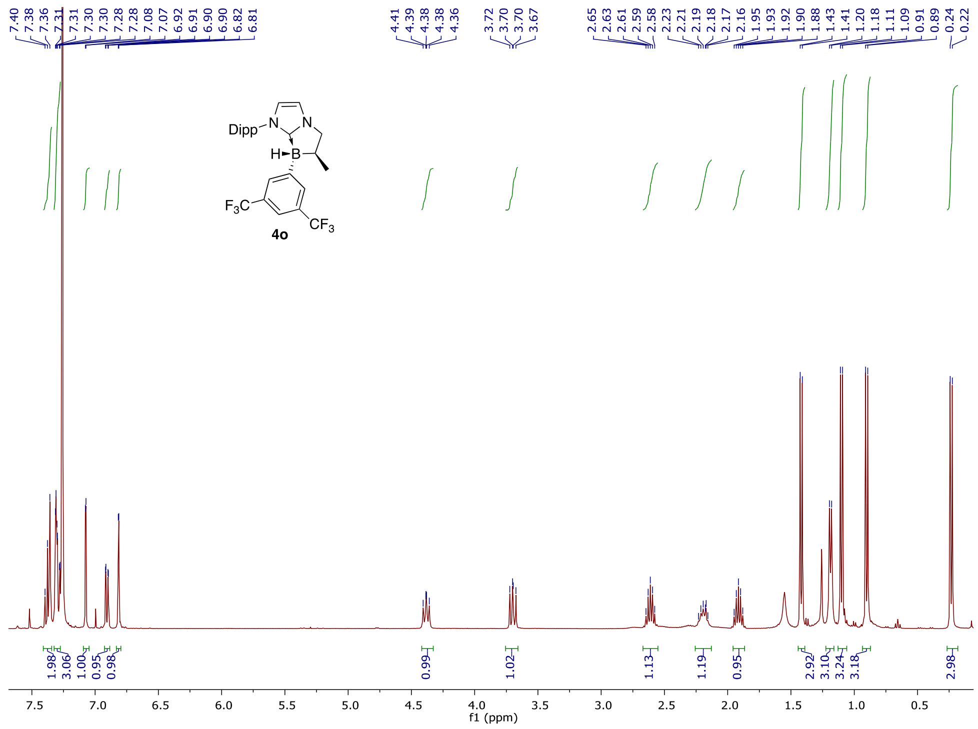

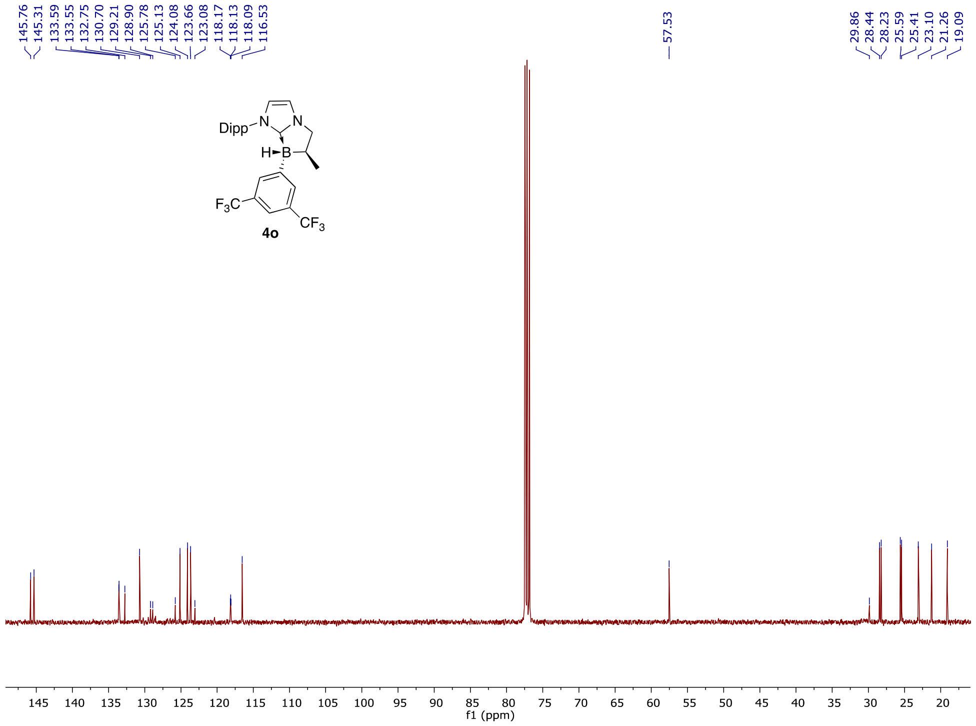
145.76
145.31
133.59
133.55
132.75
130.70
129.21
128.90
125.78
125.13
124.08
123.66
123.08
118.17
118.13
118.09
116.53
57.53
29.86
28.44
28.23
25.59
25.41
23.10
21.26
19.09
Dipp
N
N
H
B
F3C
CF3
4o
f1 (ppm)

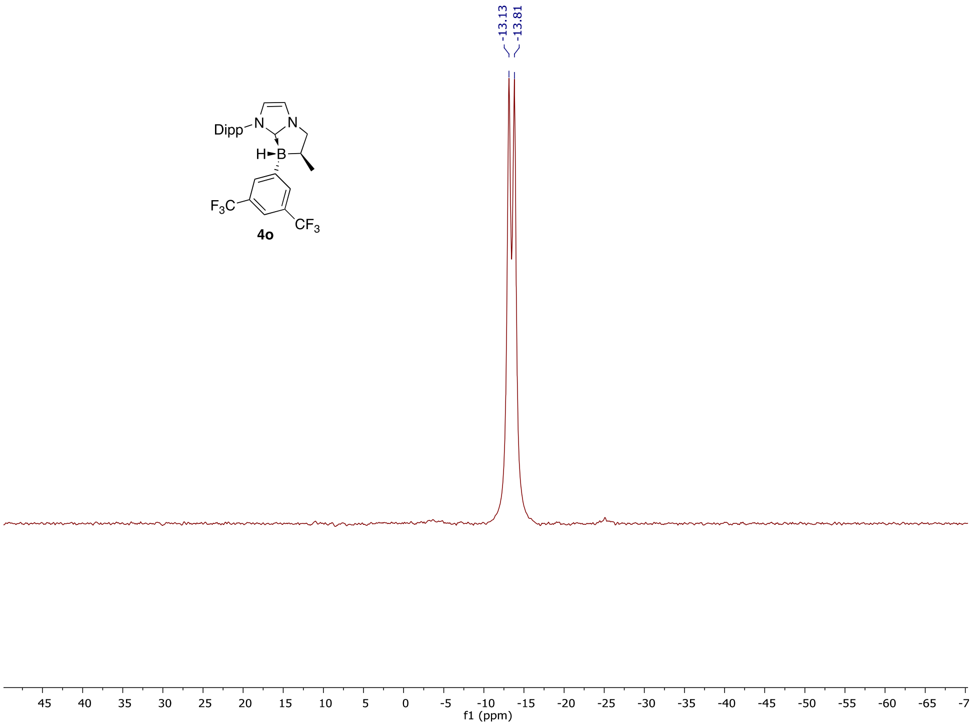
-13.13
-13.81
Dipp
N
N
H
B
F3C
CF3
4o
f1 (ppm)

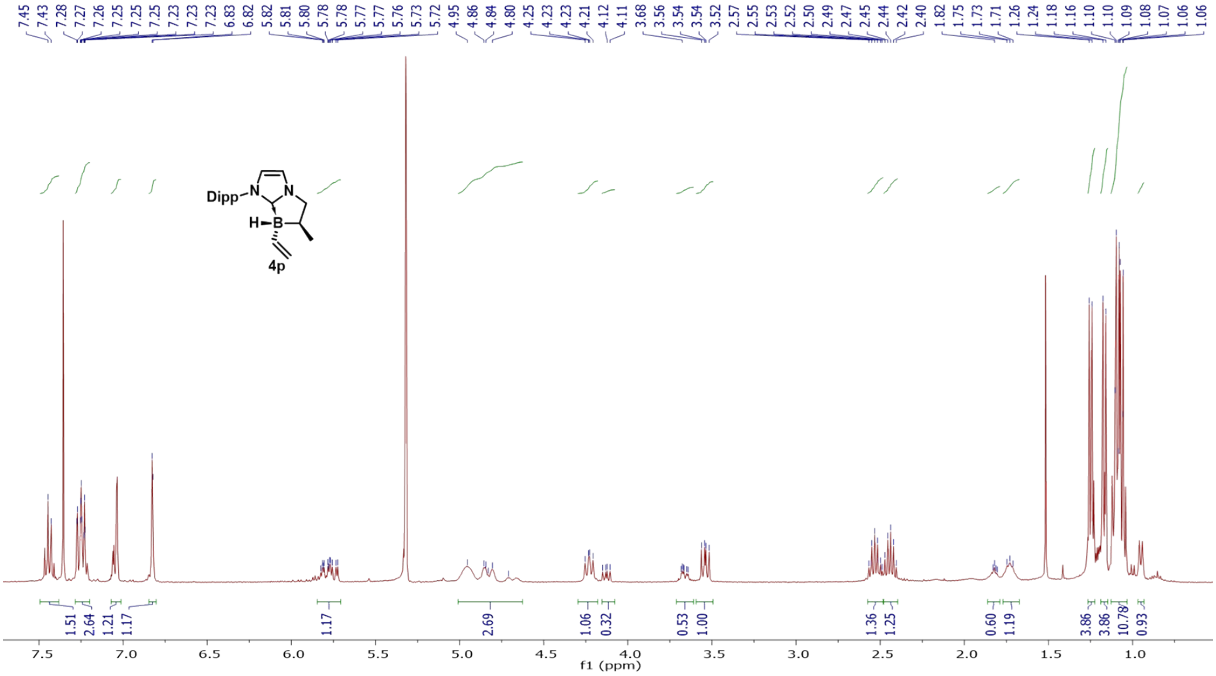
Dipp
N
N
H
B
4p
f1 (ppm)

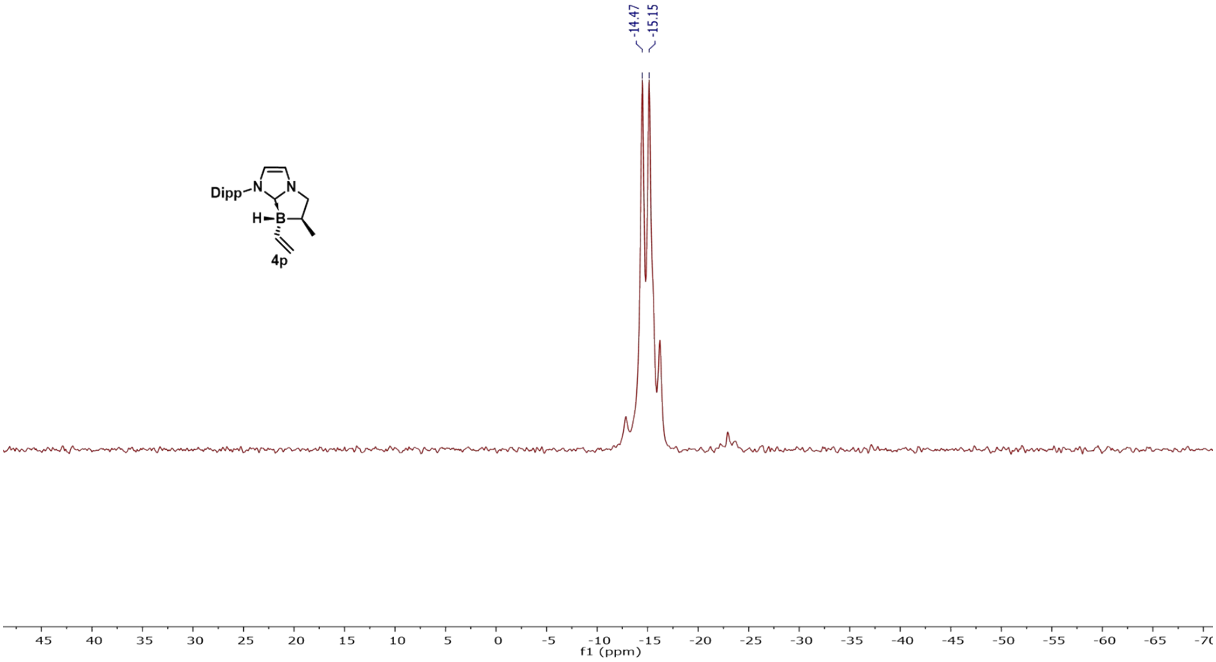
-14.47
-15.15
Dipp
N
N
H
B
4p
f1 (ppm)

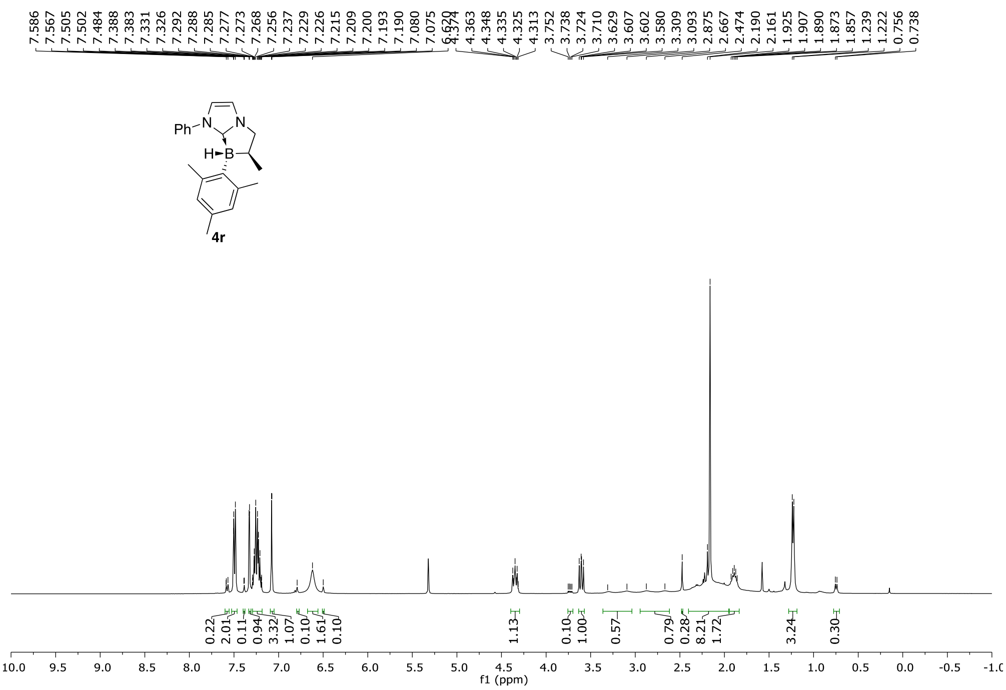
7.586 7.567 7.505 7.502 7.484 7.388 7.383 7.331 7.326 7.292 7.288 7.285 7.277 7.273 7.268 7.256 7.237 7.229 7.226 7.215 7.209 7.200 7.193 7.190 7.080 7.075 6.620 4.374 4.363 4.348 4.335 4.325 4.313 3.752 3.738 3.724 3.710 3.629 3.607 3.602 3.580 3.309 3.093 2.875 2.667 2.474 2.190 2.161 1.925 1.907 1.890 1.873 1.857 1.239 1.222 0.756 0.738
Ph
N
N
H
B
4r
0.22 2.01 0.11 0.94 3.32 1.07 0.10 1.61 0.10 1.13 0.10 1.00 0.57 0.79 0.28 8.21 1.72 3.24 0.30
f1 (ppm)

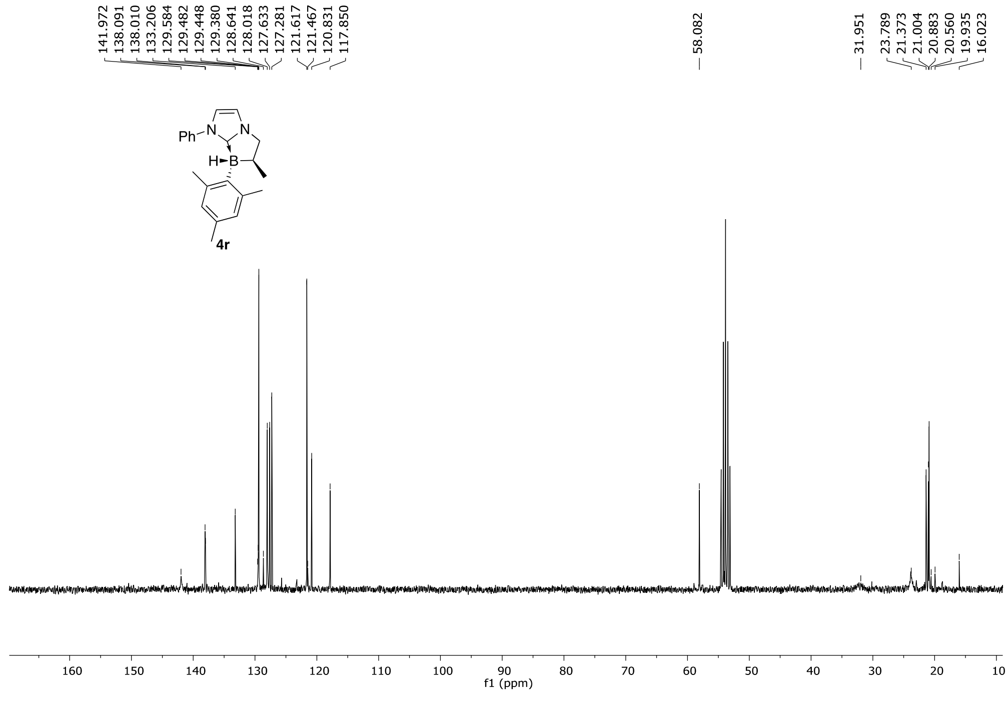
141.972 138.091 138.010 133.206 129.584 129.482 129.448 129.380 128.641 128.018 127.633 127.281 121.617 121.467 120.831 117.850
58.082
31.951 23.789 21.373 21.004 20.883 20.560 19.935 16.023
Ph
N
N
H
B
4r
f1 (ppm)

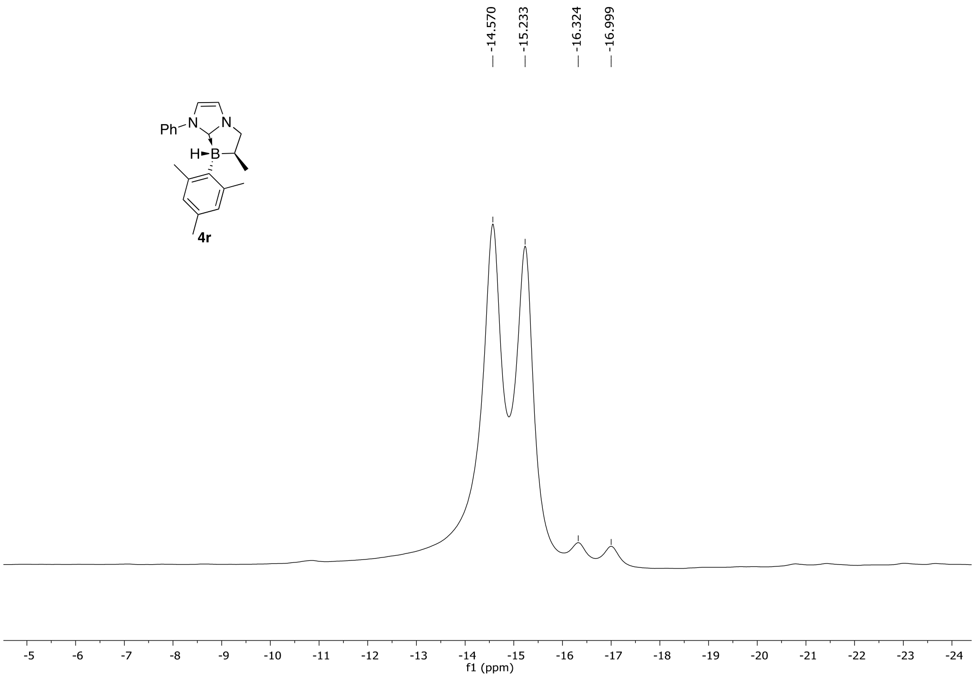

4r

-14.570
-15.233
-16.324
-16.999
-5 -6 -7 -8 -9 -10 -11 -12 -13 -14 -15 -16 -17 -18 -19 -20 -21 -22 -23 -24
f1 (ppm)

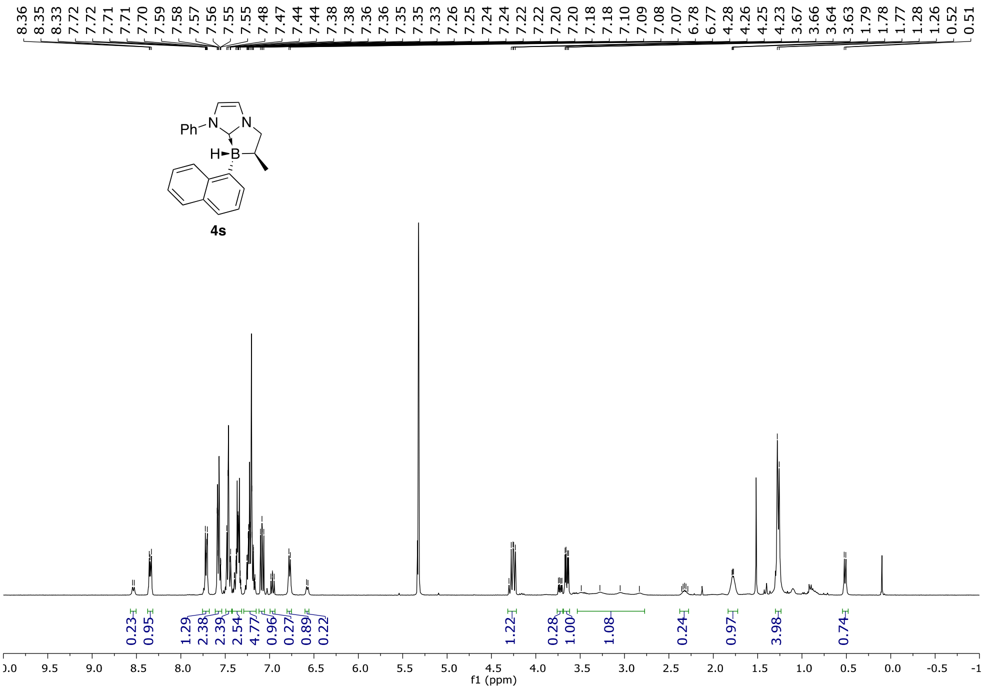

4s

8.36 8.35 8.33 7.72 7.72 7.71 7.71 7.70 7.59 7.58 7.57 7.56 7.55 7.55 7.48 7.47 7.44 7.44 7.38 7.38 7.36 7.36 7.35 7.35 7.33 7.26 7.25 7.24 7.24 7.22 7.22 7.20 7.20 7.18 7.10 7.09 7.08 7.07 6.78 6.77 4.28 4.26 4.25 4.23 3.67 3.66 3.64 3.63 1.79 1.78 1.77 1.28 1.26 0.52 0.51
0.23 0.95 1.29 2.38 2.39 2.54 4.77 0.96 0.27 0.89 0.22 1.22 0.28 1.00 1.08 0.24 0.97 3.98 0.74
9.5 9.0 8.5 8.0 7.5 7.0 6.5 6.0 5.5 5.0 4.5 4.0 3.5 3.0 2.5 2.0 1.5 1.0 0.5 0.0 -0.5
f1 (ppm)

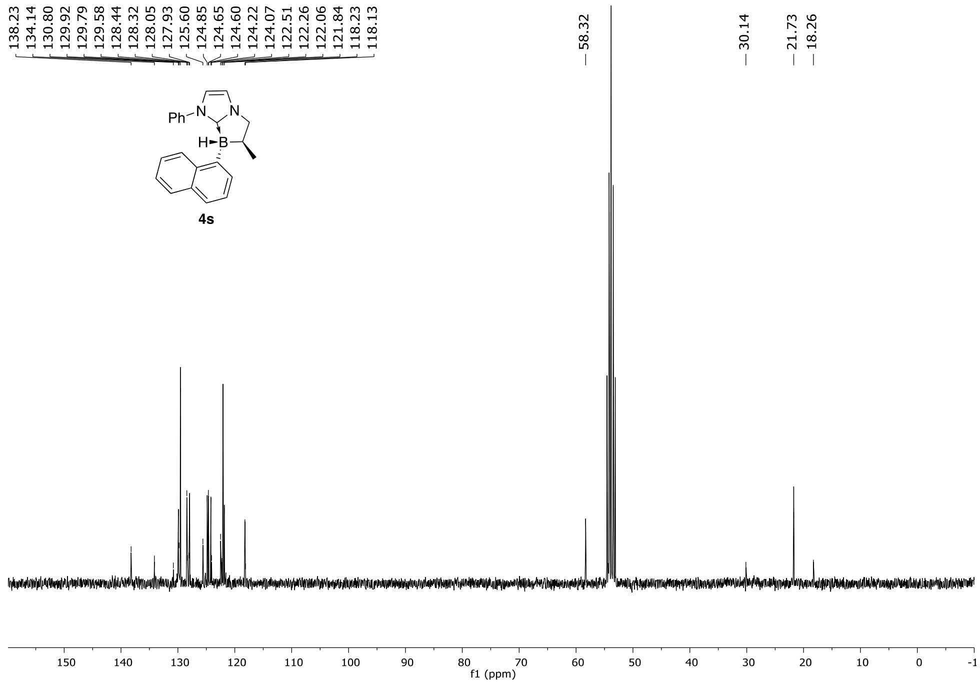
138.23
134.14
130.80
129.92
129.79
129.58
128.44
128.32
128.05
127.93
125.60
124.85
124.65
124.60
124.22
124.07
122.51
122.26
122.06
121.84
118.23
118.13
58.32
30.14
21.73
18.26
Ph
N
N
H
B
4s
150
140
130
120
110
100
90
80
70
60
50
40
30
20
10
0
-1
f1 (ppm)

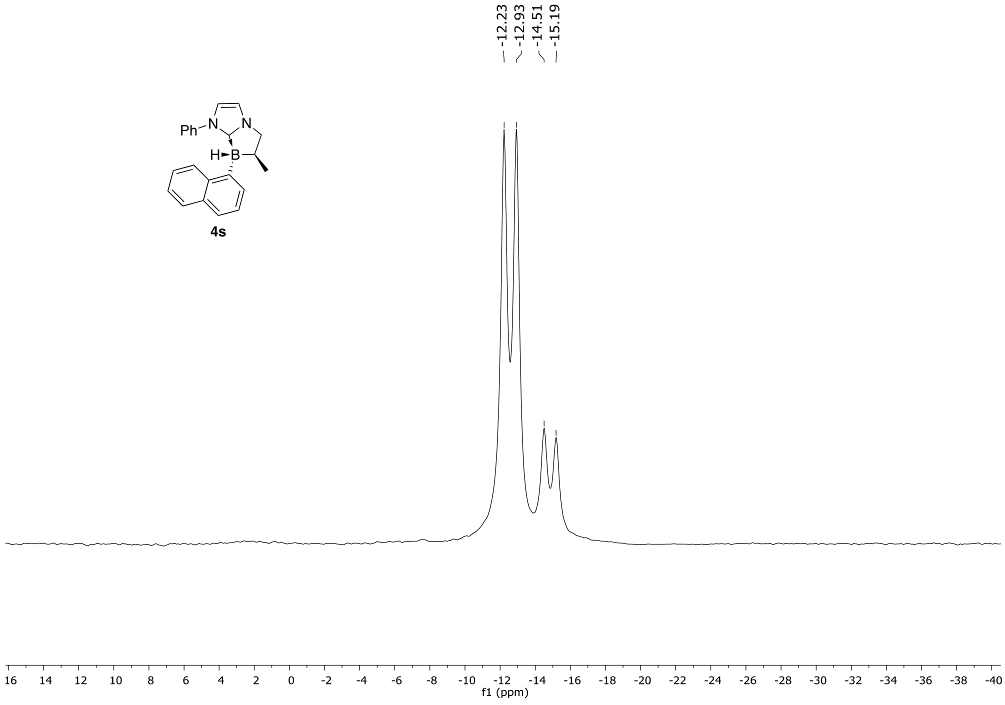
-12.23
-12.93
-14.51
-15.19
Ph
N
N
H
B
4s
16
14
12
10
8
6
4
2
0
-2
-4
-6
-8
-10
-12
-14
-16
-18
-20
-22
-24
-26
-28
-30
-32
-34
-36
-38
-40
f1 (ppm)

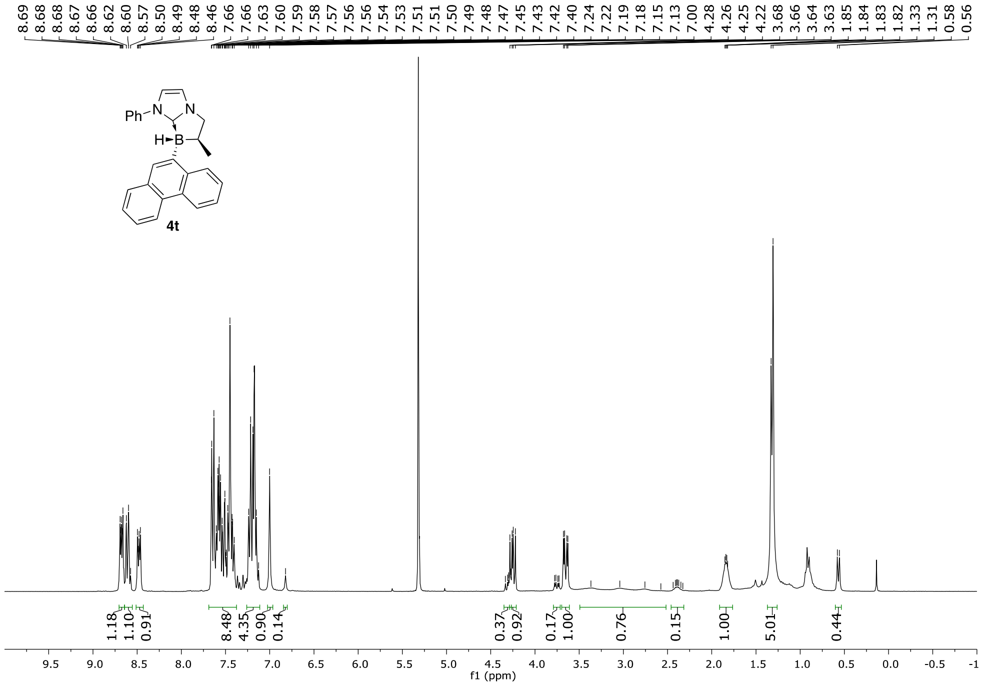
8.69 8.68 8.67 8.66 8.62 8.60 8.57 8.50 8.49 8.48 8.46 7.66 7.66 7.63 7.60 7.59 7.58 7.57 7.56 7.56 7.54 7.53 7.51 7.51 7.50 7.49 7.48 7.47 7.45 7.43 7.42 7.40 7.24 7.22 7.19 7.18 7.15 7.13 7.00 4.28 4.26 4.25 4.22 3.68 3.66 3.64 3.63 1.85 1.84 1.83 1.82 1.33 1.31 0.58 0.56
Ph
N
N
H
B
4t
1.18 1.10 0.91 8.48 4.35 0.90 0.14 0.37 0.92 0.17 1.00 0.76 0.15 1.00 5.01 0.44
f1 (ppm)

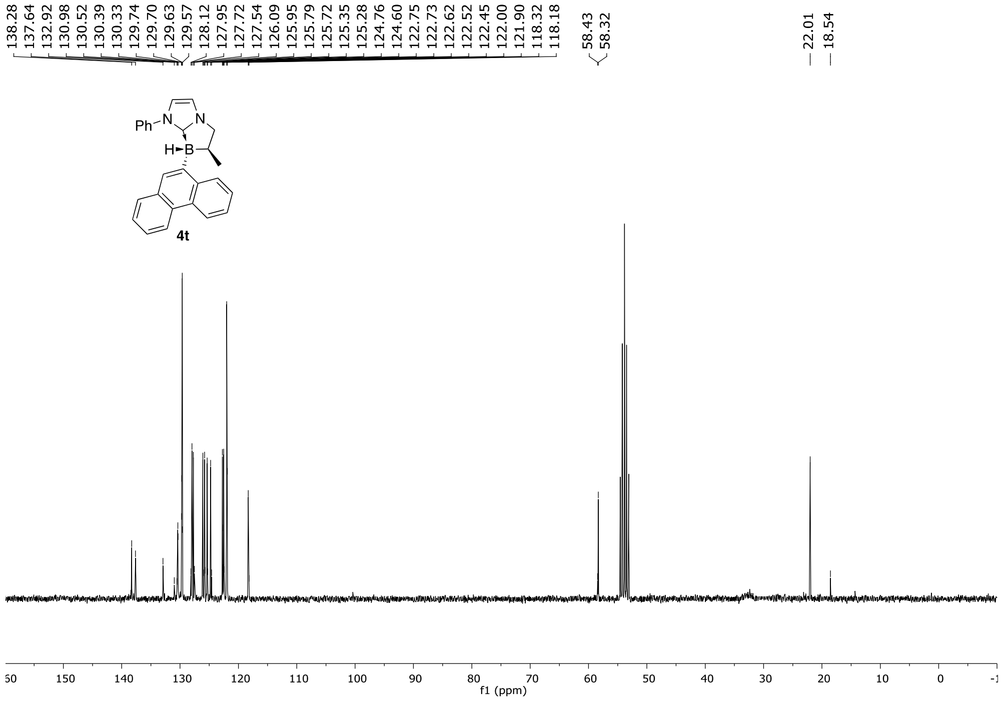
138.28 137.64 132.92 130.98 130.52 130.39 130.33 129.74 129.70 129.63 129.57 128.12 127.95 127.72 127.54 126.09 125.95 125.79 125.72 125.35 125.28 124.76 124.60 122.75 122.73 122.62 122.52 122.45 122.00 121.90 118.32 118.18
58.43 58.32
22.01
18.54
Ph
N
N
H
B
4t
f1 (ppm)

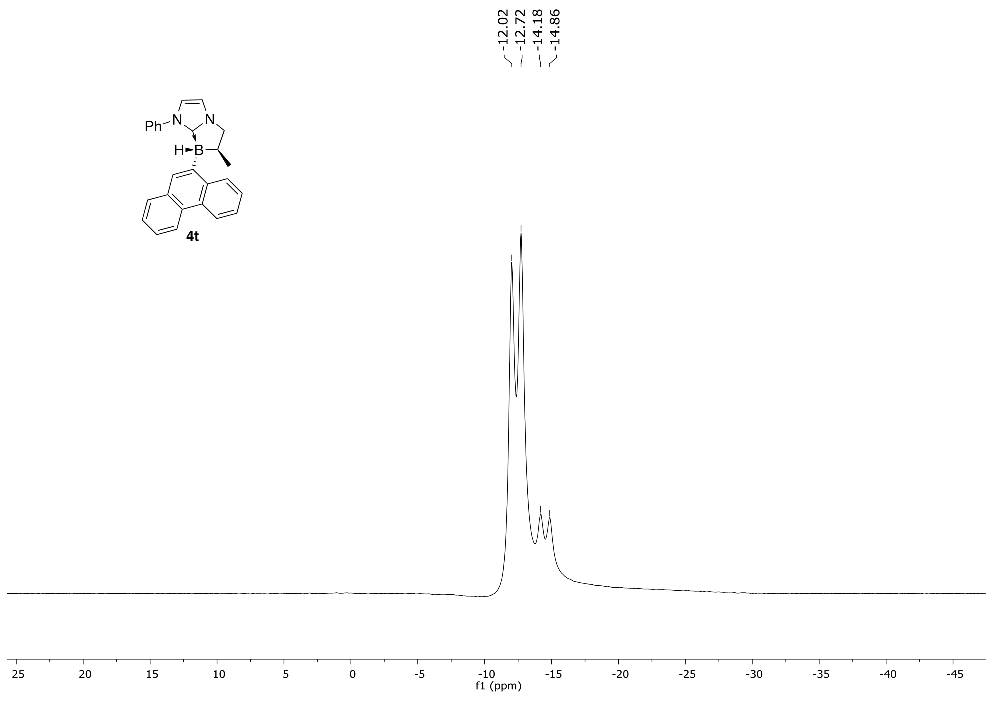

-12.02
-12.72
-14.18
-14.86
Ph
N
N
H
B
4t
25
20
15
10
5
0
-5
-10
-15
-20
-25
-30
-35
-40
-45
f1 (ppm)

5. Modeling approach for *trans*-5a, *cis*- & *trans*-5a•-, IIa and IIIa

Geometry optimizations and frequency calculations for reactants, intermediates and products were performed at the DFT level of theory by using the M06-2X functional[1] as implemented in the Gaussian 09 or 16.[2] For all the atoms, all-electron standard 6-311g(d,p) basis set was, employed. For each optimized stationary point vibrational analysis was performed to establish its nature as a minimum or saddle point, and zero-point vibrational energy (ZPE) corrections were included in all relative energies (ΔE). NBO 6 program,[3] which is included in Gaussian 09 suite of programs, was used to obtain natural bond orbitals (NBOs), atomic net charges, and densities of spin at the M06-2X/6.311g(d,p) optimized geometries.

a. *trans*-NHC-borane **5a**

(M06-2x/6-311g(d,p))

C	-2.89433388	0.49770290	0.00000000	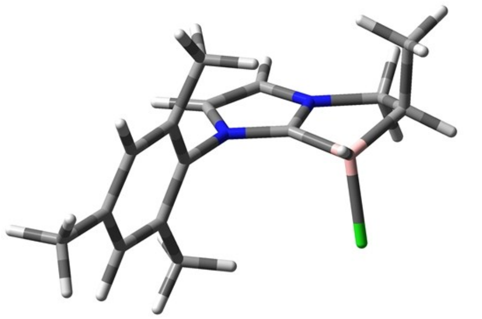
C	-1.53572188	0.52503490	0.03805400	
C	-2.16468088	0.62678690	-2.10229800	
N	-3.26368088	0.56418590	-1.33550500	
H	-3.62825288	0.42275790	0.78427000	
H	-0.85569388	0.49152490	0.87128000	
N	-1.11937588	0.59468790	-1.27089300	
C	0.18613012	0.74080990	-1.94835900	
H	0.91336412	0.08030590	-1.46948300	
H	0.50523812	1.77946090	-1.83963300	
C	-0.13136988	0.38132890	-3.41350500	
C	0.03365612	-1.12539510	-3.63510700	
H	-0.64039388	-1.69212310	-2.98155400	
H	-0.21707988	-1.39030610	-4.66372500	
B	-1.69422588	0.83390890	-3.63278900	
H	1.05425812	-1.46656810	-3.43341500	
H	0.54187212	0.93306090	-4.07241700	
C	-4.61069288	0.54583390	-1.83807500	

---

[1] Zhao, Y.; Truhlar, D. G. *Theor. Chem. Acc.*, **2008**, *120*, 215-241.

[2] Gaussian 09, Revision A.1, M. J. Frisch, G. W. Trucks, H. B. Schlegel, G. E. Scuseria, M. A. Robb, J. R. Cheeseman, G. Scalmani, V. Barone, B. Mennucci, G. A. Petersson, H. Nakatsuji, M. Caricato, X. Li, H. P. Hratchian, A. F. Izmaylov, J. Bloino, G. Zheng, J. L. Sonnenberg, M. Hada, M. Ehara, K. Toyota, R. Fukuda, J. Hasegawa, M. Ishida, T. Nakajima, Y. Honda, O. Kitao, H. Nakai, T. Vreven, J. A. Montgomery, Jr., J. E. Peralta, F. Ogliaro, M. Bearpark, J. J. Heyd, E. Brothers, K. N. Kudin, V. N. Staroverov, R. Kobayashi, J. Normand, K. Raghavachari, A. Rendell, J. C. Burant, S. S. Iyengar, J. Tomasi, M. Cossi, N. Rega, J. M. Millam, M. Klene, J. E. Knox, J. B. Cross, V. Bakken, C. Adamo, J. Jaramillo, R. Gomperts, R. E. Stratmann, O. Yazyev, A. J. Austin, R. Cammi, C. Pomelli, J. W. Ochterski, R. L. Martin, K. Morokuma, V. G. Zakrzewski, G. A. Voth, P. Salvador, J. J. Dannenberg, S. Dapprich, A. D. Daniels, Ö. Farkas, J. B. Foresman, J. V. Ortiz, J. Cioslowski, and D. J. Fox, Gaussian, Inc., Wallingford CT, **2009**.

[3] NBO 6.0. Glendening, E. D.; Badenhoop, J. K.; Reed, A. E.; Carpenter, J. E.; Bohmann, J. A.; Morales, C. M.; Landis, C. R.; Weinhold, F. (Theoretical Chemistry Institute, University of Wisconsin, Madison, WI, 2013); http://nbo6.chem.wisc.edu/

C	-5.35448888	1.72569690	-1.80162200	
C	-5.10514888	-0.64751610	-2.36331200	
C	-6.65491588	1.67737990	-2.29479700	
C	-6.41061288	-0.64430210	-2.84642900	
C	-7.19873288	0.50406190	-2.81449800	
H	-7.25186388	2.58389690	-2.28742500	
H	-6.81742688	-1.55964010	-3.26450700	
C	-4.23994288	-1.87718610	-2.43761000	
H	-3.45308988	-1.74165810	-3.18547300	
H	-3.75303088	-2.08135110	-1.48077500	
H	-4.83359888	-2.74738110	-2.71632700	
C	-4.74734488	3.00373590	-1.28889500	
H	-5.45494988	3.82646790	-1.38482700	
H	-4.46159288	2.91904690	-0.23725500	
H	-3.84758688	3.24987590	-1.85952900	
C	-8.61795488	0.47742690	-3.31886800	
H	-8.75529688	-0.30312710	-4.06810200	
H	-9.31345488	0.28027390	-2.49839300	
H	-8.89359488	1.43408690	-3.76495000	
Cl	-1.83277388	2.72199290	-3.91553200	
H	-2.31914288	0.24460990	-4.46731200	

b. NHC-borane radical anion **5a•-**

- *trans*-NHC-boron radical anion 5a•- (uM06-2x/6-311g(d,p))

Sum of electronic = -1178.1099 (Hartree/Particule)

C	-3.87957335	2.97256093	0.00000000	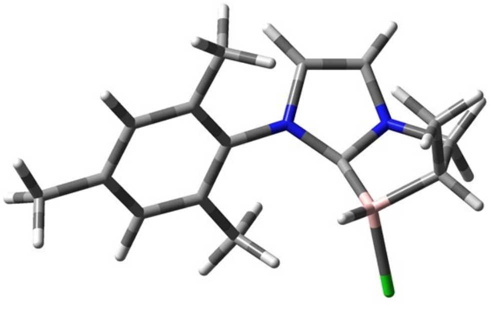
C	-2.54486335	2.98949493	0.20847500	
C	-3.05449135	4.87806793	-0.96114900	
N	-4.22846135	4.14854193	-0.69217600	
H	-4.63282335	2.29763993	0.36762200	
H	-1.93179235	2.27308093	0.73235300	
N	-2.03607135	4.16693193	-0.29172500	
C	-0.72914335	4.42281893	-0.90926700	
H	-0.09469235	3.53588493	-0.79331500	
H	-0.25701735	5.26901693	-0.40055300	
C	-1.05108435	4.78426193	-2.37120700	
C	-1.27256135	3.50001393	-3.17659300	
H	-2.09078535	2.91627493	-2.73647100	
H	-1.55604535	3.73018393	-4.20687800	
B	-2.46927235	5.61037493	-2.24213100	
H	-0.38070335	2.85923793	-3.19898900	

H	-0.23608335	5.37092293	-2.80516300	
C	-5.52891035	4.51270493	-1.04275900	
C	-6.02011935	5.80554593	-0.73267600	
C	-6.36833635	3.58014293	-1.69588000	
C	-7.33228135	6.11108193	-1.05471800	
C	-7.68652535	3.93705193	-1.97375800	
C	-8.19518135	5.19278693	-1.66757500	
H	-7.70811135	7.09897193	-0.79855900	
H	-8.32162335	3.21730493	-2.48562900	
C	-5.82906235	2.26160093	-2.19086900	
H	-4.81775835	2.39244093	-2.58386300	
H	-5.76754035	1.49499993	-1.41094300	
H	-6.46774035	1.87385593	-2.98696700	
C	-5.16550435	6.80777993	-0.00747300	
H	-5.78568735	7.63034493	0.35608700	
H	-4.66153535	6.33861893	0.84139700	
H	-4.37446135	7.20849693	-0.64671400	
C	-9.61646335	5.57437993	-1.99221300	
H	-10.13756435	4.75445493	-2.49153100	
H	-10.18113735	5.82826493	-1.08878700	
H	-9.65437935	6.44647893	-2.65270000	
Cl	-1.98897135	7.52514993	-1.85127600	
H	-3.17803135	5.64696293	-3.21441000	

Contour plots:

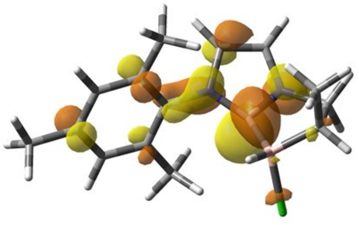

Somo (isoval 0.05)

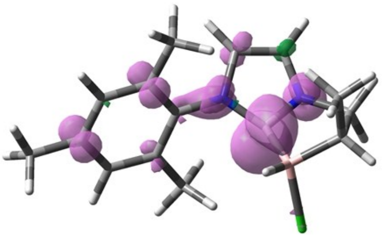

Spin density (0.004)

- *cis*-NHC-boron radical anion 5a•- (uM06-2x/6-311g(d,p))

Sum of electronic = -1178.1046082 (Hartree/Particule)

C	-3.08689038	2.42378045	0.00000000
C	-4.40047038	2.46290745	0.30702200
C	-3.64989438	0.37736745	0.86283000
N	-2.58914838	1.15511645	0.35516100
H	-2.43005038	3.20483445	-0.34104000
H	-5.09750138	3.28014645	0.20607200
N	-4.74768838	1.26741945	0.90181600
C	-6.01145138	0.53419645	0.82346800
H	-6.77848738	1.17870145	0.37804000
H	-6.32676738	0.26949045	1.83962000
C	-5.70054938	-0.72956155	-0.00220400
C	-5.75637038	-0.37204455	-1.48970300
H	-5.01201838	0.39976945	-1.71984300
H	-5.51656438	-1.23556555	-2.11216700
B	-4.16725638	-1.11127455	0.51568800
H	-6.74428238	0.00971645	-1.78151200
H	-6.44121738	-1.50654255	0.21875200
C	-1.25870238	0.75891945	0.21994600
C	-0.58094738	0.12263845	1.28830600
C	-0.57729538	1.00022145	-0.99522900
C	0.76174862	-0.18333255	1.13667400
C	0.77804462	0.69379145	-1.08096300
C	1.47673762	0.11065645	-0.03021300
H	1.27823862	-0.65986355	1.96717700
H	1.29018362	0.87201345	-2.02395500
C	-1.33194938	1.42631145	-2.22888700
H	-2.28275938	0.88714245	-2.27272900
H	-1.56107638	2.49633145	-2.26249800
H	-0.75085538	1.18112445	-3.12010900
C	-1.28123738	-0.18483355	2.58442500
H	-0.55415838	-0.51084555	3.33230900
H	-1.80851938	0.69597945	2.95880900
H	-2.03870338	-0.96109655	2.44811700
C	2.94325462	-0.21904055	-0.13897600
H	3.30505862	-0.05824055	-1.15710600
H	3.54873562	0.40269745	0.53009200
H	3.13790662	-1.26313655	0.12433100
Cl	-3.06378938	-1.89572555	-0.88703600
H	-4.14043338	-1.91285255	1.42467600

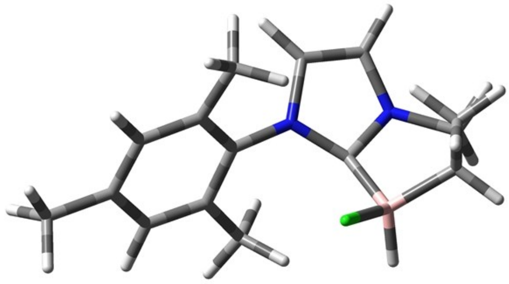

Contour plots:

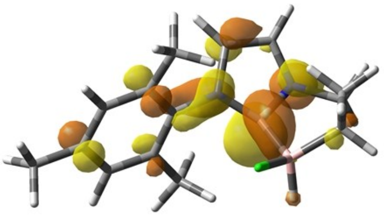

Somo (isoval 0.05)

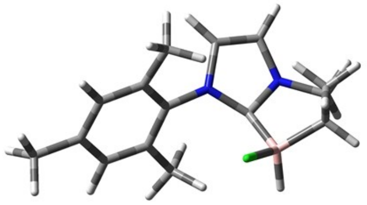

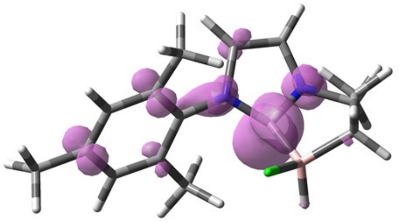

Spin density (0.004)

c. NHC-boryl radical **IIa**

(uM06-2x/6-311g(d,p))

Sum of electronic and zero-point Energies= -717.811978 (Hartree/Particle)

Sum of electronic and thermal Energies= -717.793643 (Hartree/Particle)

Sum of electronic and thermal Enthalpies= -717.792698 (Hartree/Particle)

Sum of electronic and thermal Free Energies= -717.860882 (Hartree/Particle)

C	0.29687499	1.01562498	0.00000000
C	-1.06418801	0.95430798	0.02849300
C	-0.40038001	0.51938898	-2.10516500
N	0.71462999	0.74637098	-1.30828300
H	1.00765999	1.21755698	0.78631400
H	-1.74715201	1.09796398	0.85228600
N	-1.48170701	0.66101398	-1.24833200
C	-2.77514401	0.38886098	-1.88320000
H	-3.46208501	1.22288798	-1.69158900
H	-3.21233101	-0.51311002	-1.43568100
C	-2.45548601	0.20242998	-3.40348300
C	-3.10181101	1.30764598	-4.26367900
H	-2.73135201	2.29979198	-3.97525700
H	-2.85728801	1.16508798	-5.32225100
B	-0.83555201	0.18728198	-3.49668500
H	-4.19787501	1.32086798	-4.17254800
H	-2.88237601	-0.75712302	-3.72936800
C	2.07453599	0.72150198	-1.75684300
C	2.71896199	-0.51531102	-1.92619200
C	2.72810499	1.93654498	-2.02670100
C	4.04753799	-0.51048802	-2.36269800
C	4.05773299	1.88890998	-2.45393800
C	4.73610799	0.67746298	-2.62468300
H	4.55588899	-1.46248702	-2.50073400

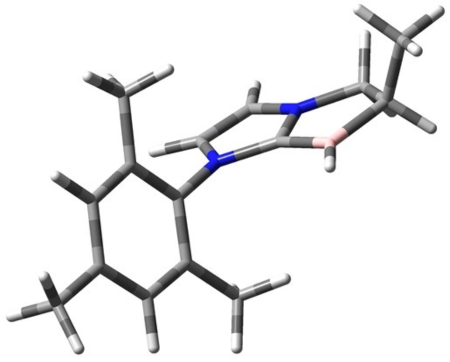

H	4.57208299	2.82298298	-2.67125300	
C	2.00709799	3.25632098	-1.88654200	
H	1.07764199	3.26034798	-2.46694500	
H	1.73181499	3.46593498	-0.84566300	
H	2.63548399	4.07956398	-2.23901000	
C	1.99737199	-1.81294502	-1.65436200	
H	2.66486999	-2.66694602	-1.80266300	
H	1.61562399	-1.85295102	-0.62717800	
H	1.13303099	-1.92958402	-2.31884700	
C	6.18174599	0.65746098	-3.06433000	
H	6.39343199	1.45798698	-3.78161800	
H	6.85889999	0.80012498	-2.21142800	
H	6.44492499	-0.29604302	-3.53359700	
H	-0.17273901	-0.00001102	-4.47126900	

Contour plots:

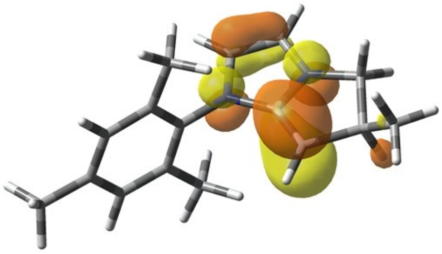

Somo (isoval 0.05)

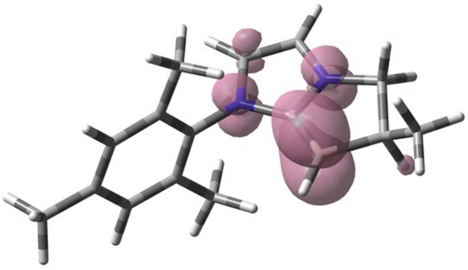

Spin density (0.004)

NBO charges: B (0.378); C_{NHC} (0.118)

d. NHC-boryl radical **IIIa**

(uM06-2x/6-311g(d,p))

Sum of electronic and zero-point Energies= -1066.295511 (Hartree/Particle)

Sum of electronic and thermal Energies= -1066.268229 (Hartree/Particle)

Sum of electronic and thermal Enthalpies= -1066.267284 (Hartree/Particle)

Sum of electronic and thermal Free Energies= -1066.353356 (Hartree/Particle)

C	-2.14176839	1.37195120	0.00000000	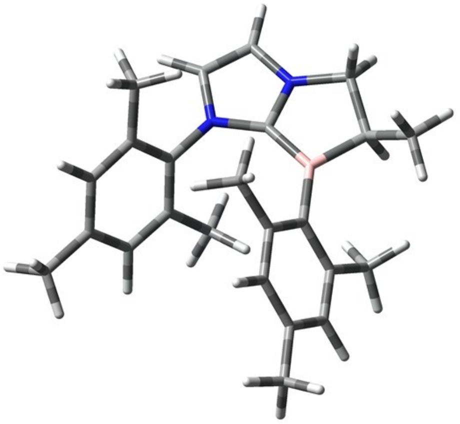
C	-0.83641239	1.02226220	-0.10488600	
C	-0.96277139	3.27219420	-0.33232300	
N	-2.22610039	2.75745220	-0.13480700	
H	-3.01555539	0.77600020	0.20009600	
H	-0.37227339	0.05300820	-0.03955200	
N	-0.12371039	2.18283720	-0.27842500	
C	1.24249961	2.50209920	-0.68983400	
H	1.36531561	2.22144620	-1.74383000	
H	1.95998361	1.93841620	-0.09027500	
C	1.34312761	4.03821820	-0.52982200	
C	2.25191261	4.66953020	-1.58468100	
H	1.85144461	4.49125120	-2.58756700	
H	2.30168261	5.75259220	-1.44731200	
B	-0.19734239	4.55410920	-0.55314500	
H	3.27327161	4.27608020	-1.54999800	
H	1.77478961	4.23415820	0.46067500	
C	-3.43593839	3.51258720	-0.11384300	
C	-3.63654839	4.47084220	0.88464400	
C	-4.38945539	3.28308020	-1.11538900	
C	-4.81360639	5.21679620	0.84552500	
C	-5.55729339	4.03765120	-1.09756000	
C	-5.78393939	5.01535620	-0.12964700	
H	-4.97105139	5.97580520	1.60585000	
H	-6.29688539	3.87825920	-1.87678100	
C	-4.13501839	2.29474120	-2.22414500	
H	-3.10539839	2.36621320	-2.58162100	
H	-4.29288039	1.26385420	-1.89630800	
H	-4.80807239	2.48610020	-3.06014400	
C	-2.62722639	4.71874620	1.97288600	
H	-3.13061139	5.06135020	2.87796100	
H	-2.05767839	3.81815620	2.20582700	
H	-1.91958639	5.49186420	1.66011000	
C	-7.06049139	5.81541020	-0.12940700	
H	-7.42439139	5.97467820	-1.14576900	
H	-7.84442039	5.29182620	0.42500700	
H	-6.91345239	6.78953320	0.33875200	
C	-0.71213039	6.03310220	-0.69007200	
C	-0.21656739	7.03966120	0.17680600	
C	-1.67605739	6.41573020	-1.64255900	
C	-0.68607439	8.34474520	0.09171900	
C	-2.12617939	7.73736620	-1.70413200	
C	-1.64691739	8.71756020	-0.84835300	
H	-0.29933639	9.09306420	0.77894900	
H	-2.87275639	8.00153820	-2.44948100	

C	0.80991961	6.71223420	1.23607800	
H	1.78843961	6.50787320	0.79168600	
H	0.92764961	7.54352620	1.93270200	
H	0.52448861	5.82339320	1.80664600	
C	-2.12036639	10.14504320	-0.94373400	
H	-1.39024139	10.76202820	-1.47545900	
H	-3.06711939	10.21094520	-1.48220300	
H	-2.25892739	10.58182620	0.04765900	
C	-2.25339239	5.45004120	-2.65253000	
H	-3.32575839	5.31517720	-2.48580500	
H	-2.12239839	5.84175020	-3.66514500	
H	-1.77292639	4.47247820	-2.60324000	

Contour plots:

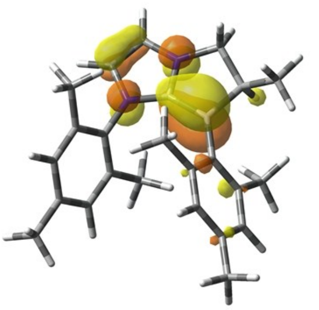

Somo (isoval 0.05)

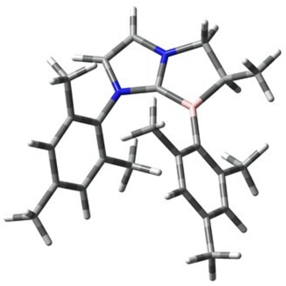

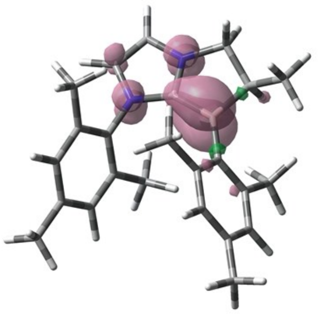

Spin density (0.004)

NBO charges: B (0.521); C_{NHC} (0.093)

6. Origin of the diastereoselectivity in the formation of NHC-boranes 4a and 4d

a. DFT calculations

To determine the origin of the diastereoselectivity in the formation of the boron stereogenic NHC-boranes **4**, we hypothesized that (i) the boron stereocenter could isomerize through a thermal equilibration *via* the intermediate radical and (ii) a preferential addition *anti* to the methyl substituent could occur. To acquire more insights, DFT calculations (M06-2X/6-311G(d,p), continuum solvent model PCM for diethylether) on *trans* and *cis*-**4a/d** were performed and clearly indicated a large preference for the *trans*-diastereomer in agreement with the experimental diastereomeric ratio.

- NHC-borane *trans*-**4a** (M062X/6-311g(d,p)) //pcm (ether)
Sum of electronic and zero-point Energies= -1066.911673 (Hartree/Particle)
Sum of electronic and thermal Energies= -1066.884639 (Hartree/Particle)
Sum of electronic and thermal Enthalpies= -1066.883695 (Hartree/Particle)
Sum of electronic and thermal Free Energies= -1066.967892 (Hartree/Particle)

C	0.14062499	2.64062496	0.00000000
C	-1.19068001	2.86303296	0.16046400
C	-0.10862501	4.04233796	1.72125200
N	0.79220399	3.38166396	0.97549600
H	0.68139199	2.03307196	-0.70535100
H	-2.03616901	2.48625896	-0.38839200
N	-1.30993801	3.72529996	1.23072200
C	-2.43905501	4.40038696	1.89913900
H	-3.18562001	3.64713596	2.16560900
H	-2.88750701	5.09754196	1.18835100
C	-1.81024101	5.09133496	3.13833800
C	-2.20378401	4.31759696	4.40165200
H	-1.82051001	3.29218196	4.35349700
H	-1.76277101	4.78150496	5.28570700
B	-0.13820701	5.07787496	2.94963600
H	-3.28942501	4.26771296	4.54512400
H	-2.21435101	6.10308196	3.21707500
C	2.20002099	3.40210996	1.26619300
C	3.00093099	4.41266296	0.74449400
C	2.68667899	2.43387396	2.15524400
C	4.34465399	4.42812996	1.13003000

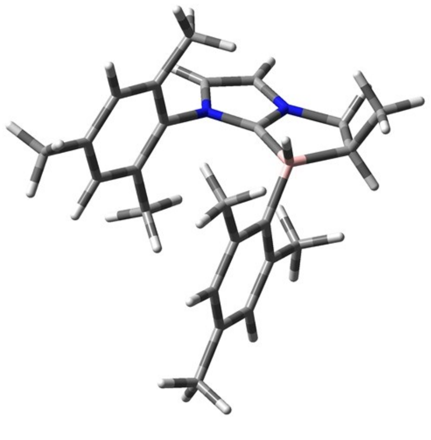

C	4.03136699	2.48106196	2.49233700	
C	4.87489499	3.47587896	1.98944100	
H	4.98164199	5.22111196	0.75031500	
H	4.42859999	1.74373196	3.18342400	
C	0.69567099	6.42954496	2.56314300	
C	1.95601299	6.68099296	3.15250100	
C	0.25908299	7.35345196	1.58830600	
C	2.69740399	7.81280496	2.80345600	
C	1.01190599	8.48465396	1.27025600	
C	2.23726399	8.73993796	1.87493600	
H	3.66362499	7.97479496	3.27474100	
H	0.63820799	9.17594596	0.51891100	
C	1.75399299	1.42254896	2.76624200	
H	1.00192799	1.92942496	3.37881400	
H	1.22283999	0.84947796	2.00232200	
H	2.30422899	0.72718896	3.39926500	
C	2.47991799	5.48101096	-0.17759000	
H	3.01681099	5.44941396	-1.12909100	
H	1.41388199	5.37389096	-0.37580900	
H	2.63624999	6.46404896	0.27378000	
C	6.32868099	3.50323696	2.38265200	
H	6.44090599	3.40474796	3.46436500	
H	6.86894099	2.67456696	1.91723000	
H	6.80421699	4.43361496	2.07166000	
C	2.57315899	5.73819496	4.16118300	
H	2.69878699	4.73675896	3.74274900	
H	3.55428599	6.10262496	4.47188800	
H	1.94416799	5.63821396	5.04787200	
C	-1.01354101	7.13620296	0.80159300	
H	-1.00521401	6.16477496	0.29637100	
H	-1.89872701	7.17024396	1.43867200	
H	-1.12734701	7.90257296	0.03324500	
C	3.02664599	9.98199296	1.54922200	
H	2.87271599	10.28815896	0.51256400	
H	2.72055599	10.81556296	2.18802200	
H	4.09529399	9.82259496	1.70509400	
H	0.34868899	4.52571996	3.90987600	

- NHC-borane *cis*-**4a** (M062X/6-311g(d,p))//pcm (ether)

Sum of electronic and zero-point Energies= -1066.906909 (Hartree/Particle)

Sum of electronic and thermal Energies= -1066.880374 (Hartree/Particle)

Sum of electronic and thermal Enthalpies= -1066.879430 (Hartree/Particle)

Sum of electronic and thermal Free Energies= -1066.961136 (Hartree/Particle)

C	-0.93749997	0.98437499	0.00000000	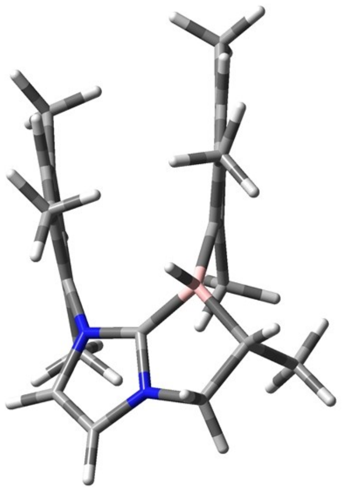
C	0.19529603	1.71204299	-0.17381900	
C	-1.54096497	3.12103199	-0.28014000	
N	-2.00610697	1.87112699	-0.07261200	
H	-1.08579397	-0.06938201	0.16069900	
H	1.22791603	1.40946599	-0.18721000	
N	-0.21042497	3.01621999	-0.36288400	
C	0.47646103	4.31564599	-0.51713200	
H	1.32308503	4.19339099	-1.19813500	
H	0.84430503	4.61915999	0.46660100	
C	-0.62884697	5.26376399	-1.03071200	
C	-0.64052297	5.29199899	-2.56071700	
H	-0.69624797	4.28109899	-2.97650400	
H	-1.51014797	5.84314699	-2.92657200	
B	-2.00249097	4.67176499	-0.30204800	
H	0.25951203	5.76494199	-2.96834300	
H	-0.41152497	6.27001599	-0.66257600	
C	-3.40937597	1.55555999	-0.07789500	
C	-4.23103197	2.11828699	0.90155500	
C	-3.92402797	0.79496899	-1.13394200	
C	-5.60796897	1.97136499	0.75538600	
C	-5.30651197	0.67234799	-1.22949100	
C	-6.16329597	1.28338099	-0.31652200	
H	-6.25994697	2.43614399	1.48864900	
H	-5.72470797	0.11081099	-2.05939100	
C	-3.51994397	4.97599799	-0.78310500	
C	-4.31709297	5.85579399	-0.01059300	
C	-4.17172497	4.29536299	-1.83234300	
C	-5.69227997	5.94521299	-0.21201800	
C	-5.55623997	4.39301799	-2.00546800	
C	-6.34139497	5.19168099	-1.18750300	
H	-6.27600897	6.62043799	0.40921700	
H	-6.02754497	3.82612799	-2.80538100	
C	-3.03706897	0.14276999	-2.16449200	
H	-2.19570297	0.77931299	-2.44223800	
H	-2.62810697	-0.80114401	-1.79269600	
H	-3.60985597	-0.07796301	-3.06553300	
C	-3.68521597	2.89113899	2.07048400	
H	-4.31067597	2.72261199	2.94824400	
H	-2.66055997	2.60546899	2.30990400	
H	-3.68804497	3.95930099	1.84469200	
C	-7.65438897	1.25034199	-0.51752600	
H	-7.96529897	2.13116999	-1.08790600	
H	-7.96464497	0.36299499	-1.07111300	
H	-8.18435497	1.26785799	0.43594800	

C	-3.70653797	6.73886299	1.05665900	
H	-2.76950897	7.18011999	0.71377900	
H	-4.39305297	7.54581299	1.31934400	
H	-3.47601697	6.18269699	1.97003600	
C	-3.42612097	3.47858099	-2.86336500	
H	-3.13649597	4.11421299	-3.70548800	
H	-2.51070897	3.03709599	-2.47314700	
H	-4.05616797	2.67614799	-3.25408100	
C	-7.83883597	5.26111099	-1.34805600	
H	-8.16438697	4.73056699	-2.24484800	
H	-8.34602797	4.81382299	-0.48781800	
H	-8.18034897	6.29616999	-1.42486800	
H	-1.88022497	4.93952399	0.88303900	

ΔE *trans*-**4a** vs *cis*-**4a** *=-2.989 Kcal.mol*$^{-1}$

- NHC-borane *trans*-**4d** (M062X/6-311g(d,p)) //pcm (ether)

Sum of electronic and zero-point Energies= -1102.844485 (Hartree/Particle)

Sum of electronic and thermal Energies= -1102.818077 (Hartree/Particle)

Sum of electronic and thermal Enthalpies= -1102.817133 (Hartree/Particle)

Sum of electronic and thermal Free Energies= -1102.900798 (Hartree/Particle)

C	-1.44054885	0.15243902	0.00000000	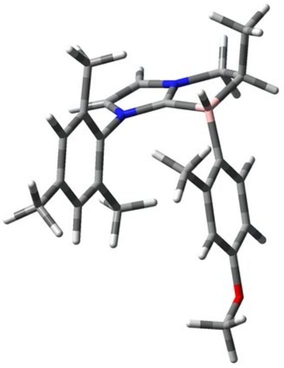
C	-0.84096785	-1.06512898	0.04126900	
C	-2.07096385	-0.84755998	1.90019000	
N	-2.19953585	0.26667202	1.15749600	
H	-1.39652985	0.94369002	-0.72908000	
H	-0.17253685	-1.54374698	-0.65317100	
N	-1.24613985	-1.65086898	1.22206100	
C	-1.05859685	-2.98433498	1.82085900	
H	0.00758215	-3.22518698	1.82240400	
H	-1.57563185	-3.71202798	1.19171200	
C	-1.66533385	-2.85814998	3.23876900	
C	-0.53869385	-2.66382198	4.25895600	
H	0.00916415	-1.73827398	4.04716800	
H	-0.94442985	-2.57256498	5.26836000	
B	-2.69145285	-1.53872398	3.22958200	
H	0.18211515	-3.48914098	4.25230100	
H	-2.19960785	-3.78443698	3.46943500	
C	-3.04516485	1.36900002	1.52083800	
C	-4.34567685	1.41145102	1.00979500	

C	-2.55306185	2.31710002	2.41643500	
C	-5.16410385	2.45558402	1.42965900	
C	-3.40837985	3.34917802	2.80103800	
C	-4.71351485	3.43012802	2.32161500	
H	-6.18309585	2.50488002	1.05774400	
H	-3.04842585	4.09618402	3.50120100	
C	-1.16108785	2.19635502	2.97633200	
H	-1.08506585	1.31225202	3.61604000	
H	-0.42129485	2.08823102	2.17977300	
H	-0.90843085	3.07500002	3.56878400	
C	-4.84449585	0.35132102	0.06508100	
H	-5.87894485	0.54924102	-0.21491000	
H	-4.24103785	0.31800002	-0.84564400	
H	-4.79828085	-0.63554398	0.53414500	
C	-5.62857985	4.54923002	2.74626200	
H	-5.20324885	5.11386902	3.57603300	
H	-5.80100485	5.24115602	1.91793100	
H	-6.60015885	4.15931502	3.05621900	
H	-2.52899985	-0.84326498	4.20907000	
C	-4.25663685	-1.82710998	2.90020800	
C	-5.30634085	-0.98981698	3.32281600	
C	-4.60915085	-2.88779998	2.05002800	
C	-6.61962185	-1.20425298	2.88217400	
C	-5.90508685	-3.11472198	1.60931000	
H	-3.83327185	-3.56853598	1.70872200	
C	-6.92282385	-2.25652298	2.02115600	
H	-6.15147985	-3.93920098	0.95036700	
H	-7.39855585	-0.53452598	3.22592500	
C	-5.06131185	0.16360002	4.26834700	
H	-4.65466285	-0.19503398	5.21699000	
H	-4.33680185	0.86964102	3.85834600	
H	-5.98784985	0.70438702	4.47142200	
O	-8.17188885	-2.52568298	1.54063900	
C	-9.22203285	-1.66740798	1.93787500	
H	-10.11965485	-2.04037598	1.44987200	
H	-9.36376485	-1.68733398	3.02281200	
H	-9.03572185	-0.63731198	1.61755700	

- NHC-borane *cis*-**4d** (M062X/6-311g(d,p)) //pcm (ether)

Sum of electronic and zero-point Energies= -1102.841889 (Hartree/Particle)

Sum of electronic and thermal Energies= -1102.815460 (Hartree/Particle)

Sum of electronic and thermal Enthalpies= -1102.814515 (Hartree/Particle)

Sum of electronic and thermal Free Energies= -1102.898764 (Hartree/Particle)

C	-1.37957323	0.00000000	0.00000000	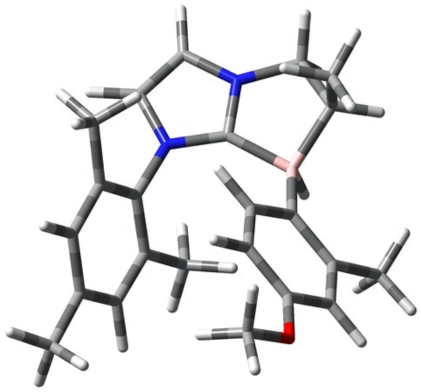
C	-2.35688723	-0.92453400	-0.17656100	
C	-0.47456523	-2.01646200	0.36247800	
N	-0.21844023	-0.69156200	0.32880500	
H	-1.39564023	1.07337700	-0.07835300	
H	-3.39490123	-0.81031600	-0.43674400	
N	-1.76524023	-2.14907700	0.03719000	
C	-2.24429023	-3.54430600	0.04082400	
H	-2.94489523	-3.68035300	-0.78701700	
H	-2.76445623	-3.72419600	0.98500500	
C	-0.94779023	-4.36942100	-0.06835700	
C	-0.57149323	-4.57541600	-1.53731500	
H	-0.42305323	-3.61217500	-2.04039100	
H	0.36939977	-5.12350100	-1.62061800	
B	0.16664777	-3.45417400	0.76481900	
H	-1.34067123	-5.12492900	-2.09075300	
H	-1.11422023	-5.34183600	0.40323500	
C	1.08283677	-0.10055700	0.47916900	
C	1.74410477	-0.19441300	1.70479800	
C	1.66798377	0.49960000	-0.64128700	
C	3.03048077	0.33539900	1.78764400	
C	2.95044977	1.02259000	-0.50401900	
C	3.64972377	0.94234800	0.69896200	
H	3.56132577	0.26668200	2.73207700	
H	3.42211977	1.48495900	-1.36552600	
C	1.72866277	-3.64374400	0.43711600	
C	2.55067277	-4.53195700	1.16909600	
C	2.35197377	-2.92445900	-0.58371100	
C	3.90628577	-4.64347600	0.88254800	
C	3.71339077	-3.02039300	-0.88233300	
C	4.49856877	-3.88732500	-0.13110100	
H	4.54013677	-5.32261300	1.44354300	
H	4.13159677	-2.42272600	-1.68183700	
C	0.96714177	0.54133500	-1.97553900	
H	0.46518677	-0.40569800	-2.19046600	
H	0.20722777	1.32597400	-2.00945800	
H	1.68627677	0.73883300	-2.77004100	
C	1.12443877	-0.87394000	2.89447200	
H	1.57868477	-0.50932000	3.81583400	
H	0.04713677	-0.70547100	2.94113000	
H	1.28242577	-1.95413900	2.83163500	
C	5.05797277	1.46581500	0.80907600	
H	5.77623977	0.66359400	0.61883500	

H	5.24250377	2.25912500	0.08378200	
H	5.25572077	1.85579500	1.80846500	
H	-0.07528723	-3.54768300	1.95893200	
H	1.75636177	-2.24430100	-1.18715300	
C	1.96993277	-5.37381800	2.28011700	
H	1.07654277	-5.90392700	1.94220600	
H	2.69505577	-6.10559700	2.63973700	
H	1.66363277	-4.74797500	3.12288100	
O	5.84000977	-4.07207600	-0.30849400	
C	6.46630077	-3.31665000	-1.32477400	
H	7.51805977	-3.59329100	-1.30564200	
H	6.04744577	-3.54951500	-2.30884000	
H	6.36920377	-2.24235200	-1.13756500	

ΔE *trans*-**4d** vs *cis*-**4d**= *-1,629 Kcal.mol^{-1}*

b. EPR Studies

EPR assays were carried out on a Bruker EMX spectrometer operating at X-band with 100 kHz modulation frequency and equipped with an NMR gaussmeter for magnetic field calibration. The following conditions were used: non-saturating microwave power, 15 mW; modulation amplitude, from 0.1 G to 1 G; receiver gain, 5x10^5; 1-4 scans. The media were prepared under argon in the appropriate deoxygenated solvent and then transferred into sealed NMR tubes. The samples were heated directly into the EPR cavity with the aid of a Bruker Eurotherm B-VT 2000 variable temperature unit using a N_2 stream cooling system. For all the paramagnetic species detected, the hyperfine coupling constant values were determined after standard simulations of the experimental EPR signals using the WinSim software.

- Origin of the diastereoselectivity under thermal equilibrium by EPR study and possible explanation of by-product **3a**.

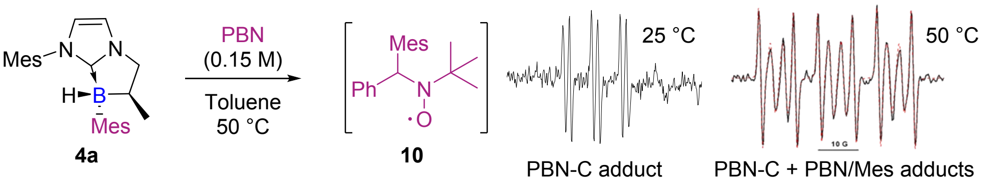

Procedure:

The spin trapping experiments were performed as follows. A 0.17 mol.L^{-1} **4a** solution was prepared under argon in deoxygenated toluene in the presence of the spin trap α−phenyl-N-terbutylnitrone (PBN, 0.15 mol.L^{-1}). The medium was transferred into the EPR cavity before

being heated to 323K. The signal observed is given below and its simulation (superimposed red dotted lines) revealed the presence of two radical spin adducts. The major species (~ 60%, a_N = 14.6 G and a_H = 8.5 G) was assigned to the spin adduct formed after addition of the mesityl radical on PBN (Mes/PBN spin adduct).

- Since nitroxides can be synthesized via Grignard reagents, the Mes/PBN nitroxide was also prepared by nucleophilic addition of MesMgBr to PBN: the same six-line spectrum was observed (100 %, a_N = 14.6 G and a_H = 8.5 G). (see for examples: (a) K. Hideg and L. Lex, *Perkin Trans 1*, 1986, **8**, 1431; (b) C. Sar, T. Kalai, J. Jeho K. Hideg, *Arkivoc*, 2012, 47).

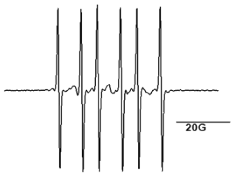

- The second species (~ 40%, a_N = 14.5 G and a_H = 3.2 G) correspond to the adduct of an unidentified carbon-centered radical on PBN. It should be mentioned that this latter species was also observed before heating, or when the same experiment was performed with **5a** instead of **4a**. On the other hand, the Mes/PBN spin adduct was never formed before heating the medium or starting from **5a**.

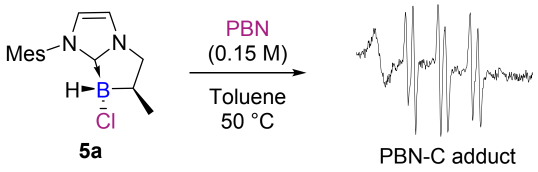

7. Mechanism studies

a. Ionic pathway

Borane **5a** was submitted to $MgBr_2$ (1.0 equiv) in benzene-d$_6$, the intermediate borenium was never observed by ^{11}B NMR, even in the presence of triethylphosphine oxide as borenium trap, and only the $MgBr_2$•OP(Et)$_3$ complex (^{31}P NMR; δ = 64.4 ppm) was observed. So, if the borenium was formed during our reaction, we should be able to see the phosphine oxide complex, which is not the case.

Procedures:

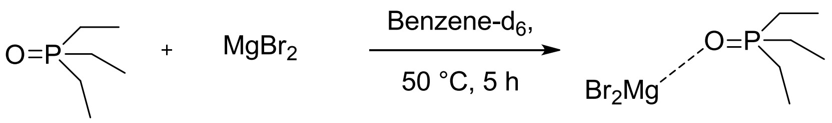

$MgBr_2$ (0.07 mmol, 1 equiv) and triethylphosphine oxide (0.07 mmol, 1 equiv) were dissolved in dry benzene-d$_6$ (0.4 mL) in a 10 mL Schlenk flask in glove box. The Schlenk was sealed and the reaction was stirred at 50 °C for 5 h.

^{31}P NMR

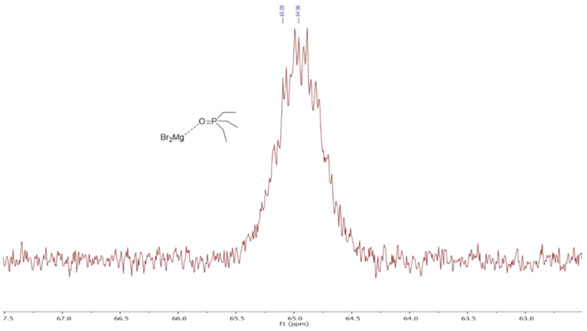

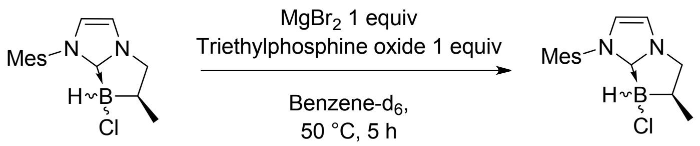

MesNHC-B(H)Cl **5a** (0.05 mmol, 1 equiv) and $MgBr_2$ (0.05 mmol, 1 equiv) were dissolved in dry benzene-d_6 (0.33 mL) in a 10 mL Schlenk flask in glove box. The reaction was heated at 50 °C then triethylphosphine oxide (0.05 mmol, 1 equiv) was added and the mixture was stirred at 50 °C for 5 h.

^{11}B NMR

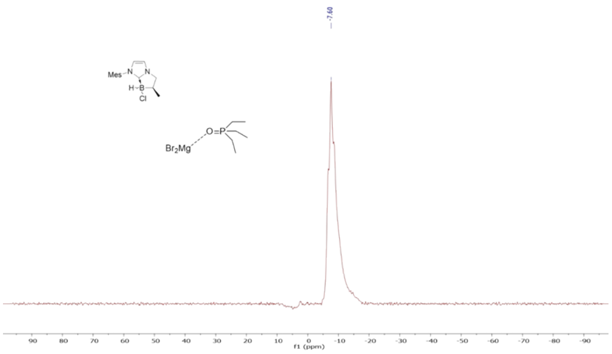

^{31}P NMR

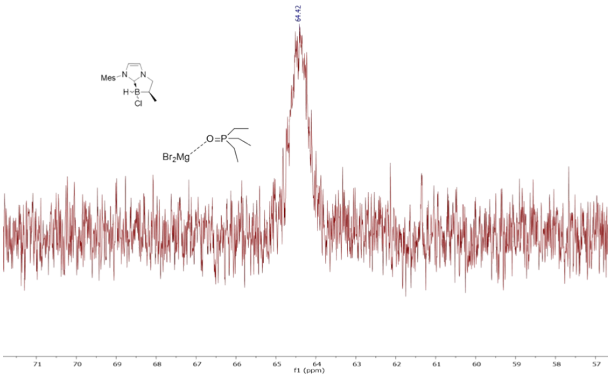

b. Radical pathway

- Reaction of **5a** with TEMPO:

Attempts to stop the reaction or trap the boryl radical with TEMPO failed. The reaction of **5a** with mesityl Grignard reagent was conducted with 1 to 5 equivalents of TEMPO, sole the rate of the reaction was affected (62% yield after 5 h instead of 93%) and no side-product was ever observed. It is in fact possible that the TEMPO-boryl adduct is unstable and the reaction is reversible and in fine goes unnoticed in the reaction.

- Synthesis of **6a**:

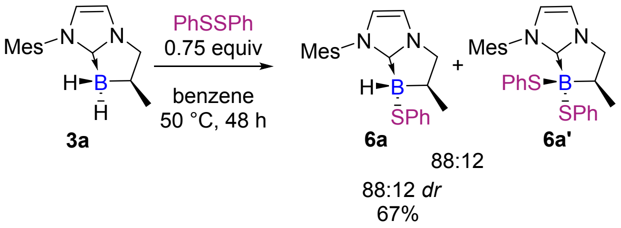

Procedure:

The NHC-borane **3a** (1.0 equiv) and the phenyldisulfide (0.75 equiv) were introduced in a Schlenk flask. Then deoxygenated benzene was introduced, the Schlenk was sealed and the solution was stirred at 50 °C for 72 hours under an aluminum foil. After evaporation of the solvent, the compound was purified by flash chromatography using pentane:DCM 1:1 then 100% DCM as eluent to afford **6a** in 67% yield as two diastereomers (88:12 *dr*).

^{1}H NMR (400 MHz, $CDCl_3$) δ (ppm) 7.05 (d, ^{3}J = 1.8 Hz, 1H), 7.04 – 6.95 (m, 2H), 6.89 (s, 1H), 6.85-6.77 (m, 4H), 6.63 (s, 1H), 4.31 (dd, ^{2}J = 11.1 Hz, ^{3}J = 7.4 Hz, 1H), 3.60 (dd, ^{2}J = 11.1 Hz, ^{3}J = 3.3 Hz, 1H), 2.27 (s, 3H), 2.04 (s, 3H), 1.91 - 1.80 (m, 4H), 1.18 (d, ^{3}J = 7.3 Hz, 3H).

^{13}C NMR (101 MHz, $CDCl_3$) δ (ppm) 142.3, 139.5, 135.4, 134.7, 133.7, 129.5, 129.3, 128.9, 127.6, 124.5, 121.9, 117.3, 58.1, 30.0 $_{(HSQC)}$, 21.1, 21.1, 17.9, 17.7, C_9 is not observed.

^{11}B NMR (128 MHz, $CDCl_3$) δ (ppm) -12.8 (d, $^{1}J_{BH}$ = 103.8 Hz).

HRMS (ES, $[M+Na]^+$) calcd for $[C_{21}H_{25}N_2SBNa]^+$: 371.1728; found: 371.1728

^{1}H NMR (400 MHz, $CDCl_3$)

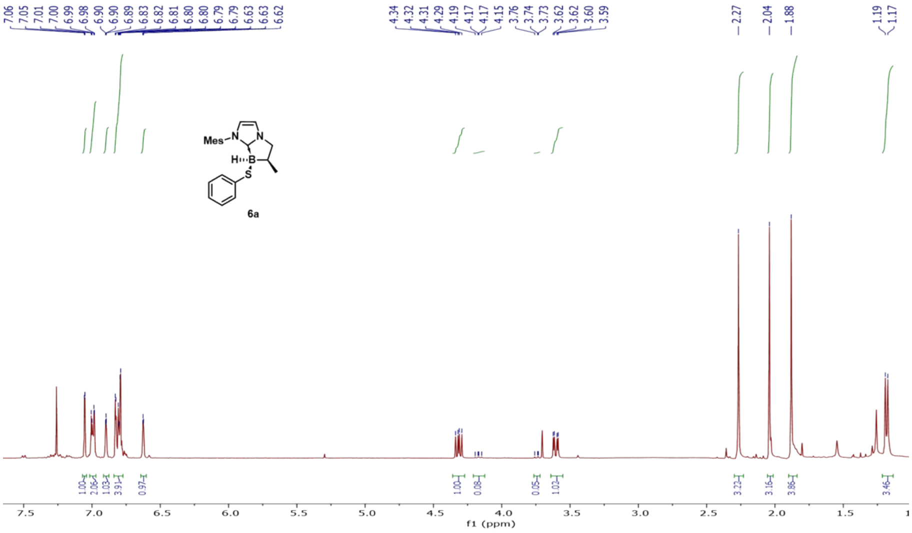

^{13}C NMR (101 MHz, $CDCl_3$)

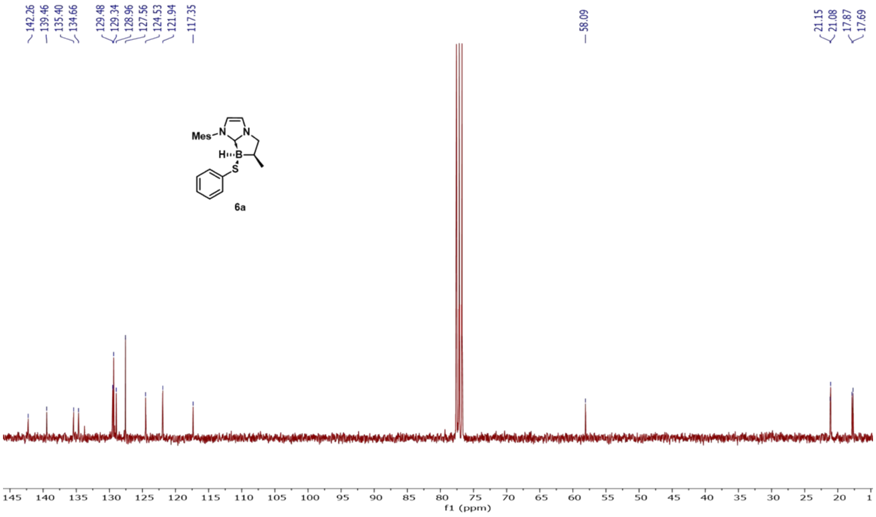

^{11}B NMR (128 MHz, $CDCl_3$)

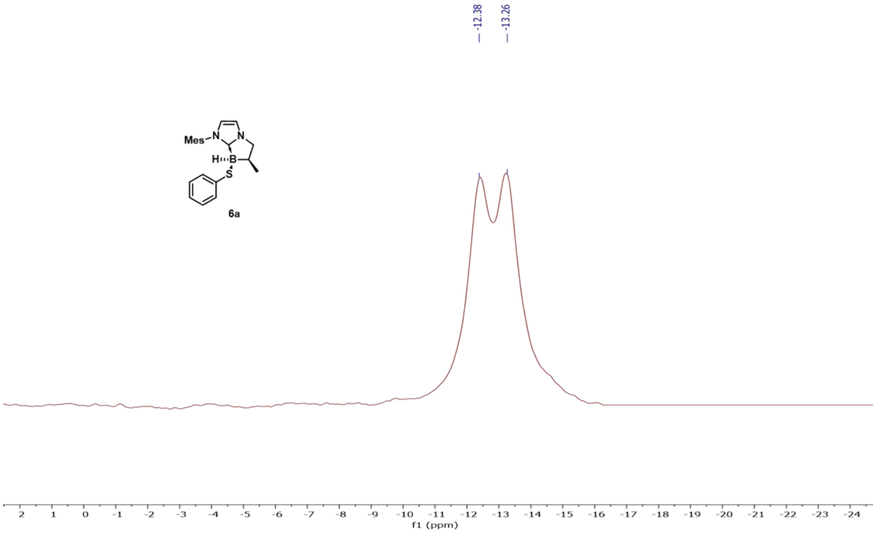

- Spin trapping experiment with **3a** with phenyldisulfide and α-phenyl-*N*-tertiary-butyl nitrone

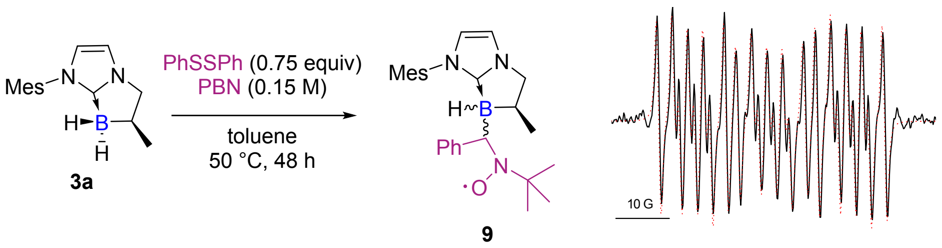

Procedure:

In a glovebox, NHC-borane **3a** (0.17 mmol, 1.0 equiv), PhSSPh (0.12 mmol, 0.75 equiv) and N-*tert*-Butyl-α-phenylnitrone (0.15 M) were dissolved in dry benzene (3 mL) in a 10 mL Schlenk flask.

- Indirect trap of **IIa** from **5a:**

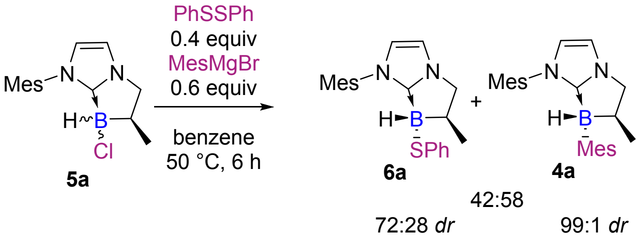

Procedure:

NHC-borane **5a** (0.07 mmol, 1 equiv) and PhSSPh (0.03 mmol, 0.4 equiv) were dissolved in dry benzene (0.44 mL) in a 10 mL Schlenk flask under argon. Then, 0.6 equivalent of Grignard reagent MesMgBr (0.04 mmol, 0.6 equiv) was added via a syringe at room temperature. The Schlenk was sealed and the reaction was stirred at 50 °C for 6 h. The crude product was filtered through a short plug of silica gel using dichloromethane (10 mL) as eluent to give **4a** and **6a** in 58:42 ratio.

^{1}H NMR (400 MHz, CD_2Cl_2)

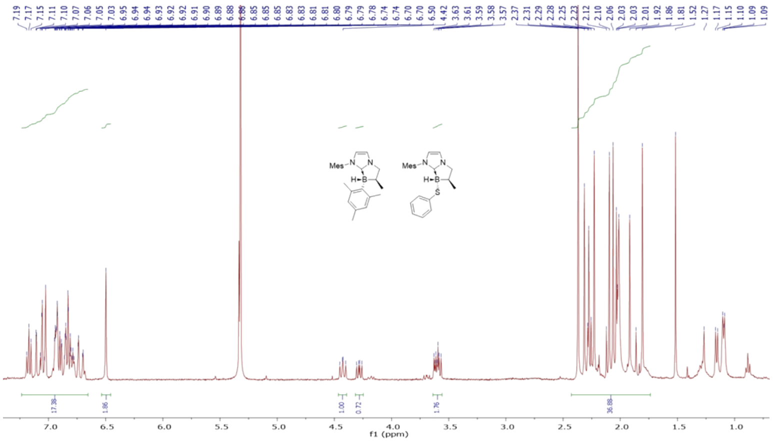

^{11}B NMR (128 MHz, CD_2Cl_2)

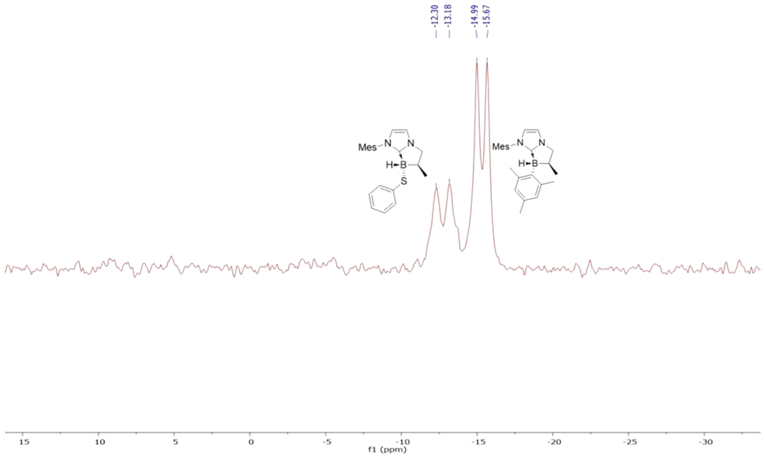

- Stability of final product **4a** under reductive conditions and possible explanation for bis-functionalized adducts:

The stability of the final product **4a** was tested in presence of an excess of MesMgBr to see if it was able to abstract the last hydride.

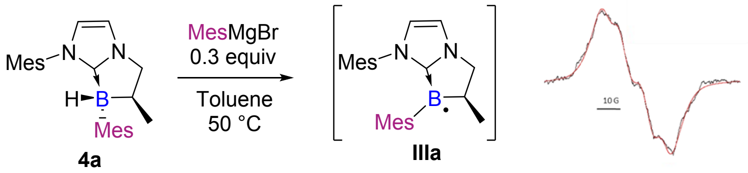

Procedure:

A medium containing NHC-borane **4a** (1.0 equiv) and MesMgBr (0.3 equiv) was prepared in toluene (0.2 M) and submitted to EPR analysis. No signal was observed before heating. Raising the temperature to 323 K yielded an intense and wide signal.

8. Catalysis

a. Imine reduction with silane catalyzed by chiral NHC-borenium

Procedure:

In a glovebox, a solution of trityl borate (0.017 mmol, 1 equiv) in CH_2Cl_2 (0.15 mL) was added to a solution of NHC-borane **4j** (0.017 mmol, 1 equiv) in CH_2Cl_2 (0.15 mL) at room temperature to afford the corresponding NHC-borenium. To a solution of NHC-borenium (20 mol%, 0.017 mmol) in CH_2Cl_2 (0.7 mL) was added phenylsilane (0.085 mmol, 1 equiv). After 10 minutes stirring at room temperature the imine (0.085 mmol, 1 equiv) was added in one portion. The reaction was monitored by TLC using pentane:DCM 7:3 as eluent. After 20

hours, 2 mL of a 1:1 MeOH/ KOH (2.0 M) was added dropwise and the solution was left to stir for 1 h. The solution was then diluted with H_2O (5 mL) then extracted with CH_2Cl_2 (3 x 10 ml). The organic layer was concentrated *in vacuo* and purified by silica gel column chromatography using pentane:DCM 7:3 as eluent to afford the pure amine.

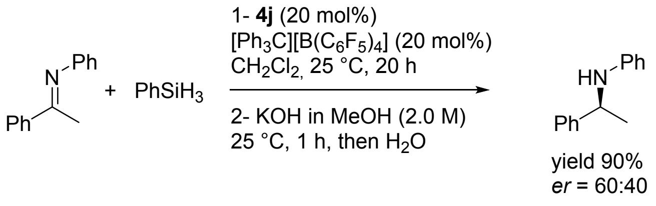

b. Borenium **IVa**-Silane complex studies

General procedure:

In a glovebox, NHC-borane **4a** (0.03 mmol, 1.0 equiv) and trityl tetrakis (pentafluorophenyl)borate (0.03 mmol, 1.0 equiv) were mixed in CD_2Cl_2 (0.2 mL). The mixture was stirred at rt for 1 h, then silane (0.015 mmol, 0.5 equiv) was added, then the reaction was followed by ^1H and ^{11}B NMR.

- with $PhSiH_3$:

The expected complex of phenylsilane and borenium species **IVa** was not detected and only the characteristic signals of the borenium precursor **IVa**, were confirmed by ^1H and ^{11}B NMR.

^1H NMR (400 MHz, CD_2Cl_2)

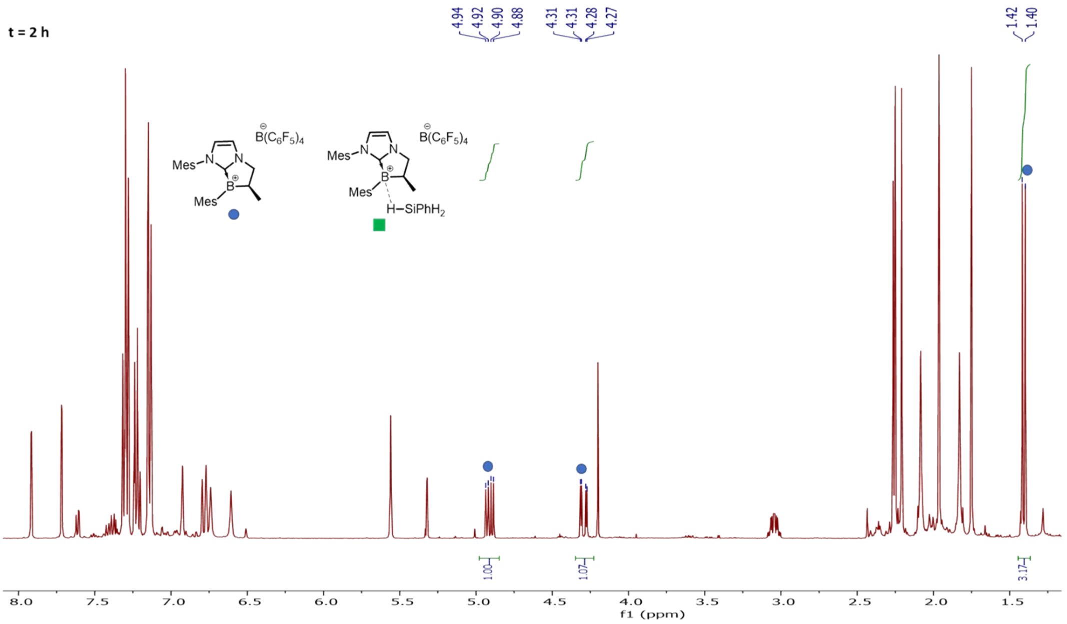

^{11}B NMR (128 MHz, CD_2Cl_2)

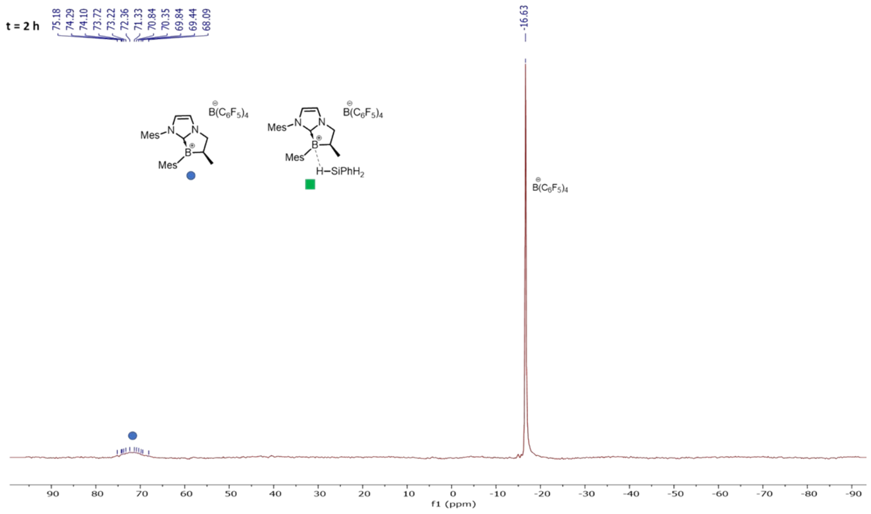

^{1}H NMR (400 MHz, CD_2Cl_2)

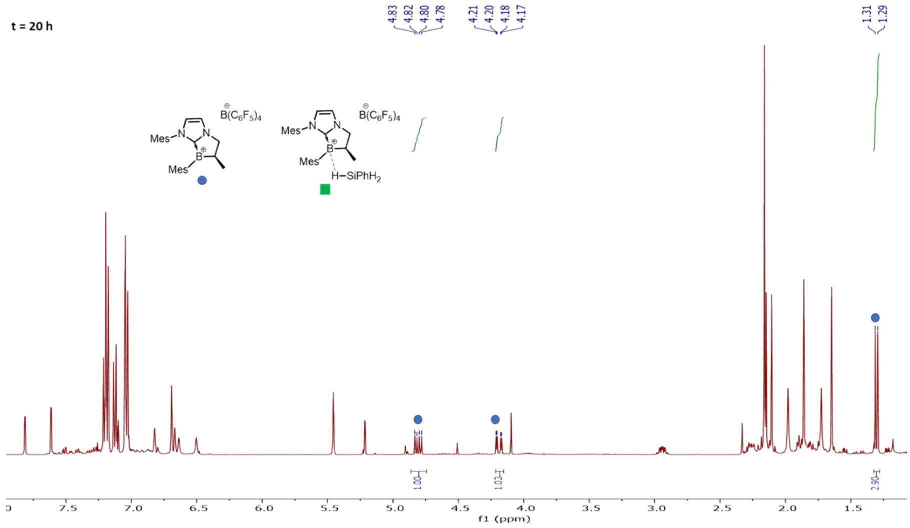

^{11}B NMR (128 MHz, CD_2Cl_2)

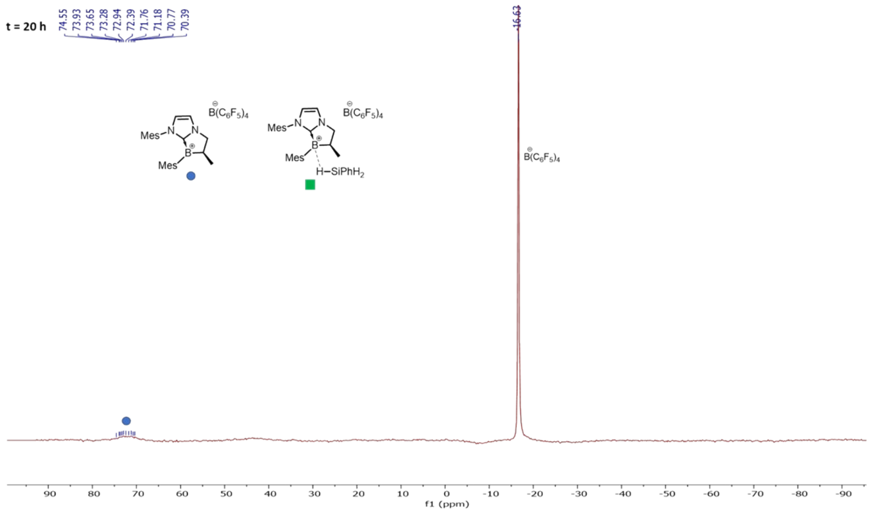

- with Et_3SiH:

After 2 h at rt, a second species appeared, corresponding to a hydrido complex where the hydride atom is partitioned between the silicon and the boron atom. This was revealed by the ^1H NMR chemical shifts of the characteristic diastereotopic CH_2 protons of the boracyclic moiety values which were found as intermediary values between borane **4a** and borenium **IVa** species. The same tendency was observed in ^{11}B NMR, where the borenium **IVa** and the complex borenium-silane co-exist (δ = 71 and 44 ppm respectively). Finally, only one diastereomer of the hydrido complex was detected by ^1H NMR after 20 h, confirming the results previously obtained with borenium **4a** and $LiBH_4$.

^1H NMR (400 MHz, CD_2Cl_2)

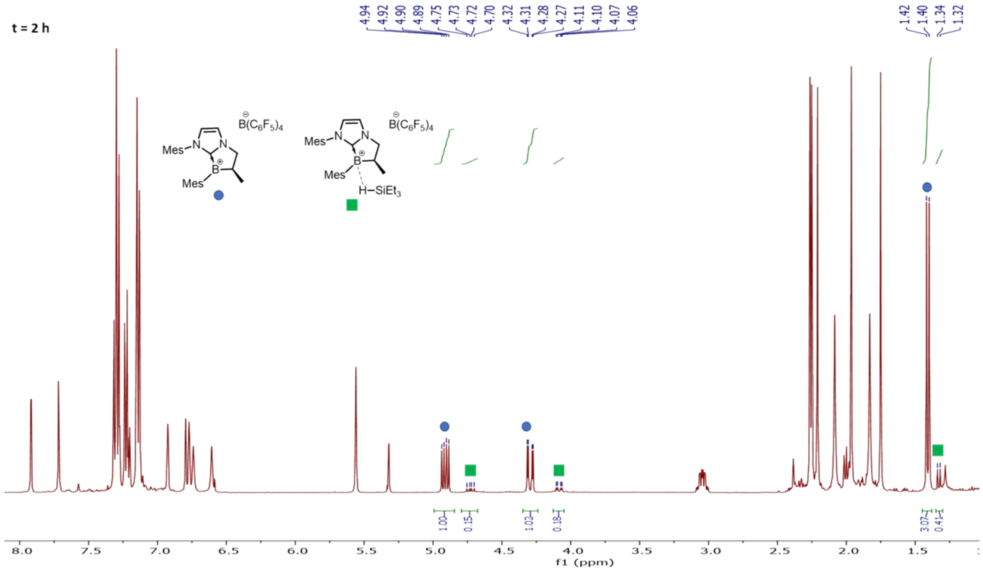

^{11}B NMR (128 MHz, CD_2Cl_2)

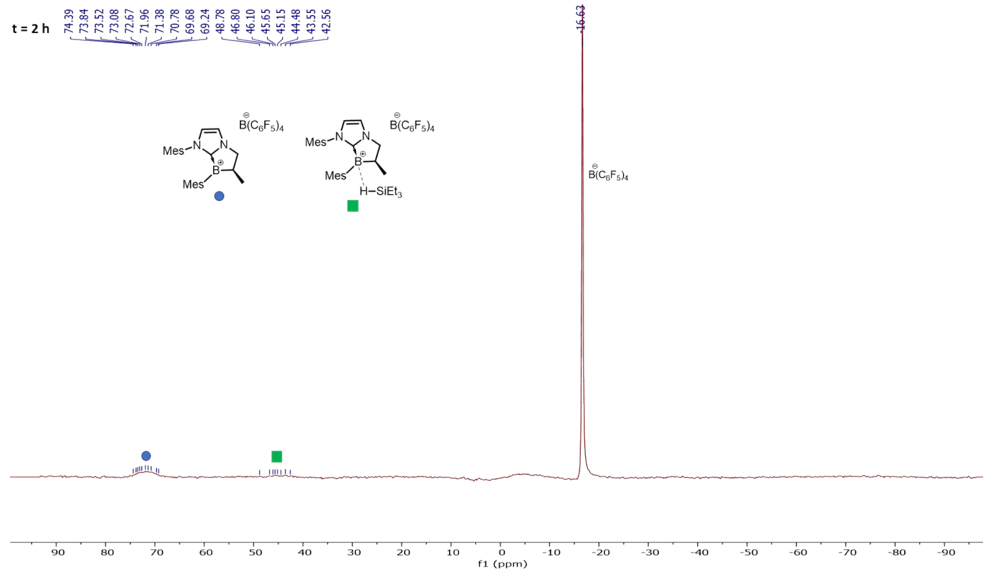

^{1}H NMR (400 MHz, CD_2Cl_2)

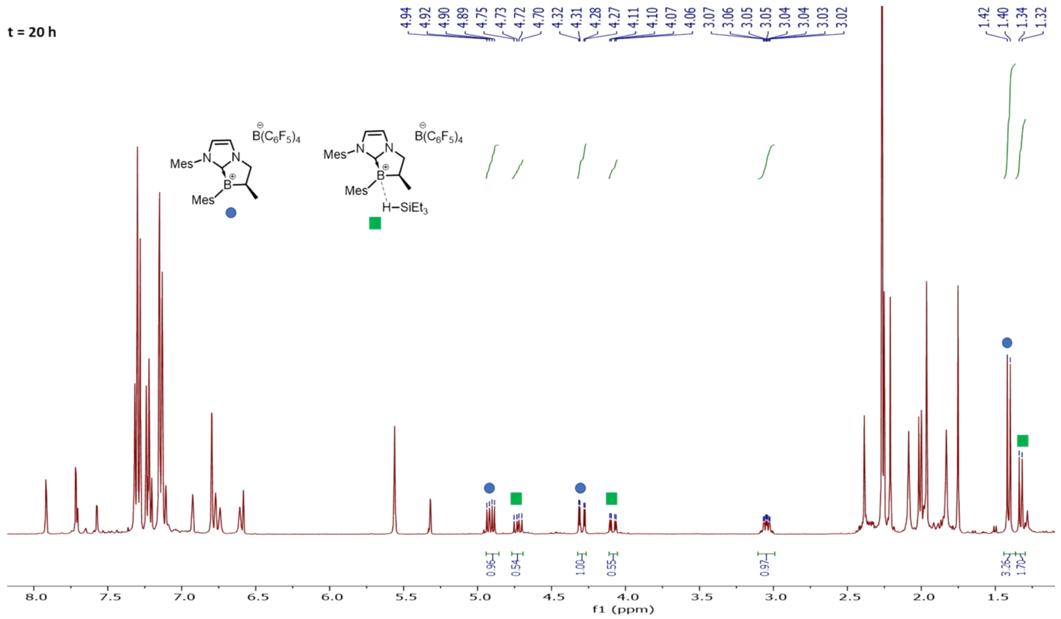

^{11}B NMR (128 MHz, CD_2Cl_2)

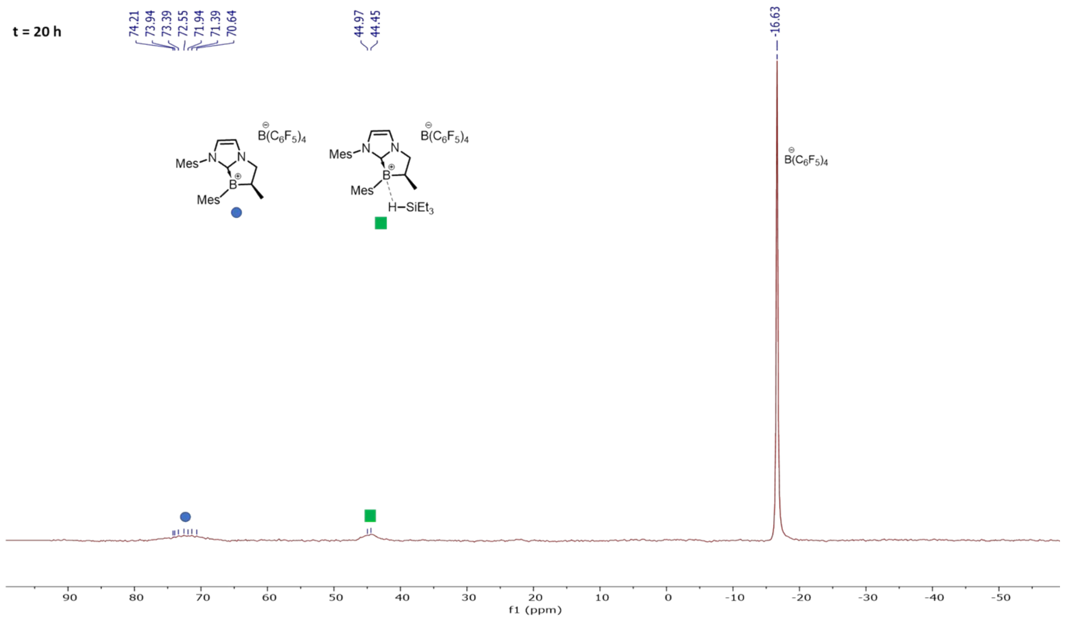

9. Cristal structures

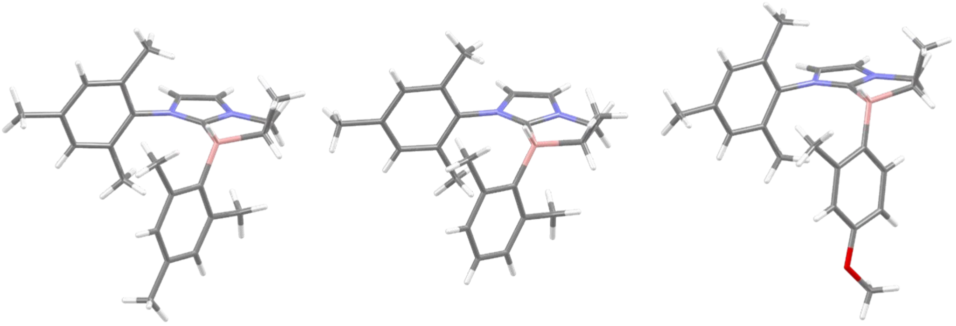

Compounds	**4a**	**4d**	**4b**
Identification code	ca493d	ca582d	ca609d
Empirical formula	$C_{24}H_{31}BN_2$	$C_{23}H_{29}BN_2$	$C_{23}H_{29}BN_2O$
Formula weight	358.32	344.29	360.29
Crystal system	orthorombic	orthorombic	orthorombic
Space group	$P2_12_12_1$	$P2_12_12_1$	$P2_12_12_1$
a (Å)	7.55860 (10)	8.56120 (10)	7.76550(10)
b (Å)	9.20850 (10)	15.4461(2)	12.17080(10)
c (Å)	30.8403 (5)	15.9652(3)	22.3907(2)
α (°)	90	90	90
β (°)	90	90	90
γ (°)	90	90	90
V $(Å)^3$	2146.59 (5)	2111.19(5)	2116.20(4)
Z	4	4	4
T (K)	293	293	293
Density	1.109	1.083	1.131
F (000)	776.0	744.0	776.0
Crystal size	0.34x0.24x0.22	0.32x0.28x0.2	0.4x0.18x0.18
Theta range for data collection	10.024 to 141.852	7.964 to 142.106	7.898 to 148.88
Index ranges	$-9 \le h \le 9$, $-11 \le k \le 9$, $-23 \le l \le 37$	$-10 \le h \le 9$, $-18 \le k \le 14$, $-17 \le l \le 19$	$-9 \le h \le 9$, $-14 \le k \le 14$, $-27 \le l \le 27$
Reflections collected	7385	7375	24338
Independent reflections	4047 [R_{int} = 0.0112, R_{sigma} = 0.0157]	3980 [R_{int} = 0.0128, R_{sigma} = 0.0170]	4258 [R_{int} = 0.0276, R_{sigma} = 0.0140]
Completeness to theta = 28.7 °	1.68/0.97	1.70/0.97	1.77/0.99
Absorption correction	Multi-Scan	Multi-Scan	Multi-Scan
Data / restraints / parameters	4047/0/251	3980/0/241	4258/0/251
Goodness-of-fit on F2	1.146	1.083	1.040
Final R indexes [I>=2σ (I)]	R_1 = 0.0420, wR_2 = 0.1196	R_1 = 0.0455, wR_2 = 0.1090	R_1 = 0.0364, wR_2 = 0.1027
Final R indexes (all data)	R_1 = 0.0429, wR_2 = 0.1205	R_1 = 0.0465, wR_2 = 0.1107	R_1 = 0.0371, wR_2 = 0.1040
Largest diff. peak and hole	0.13/-0.15	0.18/-0.33	0.15/-0.21
CCDC	1856403	1857909	1857908
